# Recent Advances in Synthetic Isoquinoline-Based Derivatives in Drug Design

**DOI:** 10.3390/molecules30244760

**Published:** 2025-12-12

**Authors:** Łukasz Balewski, Anita Kornicka

**Affiliations:** Department of Chemical Technology of Drugs, Faculty of Pharmacy, Medical University of Gdańsk, 80-416 Gdańsk, Poland; anita.kornicka@gumed.edu.pl

**Keywords:** isoquinoline derivatives, benzo[*c*]pyridine derivatives, biological properties, pharmacological activity

## Abstract

Compounds based on an isoquinoline scaffold (benzo[*c*]pyridine) display a broad spectrum of biological activities. In recent years, studies have focused mainly on their anticancer properties. Their antiproliferative effects are associated with diverse mechanisms that include targeting PI3K/Akt/mTOR signaling pathways and reactive oxygen species or inducing apoptosis and cell cycle arrest. Furthermore, isoquinolines may inhibit microtubule polymerization, topoisomerase, or tumor multidrug resistance. Recent studies have also shown that these compounds may act as effective antimicrobial, antifungal, antiviral, and antiprotozoal agents. Moreover, it has also been demonstrated that isoquinoline derivatives exhibit potent anti-Alzheimer effects, alleviating central nervous system functions. Additionally, they possess anti-inflammatory and antidiabetic properties. Due to the presence of donor nitrogen, the isoquinoline core constitutes an appropriate ligand that may be employed for the development of metal complexes with improved pharmacological properties. A number of chelates containing copper, iridium, or platinum were found to exhibit prominent biological activity, which places them in a leading position for the development of effective medications. This review summarizes the recent development of synthetic isoquinoline-based compounds with proven pharmacological properties in the period of 2020–2025. Also, other biomedical applications for synthetic isoquinoline derivatives are provided.

## 1. Introduction

**Figure 1:**
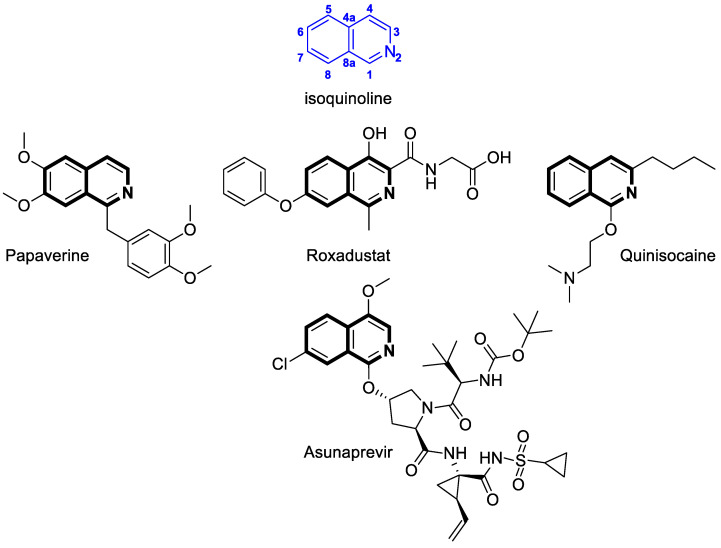
Chemical structures of isoquinoline (benzo[*c*]pyridine), natural alkaloid—Papaverine, and synthetic isoquinoline-based drugs—Roxadustat, Quinisocaine, and Asunaprevir.

The isoquinoline or benzo[*c*]pyridine scaffold consists of fuses of two aromatic rings—benzene and pyridine (Figure 1)—also known as 2-azanaphthalene, 2-benzazine, or 3,4-benzopyridine. In nature, this nitrogen-containing carbocyclic ring can be found as the structural part of an extremely large group of compounds—alkaloids—mostly occurring in high plants in several families, including *Ranunculacae*, *Berberidacae*, and *Papaveracae* [1]. Therefore, alkaloids containing an isoquinoline scaffold as a basic structural motif are extensively studied compounds with diverse biological activities that possess anticancer and anti-infective potential [2,3,4,5,6,7,8,9,10]. An example of an isoquinoline-based alkaloid is 1-[(3,4-dimethoxyphenyl)methyl]-6,7-dimethoxyisoquinoline—Papaverine (Figure 1)—extracted from *Rauwolfia serpentina* and *Papaver somniferum* [11]. This heteroaromatic system may be foreseen as a promising scaffold for the design and synthesis of pharmacologically active compounds [12]. Since this privileged structural framework occupies a prominent place in the medicinal chemistry field, a wide range of drugs are based on isoquinoline, including agents used in hypertension, anemia, smooth muscle spasms, or viral infections.

Recent advances in the synthesis of nitrogen-containing heterocycles have led to the development of novel isoquinoline derivatives. Such synthetic isoquinoline-based compounds are endowed with great therapeutic potential. For example, anemia associated with chronic kidney disease can be treated with an isoquinoline-based compound—2-[(4-hydroxy-1-methyl-7-phenoxyisoquinolin-3-yl)formamido]acetic acid—a first-of-its-kind hypoxia-inducible factor prolyl hydroxylase inhibitor, recently approved by the European Commission under the tradename Roxadustat (Figure 1) [13,14,15]. Isoquinoline also constitutes the framework of a topically used anesthetic agent—{2-[(3-butylisoquinolin-1-yl)oxy]ethyl}dimethylamine (Quinisocaine, Figure 1)—and a potent hepatitis C virus (HCV) NS3 protease inhibitor—Asunaprevir (BMS-650032) (Figure 1) [16].

**Figure 2:**
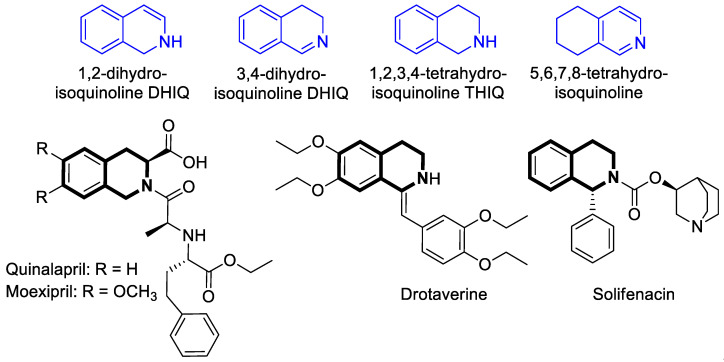
1,2- or 3,4-dihydroisoquinoline, 1,2,3,4-tetrahydroisoquinoline, and 5,6,7,8-tetrahydroisoquinoline cores—synthetic drugs containing the 1,2,3,4-tetrahydroisoquinoline core: Quinalapril, Moexipril, Drotaverine, and Solifenacin.

Structurally close to the isoquinolines are the 1,2- or 3,4-dihydroisoquinolines (1,2-DHIQs or 3,4-DHIQs) that contain scaffolds with two hydrogen atoms added [17,18,19]. They constitute key intermediates or building blocks in the synthesis of several isoquinoline-containing products such as tetrahydroisoquinolines (THIQs) (Figure 2). In light of the literature, THIQs may possess interesting biological properties and may constitute potential therapeutic agents [20,21,22,23,24,25]. For example, the THIQ core is a significant component of angiotensin inhibitors approved for use in hypertension, such as Quinalapril (Figure 2) [26] and Moexipril (Figure 2) [27]. Drotaverine—a selective inhibitor of phosphodiesterase-4, commonly used in clinical practice as an antispasmodic agent—also contains THIQ core (Figure 2) [28]. Another example constitutes a competitive long-lasting muscarinic receptor antagonist Solifenacin, indicated to treat an overactive bladder (Figure 2) [29].

**Figure 3:**
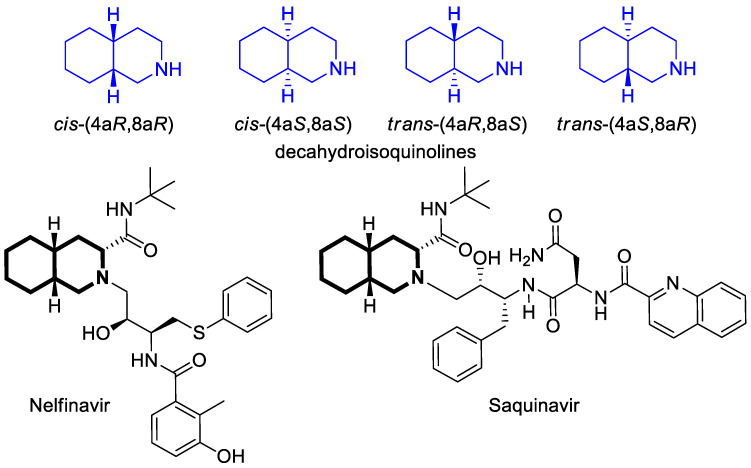
*Cis*- and *trans*-deca(per)hydroisoquinolines and antiviral agents based on their core.

The fully saturated isoquinoline analog, 1,2,3,4,4a,5,6,7,8,8a-decahydroisoquinoline (decahydroisoquinoline), forms two *cis*-isomers (4a*R*,8a*R* and 4a*S*,8a*S*) and two *trans*-isomers (4a*R*,8a*S* and 4a*S*,8a*R*) [30,31,32]. Numerous pharmaceutical products incorporate a decahydroisoquinoline ring system, including the protease inhibitors for Human Immunodeficiency Virus Type 1 (HIV-1), Nelfinavir and Saquinavir, as shown in Figure 3.

**Figure 4:**
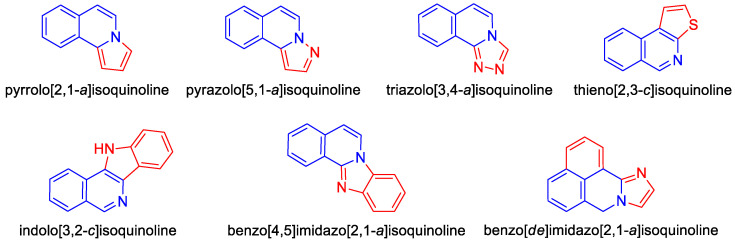
Examples of isoquinoline-based fused systems.

Special attention should be paid to derivatives where the isoquinoline system is combined with other heterocyclic or aromatic rings, resulting in more complex molecular structures. These fused isoquinoline derivatives such as pyrrolo[2,1-*a*]isoquinoline, pyrazolo[5,1-*a*]isoquinoline, 1,2,4-triazolo[3,4-*a*]isoquinoline, thieno[2,3-*c*]isoquinoline, 11*H*-indolo[3,2-*c*]isoquinoline, benzo[4,5]imidazo[2,1-*a*]isoquinoline, and 7*H*-benzo[*de*]imidazo[2,1-*a*]isoquinoline are of significant interest in medicinal chemistry (Figure 4).

They are abundantly found in nature as plant or marine alkaloids. To date, due to the diverse and significant biological properties of isoquinoline-based compounds, a growing number of articles has been published. The aim of this review is to provide insight into the papers filed in the years 2020–2025 and to consider isoquinoline-, tetrahydroisoquinoline-, and dihydroisoquinoline-containing compounds and metal complexes with ligands bearing isoquinoline scaffolds with proven biological properties. Special attention is paid to agents that may be used as potential lead structures in drug design, or for which practical applications may be found.

## 2. Chemotherapeutic Activity

### 2.1. Anticancer Agents

**Figure 5:**
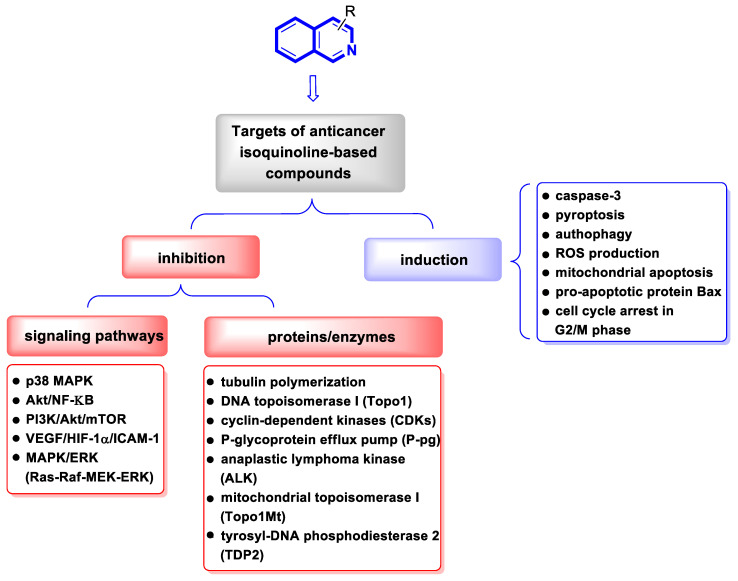
Possible modes of anticancer action of isoquinoline-based compounds.

Although cancer therapeutics have advanced dramatically, the limitations of traditional anticancer treatments are due to chemoresistance. Hence, antitumor drugs are mostly ineffective against rapidly dividing cancer cells. Consequently, designing new antitumor drugs still remains one of the most important areas of drug development [33]. Studies on the molecular mechanisms involved in tumor pathogenesis and progression have led to the identification of key tumor signal transduction pathways such as PI3K/Akt/NF-κB or Ras-Raf-MEK-ERK and p38 MAPK, which become a promising therapeutic option for several types of malignant diseases. Another therapeutically useful anticancer strategy consists of interactions with several proteins, e.g., topoisomerase or tyrosyl-DNA phosphodiesterase 2 (TDP2) or targeting microtubules (Figure 5).

In recent studies, there has been a growing body of evidence that semi-synthetic or synthetic isoquinoline-based compounds are promising anticancer drug candidates, since they interact strongly with the above-mentioned targets.

#### 2.1.1. Isoquinoline-Based Compounds

**Figure 6:**
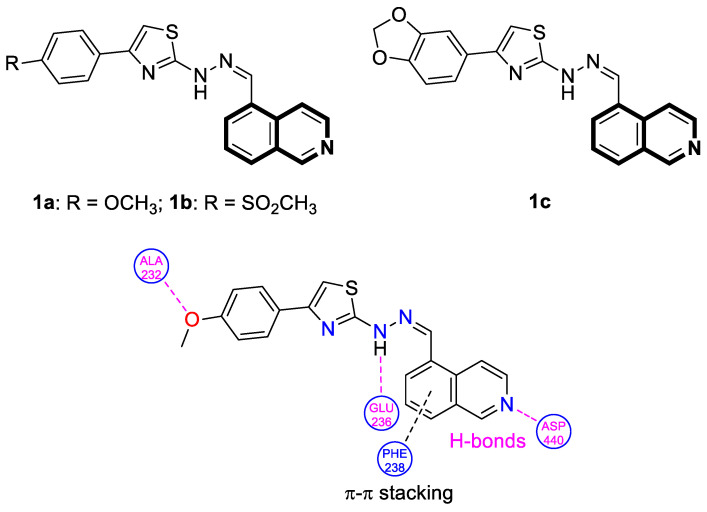
Designed isoquinoline–hydrazinyl-thiazole hybrids **1a**, **1b**, and **1c** with anticancer activity and in silico molecular docking simulations for 2-(2-(isoquinolin-5-ylmethylene)hydrazinyl)-4-(4-methoxyphenyl)thiazole (**1a**) and serine/threonine kinase Akt.

The serine/threonine kinase Akt, also known as protein kinase B, plays a crucial role in numerous cellular processes as a key component of the phosphoinositide-3-kinase (PI3K)-Akt signaling pathway. The dysregulation of Akt activity has been linked to several diseases, including cancer, diabetes, cardiovascular, and neurological disorders. In particular, excessive Akt activation affects a wide array of downstream targets and regulates multiple pathways that drive tumor development, such as those controlling cell growth, survival, and proliferation. Therefore, the inhibition of Akt or its associated routes using natural or synthetic compounds has gained significant attention as a promising strategy for cancer prevention and treatment [34]. The prominent anti-non-small-cell lung cancer (NSCLC) activity of 4-(naphthalen-2-yl)-2-[2-((quinolin-4-yl)methylene)hydrazinyl]thiazole prompted Orujova and co-workers to design a new class of anticancer agents via the molecular hybridization strategy of a bioisosteric isoquinoline scaffold with thiazole and hydrazone moieties. The designed hybrids were screened for their cytotoxic effects on human lung adenocarcinoma (A549) and mouse embryonic fibroblast (L929) cell lines [34]. Three compounds, **1a**, **1b**, and **1c**, depicted in Figure 6, demonstrated in vitro selective anti-A549 activity with assessed IC_50_ values of 1.43, 1.75, and 3.93 µM, respectively. Their calculated selectivity index values were SI = 20.28, >28.57, and 11.20, respectively.

Compound **1b**, bearing a 4-methylsulfonyl group, and compound **1c**, containing a 3,4-methylenedioxy moiety, were more efficacious than Cisplatin (IC_50_  =  3.90 µM), whereas 4-methoxy-substituted compound **1a** displayed a potency comparable with a reference drug. The synthesized compounds in A549 cells induce apoptosis in the early and late stages. Furthermore, it was found that compound **1a** in the A549 cell line showed a stronger Akt-inhibiting activity than GSK690693—an ATP-competitive pan-Akt inhibitor (IC_50_ = 3.55 µM vs. 4.93 µM). The authors supported this through molecular docking studies that provided insight into its binding mode. Induced-fit docking studies showed the binding pose of compound **1a** and provided a detailed understanding of the **1a** binding mechanism (Figure 6).

Compound **1a** is represented by a distinctive binding geometry, which creates interactions mainly at the entrance site. In the tight and deep hydrophobic pocket, the compound extends along the narrow binding cavity. The hydrogen bond and π–π interaction that stabilize the **1a**-Akt complex are observed at the isoquinoline site. Alanine 232 residue creates an additional hydrogen bond. Moreover, this compound forms a hydrogen bond with negatively charged glutaminic acid 236. The obtained results indicate that compound **1a** may serve as a promising anti-NSCLC agent, displaying selective anticancer activity by suppressing Akt in A549 cells. Unlike **1a**, derivatives **1b** and **1c** did not exhibit Akt-inhibiting activity at their IC_50_ concentrations, indicating that their anticancer properties in A549 cells are associated with a different signaling route.

As cancer cells rely upon high levels of copper to promote angiogenesis, tumor growth, and metastasis, the binding of this transition metal is intriguing from an anticancer therapy perspective. It has been demonstrated that several small organic compounds can disrupt copper homeostasis in cancer, either by chelating copper or increasing its intracellular levels [35,36,37].

**Figure 7:**
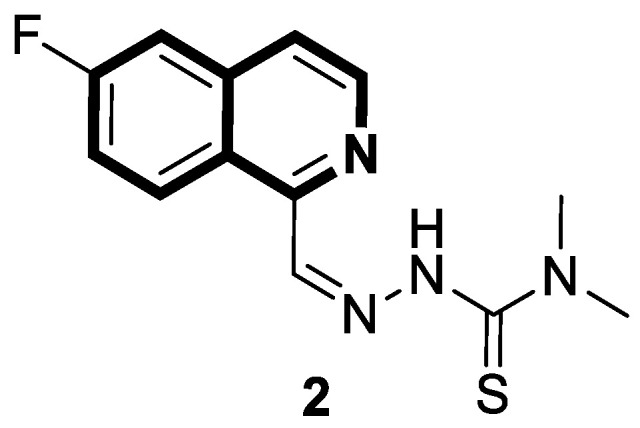
Highly potent anticancer thiosemicarbazone **2**.

Sun and co-workers rediscovered isoquinoline-based α-*N*-heterocyclic carboxaldehyde thiosemicarbazones (HCTs) as methylated and fluorinated antiproliferative agents, whose activities were potentiated by physiologically relevant Cu^2+^ levels [38]. Their cytotoxic studies identified a highly potent 2-((6-fluoroisoquinolin-1-yl)methylene)-*N*,*N*-dimethylhydrazinecarbothioamide (**2**) (Figure 7) in a panel of pancreatic cancer, small-cell lung carcinoma, prostate cancer, and leukemia, with IC_50_ values in the low-to-mid nanomolar range.

The lead compound **2** was tested against several aggressive cancer models and non-cancerous cell lines to determine whether its activity varies among cancers that require high copper levels for growth, such as PDAC, SCLC, and prostate cancer. Moreover, **2** was also evaluated in an aggressive leukemia model to prove its potency in the treatment of hematological malignancies such as acute myeloid leukemia (AML). The in vitro study showed that the non-cancerous human epithelial cell line (HPDE) was significantly more resistant to **2** in comparison to the tested tumor cell lines. Compound **2**—as a highly potent cancer cell growth inhibitor—was shown to possess a high degree of cancer-specific cytotoxicity, and its activity is dependent strongly on specific cancer characteristics. Calculated IC_50_ values were in the range of 1 nM to 200 nM. These results were supported by computational calculations that indicated that fluorination at the 6-position of the isoquinoline ring is beneficial for ligand–copper interactions and the formation of stable chelates. Further in vitro assays have shown that, in cancer cells, **2** induces oxidative stress, ROS production, has selective mitochondrial-dependent toxicity, and targets oxidative phosphorylation (OXPHOS). Compound **2** chelated with copper displayed in vivo effectiveness and tolerability in aggressive models of systemic leukemia—a murine BCR-ABL—expressing *Arf*-null pre-B, and a human systemic acute myeloid leukemia (AML) model (MV4-11). These cancers are characterized by aggressive phenotypes with high intrinsic OXPHOS levels. After 14 and 19 days of treatment, there was a significant reduction in the systemic disease burden for mice treated with the tested **2** copper(II) complex.

The anticancer mechanism of pyrrolo[2,1-*a*]isoquinoline-fused marine alkaloids—lamellarins—includes the inhibition of DNA topoisomerase I (Topo1), cyclin-dependent kinases (CDKs), mitochondrial topoisomerase I (Topo1Mt), and the induction of mitochondrial apoptosis. For this reason, there has been considerable interest in novel analogs of lamellarins. The DNA-Topo1 complex, also known as the cleavage complex, is stabilized by lamellarins, which block the religation step. A key enzyme for DNA replication, Topo1 catalyzes the transition from supercoiled DNA to relaxed DNA by creating a transient break on one strand of the DNA duplex, then sealing the nick by rejoining the cleaved strand. In order to design topoisomerase I inhibitors, scaffolding hopping or lead hopping has been applied to the design of new benzo[6,7]indolo[3,4-*c*]isoquinolines.

**Figure 8:**
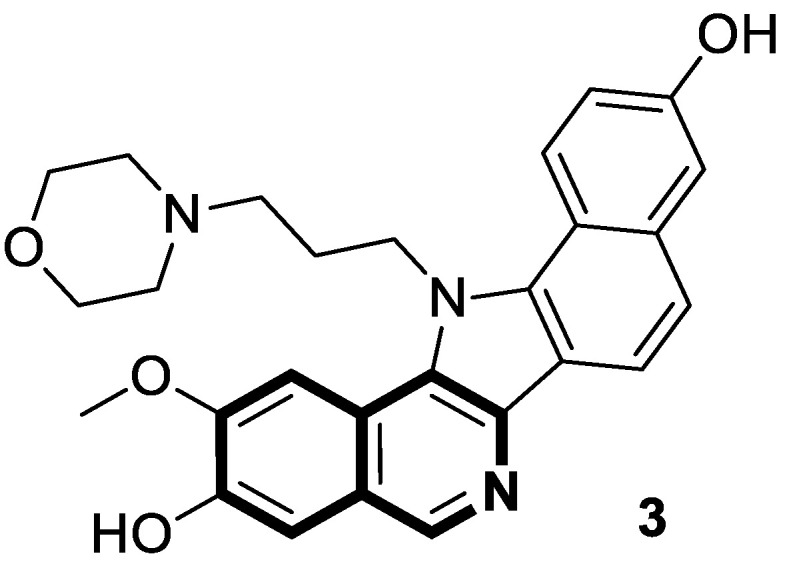
*N*-(3-morpholinopropyl)-substituted isoquinoline **3**, with antiproliferative activity.

Sakai and colleagues synthesized a series of pentacyclic analogs of natural lamellarins through Bischler–Napieralski condensation [39]. An in vitro evaluation of the anticancer potency of novel benzo[6,7]indolo[3,4-*c*]isoquinolines was carried out using a panel of human cancer cell lines (breast, CNS, colon, lung, melanoma, ovarian, renal, stomach, and prostate). In the testing, *N*-(3-morpholinopropyl)-substituted isoquinoline derivative **3** (Figure 8) appeared to be the most effective antiproliferative compound, with a mean GI_50_ value of 39 nM. Compound **3** showed clear bands of nicked DNA, with intensities similar to the reference topoisomerase inhibitors—Camptothecin (CPT) and lamellarin D. As a result, 2-methoxy-13-(3-morpholinopropyl)-13*H*-benzo[6,7]indolo[3,2-*c*]isoquinoline-3,10-diol (**3**) inhibits the activity of topoisomerase I by stabilizing the enzyme–DNA complex. The authors suggest that nitrogen substitution on the pyrrole nitrogen atom plays a significant role in the antiproliferative properties of these isoquinoline derivatives. Their activity can be improved by further optimizing the *N*-alkyl side chain. Consequently, *N*-substituted derivatives could prove to be promising scaffolds for anticancer drug development.

**Figure 9:**
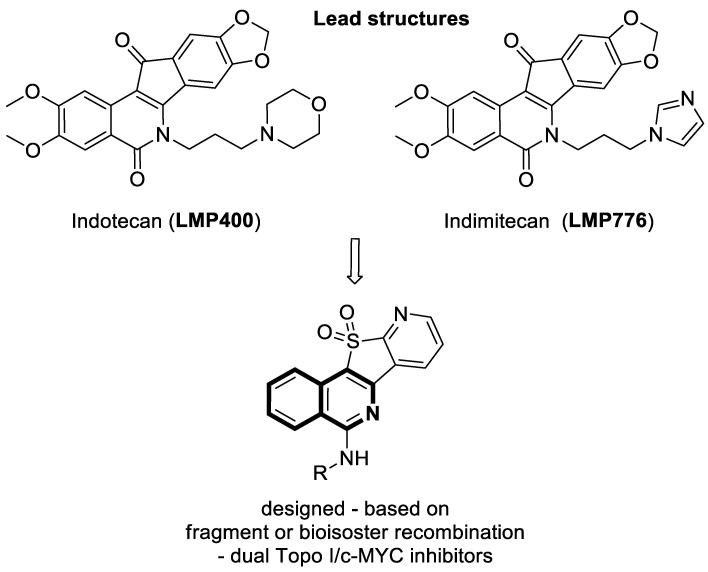
Type I topoisomerase inhibitors—Indotecan (**LMP400**) and Indimitecan (**LMP776**)—as lead structures for novel Topo I/c-MYC dual inhibitors.

As compared with the subtypes IA and IB topoisomerases, type IA topoisomerases change the linking number of circular DNA strands by strict units of 1, while type IB topoisomerases change the linking number by multiples of 1. Among the topoisomerase I inhibitors under clinical trials designed to treat advanced solid tumors are indenoisoquinoline derivatives—Indotecan (**LMP400**) and Indimitecan (**LMP776**) (Figure 9).

**Figure 10:**
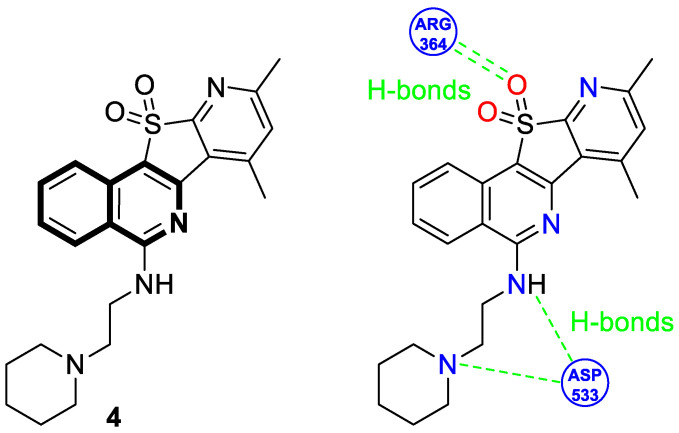
Topo I inhibitor **4** and results of molecular docking of **4** with Topo I/DNA.

In 2025, based on the structure of the 7-azaindenoisoquinoline moiety and quindoline scaffold, Zheng et al. evaluated the Topo I inhibitory and antiproliferative activity of designed pyrido[3′,2′:4,5]thieno[3,2-*c*]isoquinoline 11,11-dioxides against frequent human cancers, including non-small-cell lung (NCI-H460 and A549), renal (ACHN), oral (Cal27), and gastric (MGC-803) carcinomas, as well as non-cancerous human urinary tract epithelial cells (SV-HUC-1) [40]. The most potent Topo I inhibitor **4** (Figure 10) displayed antiproliferative activity against a Cal27 cell line, with a calculated IC_50_ value of 1.12 μM. This oral cancer cell line is characterized by a high Topo I expression level.

Moreover, it was discovered that compound **4** repressed transcription factor c-MYC, as well as exerting its potent inhibitory activity against the PI3K/AKT/NF-κB pathway. According to further studies, 7,9-dimethyl-5-((2-(piperidin-1-yl)ethyl)amino)pyrido[3′,2′:4,5]thieno[3,2-*c*]isoquinoline 11,11-dioxide (**4**) was also shown to induce S-phase cell-cycle arrest, inhibit colony formation and migration, and trigger both autophagy and apoptosis in the representative Cal27 cell line. The estimated number of apoptotic cells in the early and late phases increased from 5.9% to 32.8%. Additionally, in vivo studies revealed that compound **4** inhibited the growth of a human oral cancer Cal27 xenograft tumor without significant adverse effects on the major organs of animals. After 21 days of treatment at a dose of 20 mg/kg, the relative tumor proliferation and suppression rates were approximately 34% and 65%, respectively. To elucidate the mechanism of Topo I inhibition, molecular docking was used to predict compound **4**–enzyme binding modes (Figure 10). It was found that compound **4** could interact with enzymes through two hydrogen bonds formed between the sulfonyl group and one arginine residue (ARG-364), with distances of 2.2 Å and 2.5 Å, respectively. It may suggest the importance of the sulfonyl group for Topo I inhibition. Also, the 2-(piperidin-1-yl)ethanamine side chain of the docked molecule **4** interacted with asparagine (ASP-533), resulting in the formation of two additional hydrogen bonds with distances of 2.0 Å and 3.4 Å. In addition, the polyaromatic ring was capable of embedding into double-stranded DNA.

**Figure 11:**
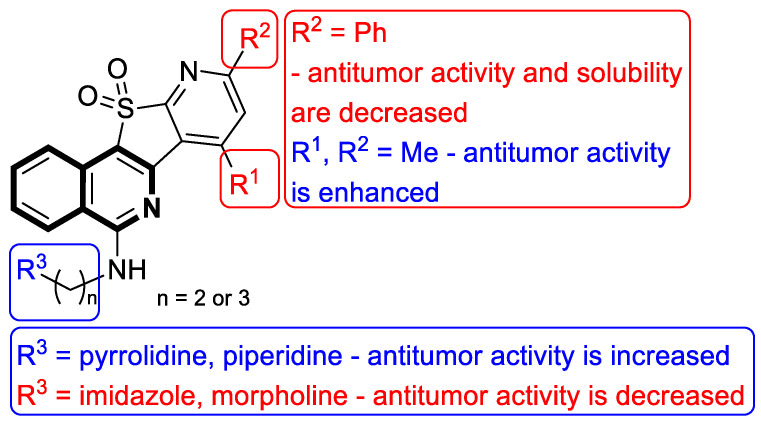
SAR analysis for pyrido[3′,2′:4,5]thieno[3,2-*c*]isoquinoline 11,11-dioxide derivatives designed by Zheng.

Based on an SAR analysis (Figure 11), it was found that the antitumor potency of the designed pyrido[3′,2′:4,5]thieno[3,2-*c*]isoquinoline 11,11-dioxide derivatives was significantly improved when two methyl groups were introduced in the polyaromatic ring (R^1^ and R^2^). Activity was also affected by the length of the side chain (R^3^), where three carbon atoms seemed to be optimal. On the other hand, compounds with piperidinyl or pyrrolidinyl scaffolds at the end of the chain mostly displayed potent antitumor and Topo I inhibitory activity, while those with morpholinyl and imidazolyl exerted a low cytotoxic effect. This suggests that the nature of the heterocyclic ring at the end of the chain may affect antitumor activity. The presence of a heterocyclic ring with more basic properties, such as pyrrolidine or piperidine, appears to be more favorable for the anticancer activity of this class of compounds. Based on these findings, compound **4** can be identified as a candidate for further development of Topo I/c-MYC dual inhibitors that combat oral cancers.

#### 2.1.2. Dihydroisoquinoline-Based Compounds

**Figure 12:**
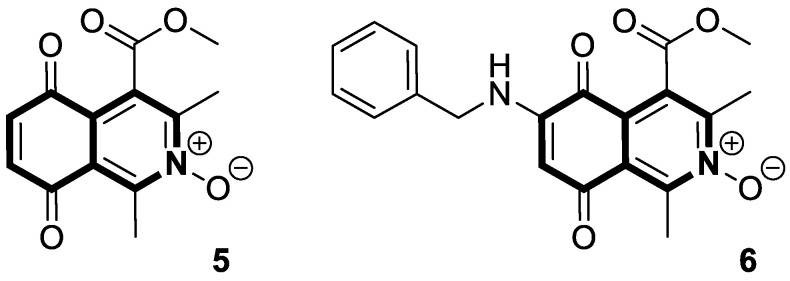
Dihydroisoquinoline *N*-oxide **5** and its amino-benzylated analog **6**, with cytotoxic properties.

Apart from the extensively explored isoquinolines, Kruschel et al. designed novel anticancer derivatives containing a privileged isoquinoline-5,8-dione pharmacophore, known as isoquinolinequinone (IQQ). This unique framework was reported in the structure of Caulibugulones—marine cytotoxic —and Mansouramycins—found as secondary active metabolites of *Streptomyces* [41]. It was found that the transformation of the IQQ framework into *N*-oxide resulted in an alteration of the electronic properties of the quinone to improve its overall cytotoxicity. Preliminary studies on the precursor scaffold—4-(methoxycarbonyl)-1,3-dimethyl-5,8-dioxo-5,8-dihydroisoquinoline *N*-oxide (**5**) (Figure 12) at 10 µM—revealed notable cytotoxic activities against several human cancer lines, including melanoma, breast, colon, and ovarian, with a mean cell growth of −29.45%. A regioselective substitution of the IQQ framework led to an amino-benzylated analog **6** (Figure 12), which exhibited a lower mean cell growth of −35.1%. A five-dose screening against the full panel of 53 common human tumor cells showed excellent potency (the mean GI_50_ value was 0.91 µM). The most responsive to 6-(benzylamino)-4-(methoxycarbonyl)-1,3-dimethyl-5,8-dioxo-5,8-dihydroisoquinoline *N*-oxide (**6**) were breast cancer cell lines MDA-MB-468 (GI_50_ = 259 nM), MCF-7 (GI_50_ = 290 nM), melanomas MALME-3M (GI_50_ = 134 nM) and MDA-MB-435 (GI_50_ = 208 nM), and ovarian adenocarcinoma OVCAR-3 (GI_50_ = 272 nM), including a high-grade serous ovarian cancer cell line (HGSOC) OVCAR-8 (GI_50_ = 484 nM) that has a low survival rate.

**Figure 13:**
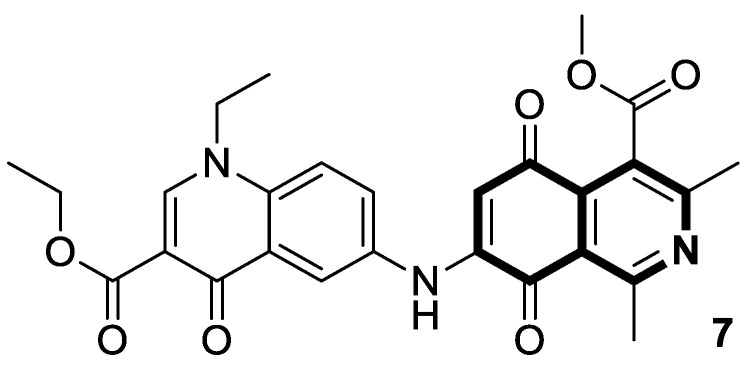
A novel isoquinoline-based AMPK activator—ethyl 1-ethyl-6-((4-(methoxycarbonyl)-1,3-dimethyl-5,8-dioxo-5,8-dihydroisoquinolin-7-yl)amino)-4-oxo-1,4-dihydroquinoline-3-carboxylate (**7**).

One of the most crucial enzymes involved in regulating the growth, metabolism, and survival of cells is AMP-activated protein kinase (AMPK). As well as regulating energy homeostasis, AMPK also inhibits the unfolded protein response (UPR), as it inhibits the synthesis of protein and the growth of cells. Since the enzyme regulates key metabolic pathways such as glucose and lipid homeostasis, it may reprogram eukaryotic cell metabolism, making it a promising therapeutic target for metabolic diseases or cancers. Nascimento Mello and co-workers, based on the structure of a cytotoxic isoquinolinequinone from marine *Streptomyces*—mansouramycin—identified a novel isoquinoline-based AMPK activator that inhibits mTORC1 signaling (compound **7**, Figure 13) [42].

In a breast cancer cell line (MCF-7), compound **7** was shown to induce the dose-dependent phosphorylation of AMPK and its downstream target acetyl-CoA carboxylase (ACC). Additionally, compound **7** has the ability to decrease lactate formation and glucose uptake, leading to a significant decreasing intracellular ATP content. As a result, AMPK activation by compound **7** inhibited the activity of the mammalian target of Rapamycin complex 1 (mTORC1). Treatment with **7** also triggered both apoptosis and necrosis in MCF-7 cells. Moreover, the isoquinoline derivative **7** stimulates autophagy in these cells, increasing it by 1.6-fold at 12 µM and by 8-fold at 24 µM.

Pancreatic cancer is one of the most aggressive and deadly malignancies, with survival rates below 10% after five years. This tumor has a high propensity for metastasis, with cancerous cells migrating to particular critical organs, such as the liver, duodenum, or stomach. Unfortunately, most cancer patients are diagnosed at an advanced metastasis stage [43,44,45].

Antimetabolites that interfere with DNA synthesis, such as Gemcitabine, are used for treating advanced and metastatic stages of pancreatic cancer. Although Gemcitabine inhibits rapid cell division in tumor areas with blood vessels, it is ineffective when nutrients and oxygen are low. In prolonged periods of nutrient deprivation, pancreatic cancer cells are capable of surviving [46,47,48].

**Figure 14:**
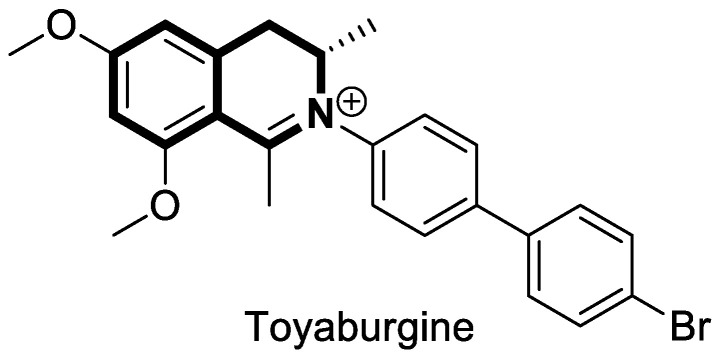
Antimetastatic compound—Toyaburgine—against human pancreatic cancer.

The isoquinoline moiety is an important part of the structure of interesting structurally diverse biaryl alkaloids found in Asian and African plants—naphthylisoquinolines (NIQs)—since they display strong cytotoxic activity and create the phenomenon of an axial chirality due to the restricted rotation around a *C*,*C*- or *N*,*C*-bond biaryl axis (they are rotationally hindered) [49,50]. Based on the structure of these *N*,*C*-coupled naphthylisoquinoline alkaloids, an *N*-biphenyl-dihydroisoquinoline derivative—Toyaburgine (Figure 14)—was synthesized by a team led by Brigmann. This simplified structural analog was designed to avoid axial chirality by introducing an *N*-biphenyl substitutent that can freely rotate [51].

The compound has shown a strong antimetastatic nanomolar potential against the human pancreatic cancer cell line (MIA PaCa-2) (PC_50_ = 3.8 nM). The fact that pancreatic cancer cells migrate to different organs leads to invasion and colony formation, making surgical interventions ineffective. Therefore, the inhibitory effect on MIA PaCa-2 colony formation of Toyaburgine was assessed. It was found that the derivative, at a concentration of 5 μM, strongly inhibited cell colony formation in a concentration-dependent manner, indicating the effective antimetastatic potential of the compound. Furthermore, Toyaburgine inhibits the PI3K/Akt/mTOR signaling pathway, which is involved in progression, angiogenesis, and metastasis. It is well known that the PI3K/Akt/mTOR pathway contributes to Gemcitabine resistance in pancreatic cancer, and the dysregulation of this pathway is frequently observed in pancreatic cancer. This makes targeting the PI3K/Akt/mTOR pathway the therapeutic strategy for overcoming drug resistance. A Western blot analysis clearly showed that MIA PaCa-2 cells treated with the compound downregulated key proteins in a concentration-dependent manner, such as phosphorylated Akt (p-Akt S473), PI3K (p110α and p110β), Akt, mTOR, and phosphorylated mTOR (p-mTOR S2448). Additionally, an in vivo assessment proved that Toyaburgine has significant anticancer properties. The tested compound inhibited tumor growth dose-dependently, with high-dose treatments being more effective than low-dose treatments. Combined with Gemcitabine, the compound showed a synergistic effect, significantly reducing tumor weight and volume. Considering the superior performance of combination therapy, the authors suggest that Toyaburgine may potentiate Gemcitabine activity, potentially overcoming drug resistance and reducing adverse effects.

**Figure 15:**
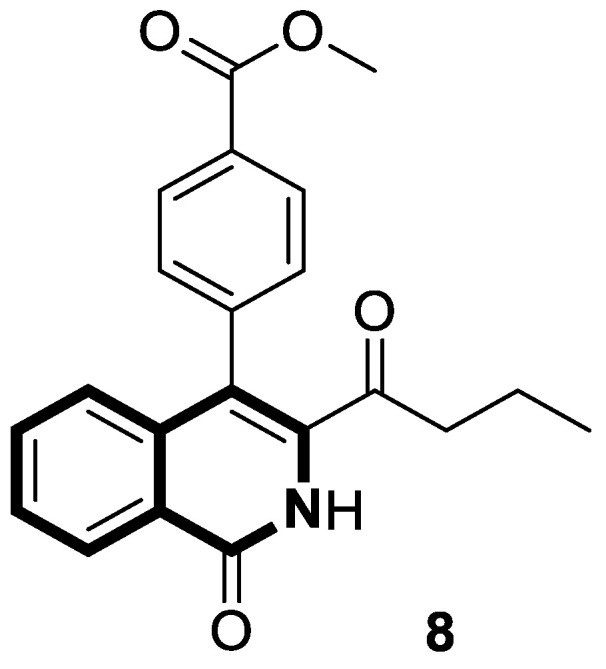
A novel 3-acyl isoquinolin-1(2*H*)-one derivative **8**, which induces G2-phase arrest, apoptosis, and GSDME-dependent pyroptosis in breast cancer.

Clinically, breast cancer has particularly high recurrence, metastasis, and mortality rates. This complex disease is classified into distinct subtypes, which affect prognosis and treatment options. The acquired resistance of breast cancer cells, limited effectiveness of several drugs, and their significant toxicity stimulate the search for novel synthetic agents with high potency and tumor selectivity. The efficient synthesis of 3-acyl isoquinolin-1(2*H*)-one derivatives and their anticancer screening prompted Ma and colleagues to the deep examination of the effects of those identified during preliminary tests, e.g., methyl 4-(3-butyryl-1-oxo-1,2-dihydroisoquinolin-4-yl)benzoate (**8**) (Figure 15), on breast cancers MCF-7 and MDA-MB-231 [52].

Compound **8** in both cancer cell lines showed a significantly better antiproliferative activity than the reference drug—5-Fluorouracil—in a dose-dependent manner, with calculated half-maximum inhibitory concentrations ranging from 2.4 μM to 5.7 μM. Upon cytotoxicity testing against normal human mammary epithelial cells (MCF10A), compound **8** proved to be selectively toxic towards cancer cells. As a result of treating MCF-7 and MDA-MB-231 cells with compound **8**, a dose-dependent increase in the proportion of cells in the G2 phase was observed. Furthermore, a Western blot analysis clearly confirmed that compound **8** induced G2-phase cell-cycle arrest. A decrease in CDK1 expression was also observed. It was demonstrated that compound **8** is involved in the mitochondrial-mediated intrinsic apoptosis pathway. The pro-apoptotic protein Bax expression was significantly increased by compound **8**, while Bcl-2 expression was significantly reduced. The results of further studies revealed that the tested compound **8** blocks the mitogen-activated protein kinase MAPK/ERK or Ras-Raf-MEK-ERK and p38 MAPK pathways, which play an important role in signal transduction in cells. Finally, an induction of programmed cell death via pyroptosis by **8** was observed, which involves the caspase-3 cleavage of the protein gasdermin E (GSDME). An *N*-terminal cleavage fragment of GSDME has a pore-forming ability. In this way, caspase-3-mediated pyroptosis may contribute a significant role in cancer treatment. It was found that the GSDME cleavage was enhanced by **8** in a dose-dependent manner.

**Figure 16:**
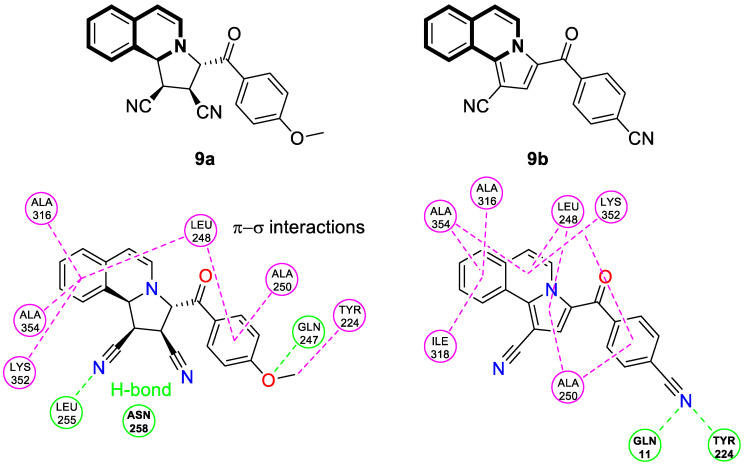
Pyrrolo[2,1-*a*]isoquinoline derivatives **9a** and **9b**, designed by Al-Matarneh, with anticancer activity and molecular docking results obtained for 3-(4-methoxybenzoyl)-1,2,3,10b-tetrahydropyrrolo[2,1-*a*]isoquinoline-1,2-dicarbonitrile (**9a**) and 3-(4-cyanobenzoyl)pyrrolo[2,1-*a*]isoquinoline-1-carbonitrile (**9b**).

In 2021, interesting cyano-substituted pyrrole-fused isoquinolines were reported by Al-Matarneh and colleagues [53]. In this study, a (tetrahydro)pyrrolo[2,1-*a*]isoquinoline scaffold was constructed via the [3+2] cycloaddition reaction of cycloimmonium ylides with fumaronitrile. The identified cycloaddition products possessed two different configurations of the nitrile groups—identical to the parent alkene or reverse. The synthesized compounds were evaluated for anticancer potency against a panel of human cancer cell lines (NCI). Most of the tested derivatives were inactive, except for isoquinoline with reverse configuration **9a** (Figure 16), which selectively inhibited ovarian cancer cells (OVCAR-8, GI = 100%, 10^−5^ M) and colon cancer cells (SW-620, GI = 82%, 10^−5^ M). Moreover, a non-selective inhibition and moderate activity against several cancer lines were proven for compound **9b** (Figure 16).

To gain more insight into the possible mechanisms of anticancer activity of compounds **9a** and **9b**, molecular docking simulations were conducted for the α,β-tubulin Colchicine binding site (Figure 16). Compound **9a**, with a binding energy value of −9.7 kcal/mol after being placed in the binding pocket, was stabilized through hydrophobic contact with amino acids in the tubulin β subunit. In the case of 3-(4-cyanobenzoyl)pyrrolo[2,1-*a*]isoquinoline-1-carbonitrile (**9b**), with a Gibbs free energy value ∆G = −10.8 kcal/mol, an additional interaction through a nitrile group with tyrosine 224 and glutamine 11 of the α-subunit was observed.

Neuroblastoma is one of the most common types of pediatric cancer, accounting for nearly 15% of deaths from newborn tumors. While rigorous treatment, including radio-, chemo-, and immunotherapy, surgery, and stem cell transplantation, is available for children at high risk, their survival rates are poor [54,55,56,57].

**Figure 17:**
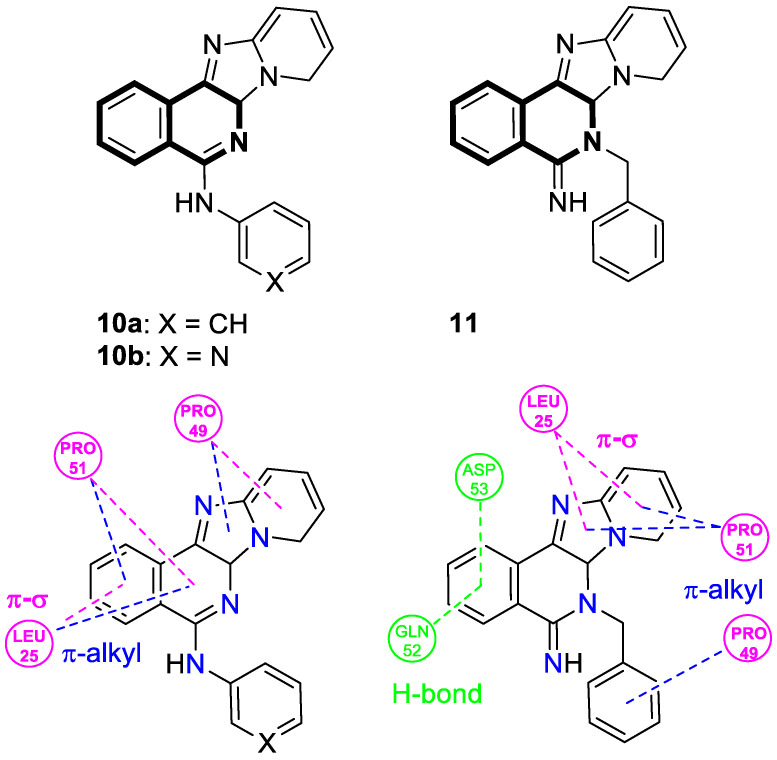
Ellipticine analogs **10a**, **10b**, and **11**—potential anti-neuroblastoma chemotherapeutic agents—and interactions between pro-apoptotic Bax protein and compounds **10a**, **10b**, and **11**.

In 2021, Tber and colleagues reported on the identification of isosteres of an isoquinoline bearing a tetracyclic alkaloid, Ellipticine, via a one-pot, three-component Groebke–Blackburn–Bienaymé (GBB) reaction that involved a 2-aminoheteroaryl derivative, 2-formylbenzonitrile, and tert-butylisocyanide in DCM at room temperature [58]. The anticancer potential of the synthesized pyrido[2′,1′:2,3]imidazo[4,5-*c*]isoquinolin-5-amine derivatives were evaluated in the human neuroblastoma cell line using a colorimetric cell viability assay. Even though the tested isoquinoline derivatives showed a moderate antiproliferative activity against cancer cells, *N*-phenyl-6a,8-dihydropyrido[2′,1′:2,3]imidazo[4,5-*c*]isoquinolin-5-amine (**10a**), *N*-(pyridin-3-yl)-6a,8-dihydropyrido[2′,1′:2,3]imidazo[4,5-*c*]isoquinolin-5-amine (**10b**), and 6-benzyl-6,6a-dihydropyrido[2′,1′:2,3]imidazo[4,5-*c*]isoquinolin-5(8*H*)-imine (**11**) (Figure 17) had a regulatory effect on apoptosis via mitochondrial dysfunction.

When compounds **10a**, **10b**, and **11** were exposed at a concentration of 10 μM, the number of human neuroblastoma cell colonies decreased approximately by 51%, 38%, and 27%, respectively. A significant alteration in caspase-3 and PARP-1 cleavage was also observed when these compounds were applied to neuroblastoma cells. The results from the protein expression analysis revealed that, during the treatment of the neuroblastoma cell line SH-SY5Y with the tested compounds, apoptotic proteins such as Bax, cleaved caspase-3, and cleaved PARP-1 were upregulated. Moreover, it was found that an increased Bax protein level in cells at the highest concentrations of compounds was accompanied by a concurrent decrease in the bcl-2 protein level. Since the balance between Bax and bcl-2 controls cellular apoptosis, this clearly indicates that the tested compounds have a regulatory potential for apoptosis. The identified Ellipticine analogs **10a**, **10b**, and **11** enhance caspase-3 cleavage in a dose-dependent manner, initiating the intrinsic apoptotic cascade and inducing mitochondrial dysfunction. Furthermore, the promising pro-apoptotic properties of the compounds were supported by molecular modeling investigations. The in silico results indicated the possible binding modes to the pro-apoptotic Bax protein retrieved from the RCSB protein data bank. In docking studies of the ligands **10a**, **10b**, and **11**, different affinities were observed for their interaction with the helices α1 and α2. As shown in Figure 17, π-σ, π-alkyl, and π-donor H-bond mixed interactions, as well as non-classical hydrogen bonds (ligand **11**), seem to play a significant role in activating the Bax protein. All three docked ligands **10a**, **10b**, and **11** interacted primarily with leucine 25 and the two proline residues 49 and 51.

#### 2.1.3. Tetrahydroisoquinoline-Based Compounds

Since the mid-1950s in the 20th century, Noscapine—a tetrahydroisoquinoline alkaloid isolated from *Papaver somniferum*—has been frequently used as a cough suppressant. There was great interest in Noscapine after the anti-mitotic properties of the compound were discovered. Its low toxicity and high bioactivity make Noscapine an ideal platform for subsequent modifications and advancement in the pursuit of innovative chemotherapeutic agents [59]. To date, several novel semi-synthetic Noscapine derivatives have been developed, and their biological activities have been extensively studied as potential pharmaceuticals.

**Figure 18:**
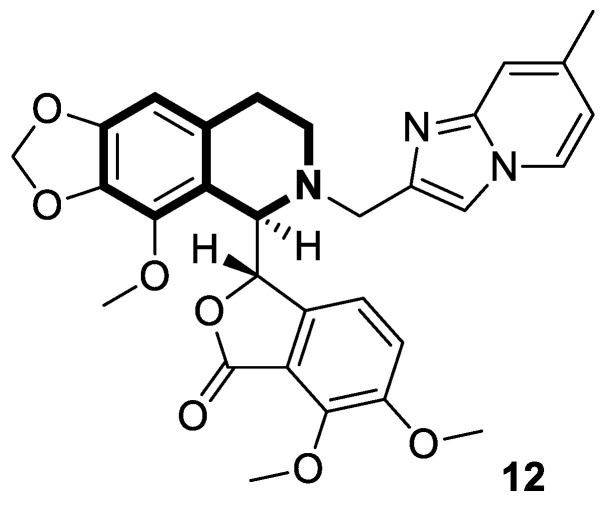
*N*-imidazopyridine derivative of Noscapine **12** as potent anti-tubulin agent.

Dash and colleagues described a novel class of Noscapine derivatives tethered to the imidazo[1,2-*a*]pyridine (impy) core, which exhibited potent cytotoxicity against breast cancer cell lines (MCF-7 and MDA-MB-231), with IC_50_ values ranging from 3.7 µM to 32.4 µM [60]. These Noscapine hybrids display no toxicity against normal human embryonic kidney (HEK) cells (IC_50_ value > 1500 µM). The tested compounds showed a high affinity for tubulin binding, with equilibrium dissociation constants ranging from 35 µM to 78 µM. Moreover, docking studies at the noscapinoid site of tubulin using the GlideXP score function revealed better scores (−6.213 to −7.897 kcal/mol) than were achieved for Noscapine (−4.96 kcal/mol). When the MDA-MD-231 line was treated with the most active compound **12** (Figure 18) at an IC_50_ concentration of 5.6 µM, a significant number of cells in the early (45%) and late phase of apoptosis (35%) were observed after 72 h of incubation. The study revealed that *N*-imidazopyridine derivative **12** arrested the cell cycle in the G2/M phase. According to in vivo xenograft mouse model studies, compound **12** substantially reduced the volume of the implanted MCF-7 tumor without adversely affecting vital organs.

Of the various classes of naturally occurring isoquinoline-based alkaloids, special attention has been paid to the bis-benzylisoquinoline-based compound tetrandrine (TET) [61,62,63,64,65,66,67,68]. Recent studies revealed that this alkaloid extracted from *Stephania tetrandra* displays tumorigenesis suppressing properties via multiple signaling pathways involving the VEGF/HIF-1α/ICAM-1 or the PI3K/AKT/ mTOR [69,70].

**Figure 19:**
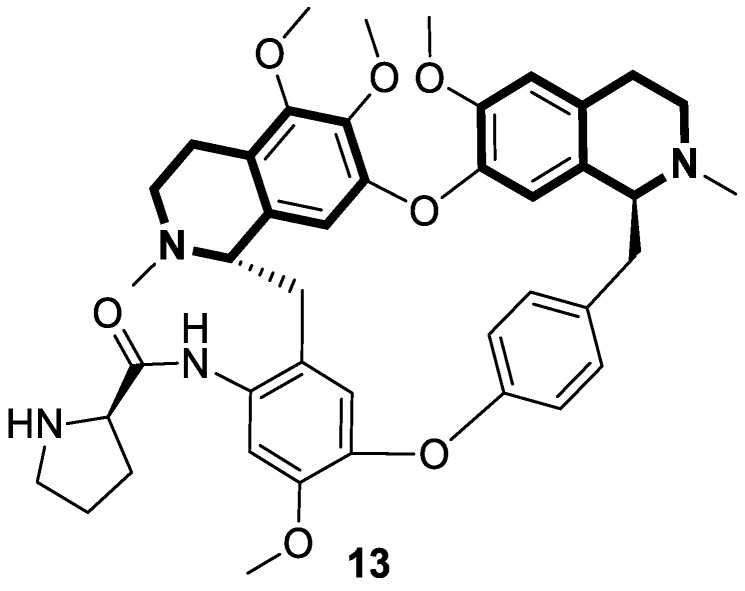
14-*N*-amino acid-substituted TET derivative **13** with anticancer potency.

Considering the preeminent antitumor properties of tetrandrine (TET), in 2022, Wang and co-workers designed a series of 14-*N*-amino acid-substituted TET derivatives with improved anticancer potency and aqueous solubility [71]. Among the synthesized amino acid derivatives, compound **13**, bearing proline, shown in Figure 19, exerted the highest antiproliferative activity against the human colorectal cancer cell line (HCT-15), with an IC_50_ value of 0.57 μM. Importantly, in comparison to tetrandrine, the solubility of TET derivative **13** was enhanced by 5 times. As determined through a Western blot analysis, proline TET derivative **13** in the HCT-15 line induced autophagy—significantly decreasing p62/SQSTM1 (sequestosome 1) levels and enhancing Beclin-1 levels and LC3-II/LC3-I ratios. As a further result of compound **13**, double-staining and flow cytometry experiments showed that compound **13** impairs the morphology and motility of colorectal cancer cells, inducing their death. Moreover, the anti-angiogenic effect of **13** was demonstrated—the compound effectively inhibited the proliferation and migration of Human Umbilical Vein Endothelial Cells (HUVEC). Based on these findings, amino acid TET derivative **13** appears to have the potential to be a promising candidate for further testing.

**Figure 20:**
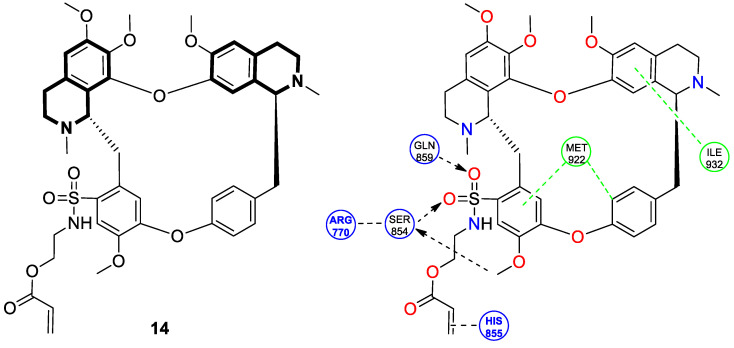
Sulfonamido-TET ethyl acrylate **14** exhibiting anticancer activity and binding capacity of compound **14** to PI3K.

Even though TET has antitumor activity, it still faces some challenges in its medical applications, including poor bioavailability, and possibly unknown targets [72]. From this point of view, in 2024, Ling and colleagues performed some rational structural modifications to design TET-based sulfonyl compounds, which could be particularly valuable as highly active and target-oriented anticancer lead compounds [73]. It was found that sulfonamido-TET ethyl acrylate **14** (Figure 20) exerted an in vitro high potency against the human colorectal carcinoma cell line (HCT116) and BALB/c mouse-derived colorectal carcinoma cell line (CT26) Compound **14** effectively inhibits the proliferation and migration of colorectal carcinoma cells.

The results of a cytotoxicity test revealed that compound **14** inhibited colon cancer cell viability (HCT116 and CT26) in a dose- and time-dependent manner, with IC_50_ values of 0.48 μM and 0.58 μM after 24 h and 0.23 μM and 0.30 μM after 48 h, respectively. Morphological changes in colon cancer cells after 24 h of exposition with compound **14** revealed a declining number of cells with increased concentration and time. Higher concentrations of compound **14** resulted in wrinkles and the detachment of cells, indicating that compound **14** effectively promotes apoptosis. It was observed that compound **14** triggered cell cycle arrest—the number of cancer cells in the S-phase was low, while, in the G2/M phase, it increased. In HCT116 cells at a concentration of 0.4 μM, the percentage of cells blocked in the G2/M phase increased from 16% to 37%. Similarly, the proportion of CT26 cells arrested in the G2/M phase increased from 12% to 32%. The suppression of the PI3K/AKT/mTOR signaling pathway activates mitochondrial damage and the mitochondrial apoptotic pathway.

Molecular docking simulations indicated that compound **14** forms a specific interaction with the binding pocket of phosphoinositide 3-kinase (PI3K) by participating in two distinct hydrogen-π stackings with isoleucine (Ile932) and methionine (Met922) (Figure 20). As a result of this multisite interaction, the possible rigidity may be enhanced, along with the binding affinity for PI3K. Additionally, strong hydrogen bonds were formed between the sulfonyl group and both arginine (Arg770) and glutamine (Gln859) residues. Moreover, the potential interaction between the terminal double bond and histidine (His855) may enhance the stability of the compound–protein complex.

**Figure 21:**
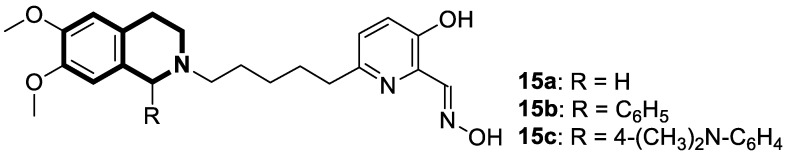
THIQ-oxime hybrids **15a**, **15b**, and **15c** with anticancer activity.

Recent studies demonstrated that certain 1,2,3,4-tetrahydroisoquinolines (THIQ) with an oxime moiety display diverse biological activities [74,75], including the strong ability to induce cytotoxicity and apoptosis in various cells [76]. In this line, Zandona and colleagues selected three THIQ-oxime hybrids, **15a**, **15b**, and **15c** (Figure 21), to evaluate their impact on common malignant human tumors: breast MDA-MB-231 and MCF-7 (ER-positive cells, progesterone receptor positive, and HER2 negative), prostate cancer PC-3, and malignant glioblastoma U251 [77].

After 24 h of exposure, compounds **15a**, **15b**, and **15c** exhibited time- and concentration-dependent cytotoxicity to ER-positive breast cancer cells (MCF-7) with calculated IC_50_ values of 74 µM, 21 µM, and 7 µM. The THIQ derivative substituted with a phenyl ring—6-(5-(6,7-dimethoxy-1-phenyl-3,4-dihydroisoquinolin-2(1*H*)-yl)pentyl)-3-hydroxypicolinaldehyde oxime (**15b**)—showed cytotoxicity against triple-negative breast cancer cells MDA-MB-231 (IC_50_ = 22 µM), whereas the compound bearing *N*,*N*-dimethylaminophenyl substituent **15c** displayed a cytotoxic effect against malignant glioblastoma multiforme cells U251 (IC_50_ = 36 µM). The reference antineoplastic agent Temozolomide achieved an IC_50_ value of 176.5 µM. Moreover, compound **15c** displayed activity against the MDA-MB-231 cell line, being 75 times less potent than Doxorubicin (IC_50_ = 21 µM vs. IC_50_ = 0.28 μM). It should be noted that the non-substituted tetrahydroisoquinoline derivative (**15a**, R = H) showed a cytotoxic effect only against the MCF-7 cell line (IC_50_ = 74 µM).

In MCF-7 cells, all tested compounds decreased mitochondrial membrane potential, with statistical significance shown for compound **15c**. A major effect of the tested compounds was the activation of caspase-9 and the modulation of the mitochondrial membrane potential, which implicates mitochondrial activation of the intrinsic apoptosis signaling pathway.

**Figure 22:**
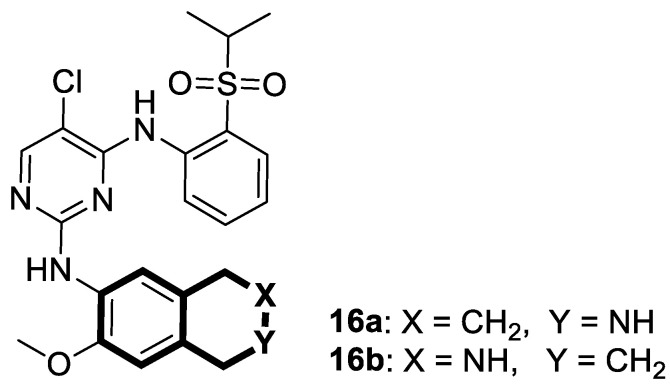
Designed THIQs **16a** and **16b** as highly potent ALK inhibitors.

The insulin receptor tyrosine kinase (RTK) family includes the anaplastic lymphoma kinase (ALK). The ALK gene has gained much attention due to the association between ALK-positive tumors that may be found in various types of cancer including diffuse large B-cell lymphoma (DLBCL), non-small-cell lung cancer (NSCLC), and anaplastic large-cell lymphoma (ALCL). The first ALK inhibitor drug to be approved for the treatment of ALK-positive NSCLC was Crizotinib [78,79]. It has only taken one to two years since Crizotinib was introduced into the clinic for patients to develop Crizotinib-resistant mutants. A potent alternative to Crizotinib and to anaplastic lymphoma kinase is second-generation Ceritinib (**LDK378**), which can overcome drug-resistant issues [80]. According to some researchers, the toxicity profile of **LDK378** may originate from the 1,4-diaminophenyl scaffold of the pyrimidine ring. A significant effort was made to circumvent this problem by introducing a more metabolically favorable piperidinyl substituent. As a result of exploring the two-position side chains of the pyrimidine in **LDK378** with a tetrahydroisoquinoline (THIQ) moiety, novel highly effective ALK inhibitors **16a** and **16b** were identified [81] (Figure 22).

THIQs **16a** and **16b** demonstrated in vitro and in vivo xenograft model inhibitory efficacies comparable to **LDK378** against various ALK mutant enzymes, including G1202R. Based on these findings, it is apparent that these compounds can be further optimized as potential ALK inhibitors that can overcome the resistance issues associated with Crizotinib and **LDK378**. On the other hand, isoquinoline-based derivatives appear completely inactive, whereas dihydroisoquinolines only have a slight active level in enzyme assays. Based on the docking results, the terminal secondary amine on the THIQ scaffold may significantly affect ALK binding in these compounds.

Microtubules (*microtubuli cellulares*) are polymerized tubular structures produced by α- and β-tubulin dimers that constitute a key element of the cytoskeleton. Among many cellular functions, tubulin promotes cytoskeletal components’ integrity, regulates cell signaling, and segregates chromosomes during mitosis. Many natural and synthetic compounds may target microtubules by binding to different protein domains that prevent their polymerization or depolymerization. Therefore, tubulin has emerged as a potential molecular target for anticancer drugs.

**Figure 23:**
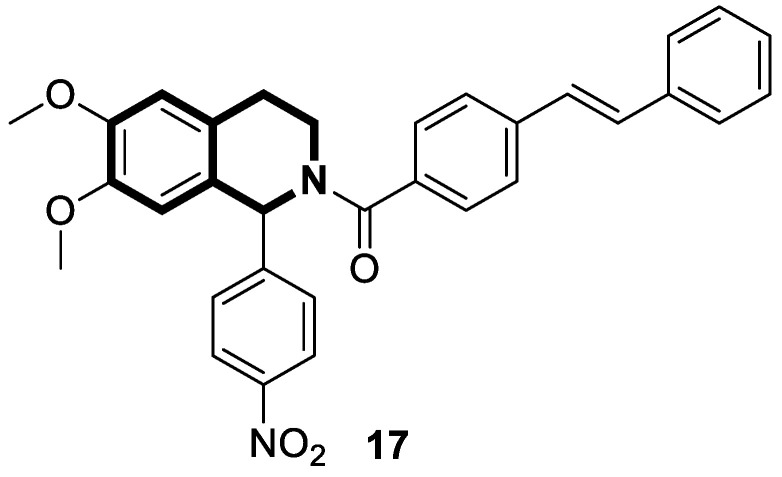
Tetrahydroisoquinoline stilbene derivative **17** as potential antitumor agent.

Li and co-workers designed a novel class of tetrahydroisoquinoline-stilbene derivatives that display in vitro inhibitory activity against human lung cancer cells A549, breast cancer cells MCF-7, and colorectal carcinoma HT-29 [82]. An exceptional level of cytotoxicity was demonstrated for the 6,7-dimethoxy-1-(4-nitrophenyl)-3,4-dihydroisoquinolin-2(1*H*)-yl)(4-styrylphenyl)methanone (**17**) (Figure 23), with an IC_50_ value of 25 nM. As assessed through a tubulin polymerization assay, compound **17** showed a higher inhibitory activity than the reference Colchicine at the same concentration. In a flow cytometry and Western blot study, **17** reduced the mitochondrial membrane potential, produced ROS, increased the level of cleaved caspase-3, and promoted the release of cytochrome C from mitochondria into the cytoplasm. These results indicated that tetrahydroisoquinoline stilbene derivative **17** caused apoptosis via the mitochondrial-dependent apoptotic pathway. According to the findings, compound **17** arrested A549 cells in the G2/M phase by downregulating the expression of cell division cycle 2 (Cdc2) and upregulating the expression of proliferating cell nuclear antigen (PCNA) and cyclin B1.

An evolutionarily conserved multifunctional transcription factor known as nuclear factor-κB (NF-κB) is a key mediator that regulates a wide range of physiological processes, such as cell proliferation and differentiation, inflammatory and immune responses, and apoptosis. Therefore, through the abnormal modulation of the NF-κB signaling cascade, it may be involved in multiple human diseases, chronic inflammation, autoimmune diseases, neurodegeneration, and tumors. In several cancers, such as the colon, breast, and melanoma, NF-κB is upregulated and suppresses apoptosis, inducing proliferation and promoting angiogenesis and metastasis.

**Figure 24:**
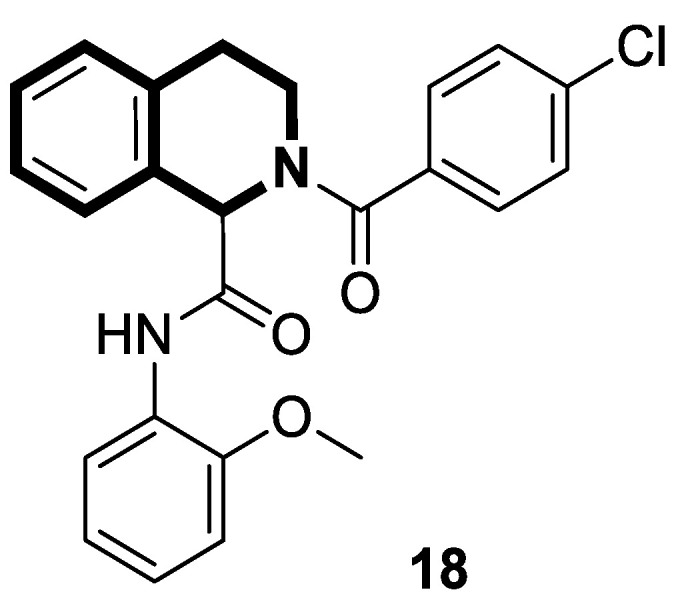
1,2,3,4-tetrahydroisoquinoline-1-carboxamide **18** with antiproliferative activity, designed by Sim et al.

Sim and collaborators present novel 1,2,3,4-tetrahydroisoquinoline derivatives that display anticancer activities against various human cancer cell lines. Cancer treatment may be more effective in inhibiting the NF- κB signaling pathway, and these compounds were rationally designed based on known NF-κB inhibitors [83]. Based on preliminary SAR results, it was found that the isoquinoline moiety appeared to be less effective for the anticancer potency of the designed compounds. The selective hydrogenation of the isoquinoline ring into the 1,2,3,4-tetrahydroisoquinoline core and the presence of the 4-chlorobenzoyl group provided a beneficial anticancer activity. As revealed through in vitro studies, the 2-(4-chlorobenzoyl)-*N*-(2-methoxyphenyl)-1,2,3,4-tetrahydroisoquinoline-1-carboxamide (**18**) (Figure 24) with a methoxyl group exhibited the most antiproliferative activity in an SRB cytotoxicity assay against six human cancer cell lines, including NUGC-3 (gastric), ACHN (renal), NCl-H23 (lung), HCT15 (colon), MDA-MB- 231 (breast), and PC-3 (prostate), with GI_50_ values ranging from 1.591 to 2.281 μM. The performed immunocytochemical analysis has demonstrated that compound **18** inhibits the nuclear translocation of NF-κB in LPS-stimulated MDA-MB-231 cells.

In the current state of chemotherapy, multidrug resistance (MDR) is one of the most significant threats. There are different mechanisms contributing to MDR—two of the most critical ones are ABC transporter family proteins—the P-glycoprotein (P-gp or ABCB1) that significantly enhances drug efflux from cancerous cells and the metabolic cytochrome P450 enzyme, CYP1B1. To overcome MDR, it has been suggested that P-glycoprotein be disabled from performing its efflux function. To date, many efforts have been made to design novel compounds that effectively modulate P-glycoprotein’s efflux function.

**Figure 25:**
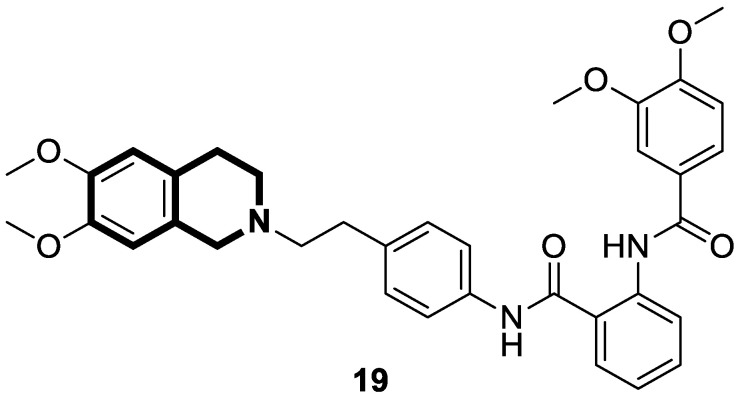
Tetrahydroisoquinoline-based third-generation P-gp inhibitor **19**.

Recently, several structural modifications of a third-generation P-gp inhibitor—*N*-(2-((4-(2-(6,7-dimethoxy-3,4-dihydroisoquinolin-2(1*H*)-yl)ethyl)phenyl)carbamoyl)phenyl)-3,4-dimethoxybenzamide (compound **19** or **WK-X-34**), depicted in Figure 25—led to the discovery of more potent compounds [84,85,86].

In 2024, based on pharmacophore hybridization, bioisosteric, and fragment-growing strategies, Dong and colleagues designed and synthesized eleven novel tetrahydroisoquinoline-benzo[*h*]chromen-4-one conjugates exhibiting dual glycoprotein-P and CYP1B1 inhibitory activity [87].

**Figure 26:**
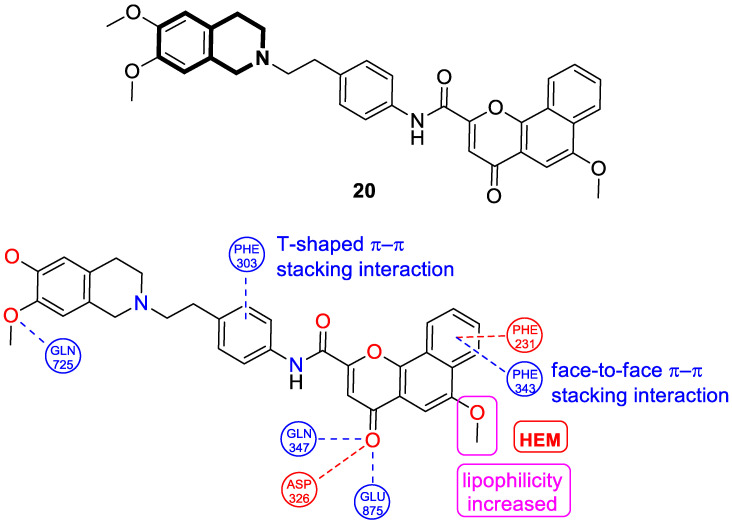
Tetrahydroisoquinoline-benzo[*h*]chromen-4-one conjugate **20**, exhibiting dual glycoprotein-P and CYP1B1 inhibitory activity, and the results of docking studies showing interactions between compound **20** and active domains of P-gp (blue) and CYP1B1 (red).

It has been shown that, in the colorectal cancer cell line (SW620/AD300), compound **20** displayed a drug resistance reversal potency similar to WK-X-34, a third-generation inhibitor (IC_50_ = 0.25 μM, RF = 44.4) (Figure 26). Moreover, **20** with Doxorubicin (**20** + DOX) at a concentration of 5 μM exhibited a remarkable drug resistance reversing activity (IC_50_ = 4.7 M, RF = 13.7) in the DOX-SW620/AD300-1B1 resistance line, which overexpressed ABCB1 and CYP1B1. Compound **20** was more potent than the non-selective CYP1B1 inhibitor α-naphthoflavone (ANF). Furthermore, the molecular docking studies showed hydrophobic, hydrogen-bonding, and face-to-face or T-shaped π–π stacking interactions between compound **20**, P-gp, and CYP1B1 (Figure 26). It was found that the molecule is stabilized in a hydrophobic pocket of P-glycoprotein formed by tryptophan, phenylalanine, alanine, and valine residues (Trp232, Phe303, Phe343, Phe770, Ala987, and Val991). A methoxyl substituent group on the benzo[*h*]chromen-4-one may increase the lipophilic character of the molecule, causing more effective hydrophobic pocket interactions. A face-to-face π–π stacking interaction was formed between the phenyl ring of benzo[*h*]chromen-4-one and phenylalanine 343; an extra T-shaped π–π stacking interaction was detected between the linking phenyl ring and phenylalanine 303. Additionally, in P-gp, hydrogen bonding was observed between benzo[*h*]chromen-4-one and glutamine (Gln347) and glutaminic acid (Glu875), as well as between the 1,2,3,4-tetrahydroisoquinoline ring and glutamine (Gln725). In the case of the CYP1B1 protein, compound **20** shares a hydrogen bond with Aspartic acid 326. The π–π stacking interaction was established between the benzo[*h*]chromen-4-one scaffold and the phenylalanine residue (Phe231). Docked molecule **20** extended towards the heme molecule via its 1,2,3,4-tetrahydroisoquinoline moiety. These results prove that this conjugate constitutes a reference scaffold for the further development of agents with MDR reversing ability.

Tangles and supercoils of DNA can be managed with type II topoisomerase (Top2), which cleaves both strands simultaneously. During DNA cleavage, Top2 employs its tyrosine residue, producing a transient Top2 cleavage complex (Top2cc) in which the enzyme is covalently bound by phosphoryl bonds. Top2 cuts DNA using its tyrosine residue, resulting in the formation of a transient Top2 cleavage complex in which the enzyme is covalently bound to the DNA via phosphoryl bonds. Specifically, Top2 poisons like Etoposide (ETP), Teniposide, and Doxorubicin are widely used as second-line treatments for platinum-resistant ovarian cancers. These chemotherapeutics bind to Top2cc and stabilize it, causing DNA double-strand breaks (DSBs). In a pharmacological sense, it depends largely on the quantity of trapped cross-links between Top2-DNA. Topoisomerase II (Top2)-mediated DNA double-strand breaks (DSBs) are repaired for tyrosyl-DNA phosphodiesterase 2 (TDP2), which undermines the anticancer mechanism of Top2 poisons such as Etoposide (ETP). It has been suggested that inhibiting TDP2 could expose cancer cells to TOP2 agents by increasing Top2 cleavage complex levels. The DSBs, however, usually trigger repair pathways, which can reduce sensitivity as a consequence. In addition, tyrosyl-DNA phosphodiesterase 2 (TDP2) is the only known human enzyme capable of selectively cleaving the 5′ phosphotyrosyl covalent bond to initiate Top2cc repair. Unfortunately, it appears that many TDP2 inhibitors possess a moderate activity and poor characterization, or do not display drug-like properties. To date, only a few reports on TDP2 inhibitors can be found in the literature.

**Figure 27:**
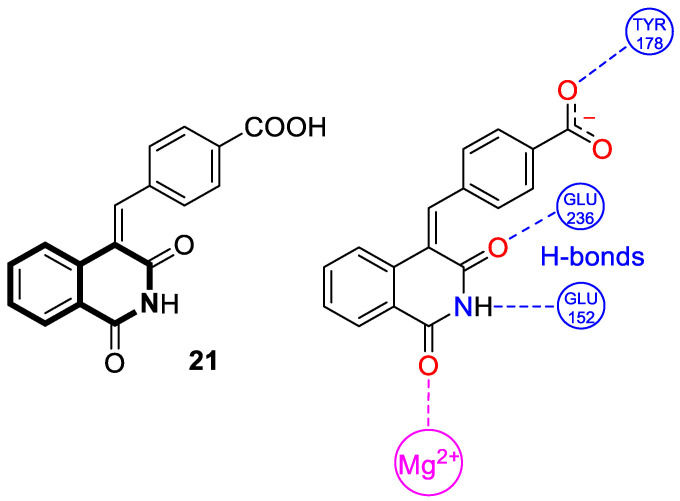
TDP2 inhibitor isoquinoline-1,3(2*H*,4*H*)-dione derivative **21** and the binding mode of benzylideneisoquinoline-1,3(2*H*,4*H*)-dione **21** with TDP2 protein.

Recently, the isoquinoline-1,3-dione scaffold was identified as a novel chemotype for constructing selective TDP2 inhibitors. However, the reported structure–activity relationship was limited to simple substitution patterns [88]. Senaweera and co-workers synthesized and tested a series of 50 analogs modified at positions N-2 and C-4 of isoquinoline-1,3(2*H*,4*H*)-dione [89]. It was found that the benzylidene-substituted derivatives showed a low micromolar potency. The highest TDP2 inhibition was demonstrated for 4-((1,3-dioxo-2,3-dihydroisoquinolin-4(1*H*)-ylidene)methyl)benzoic acid (**21**) (Figure 27), which achieved an IC_50_ value of 4.8 μM.

The authors performed molecular modeling with numerous mouse TDP2 (mTDP2) and human TDP2 (hTDP2) crystal structures with bound ligands and a high-resolution hTDP2 structure with a metal ion at the active site. Based on the results of a detailed docking analysis, the binding mode for the most prominent benzylideneisoquinoline-1,3(2*H*,4*H*)-dione **21** was predicted. It was found that both the NH group and the two carbonyl groups (C=O) of the isoquinoline-1,3-dione core are crucial to H-bond formation with glutminic acid residues (GLU 152 and 236) and the coordination of magnesium ions (C-1-carbonyl group). On the other hand, the carboxylate anion located on the terminal phenyl ring interacts with tyrosine residue (TYR 178) (Figure 27).

**Figure 28:**
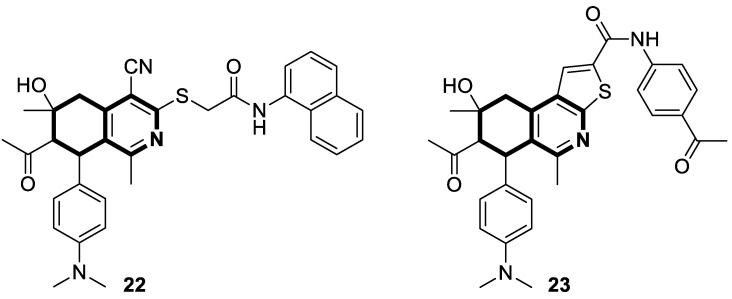
5,6,7,8-tetrahydroisoquinoline **22** and 6,7,8,9-tetrahydrothieno[2,3-*c*]isoquinoline **23** with anticancer activity, designed by Sayed.

Recently, Sayed and co-workers [90] synthesized novel 5,6,7,8-tetrahydroisoquinolines and 6,7,8,9-tetrahydrothieno[2,3-*c*]isoquinolines to evaluate their anticancer activity towards two cell lines: the A459 cell line (lung cancer cells) and MCF7 cell line (breast cancer cells) (Figure 28).

Compared with reference Doxorubicin, isoquinoline derivative **22** displayed the highest level of cytotoxic activity against the adenocarcinoma cell line (A549), while compound **23** showed the highest potency against the human breast cancer cell line (MCF7). The estimated IC_50_ values were 0.155 µM and IC_50_ 0.170 µM, respectively. Furthermore, the impact of compounds **22** and **23** on the growth of A549 and MCF7 cell lines was also investigated using flow cytometry and Annexin V-FITC apoptotic assays. Based on the results, compound **22** interrupted the G2/M phase of the cell cycle, causing a 79-fold increased apoptosis in A459 cells than in control cells, while 6,7,8,9-tetrahydrothieno[2,3-*c*]isoquinoline derivative **23** caused cell cycle arrest at the S-phase, with a 69-fold increase in apoptosis in the MCF7 cell line. Authors claimed that compound **22** behaves as a potent cyclin-dependent kinase 2 (CDK2) inhibitor, with an IC_50_ of 0.149 µM, whereas compound **23** significantly inhibits dihydrofolate reductase (DHFR), with an IC_50_ of 0.199 µM. Experimental findings were also supported by in silico docking studies.

**Figure 29:**
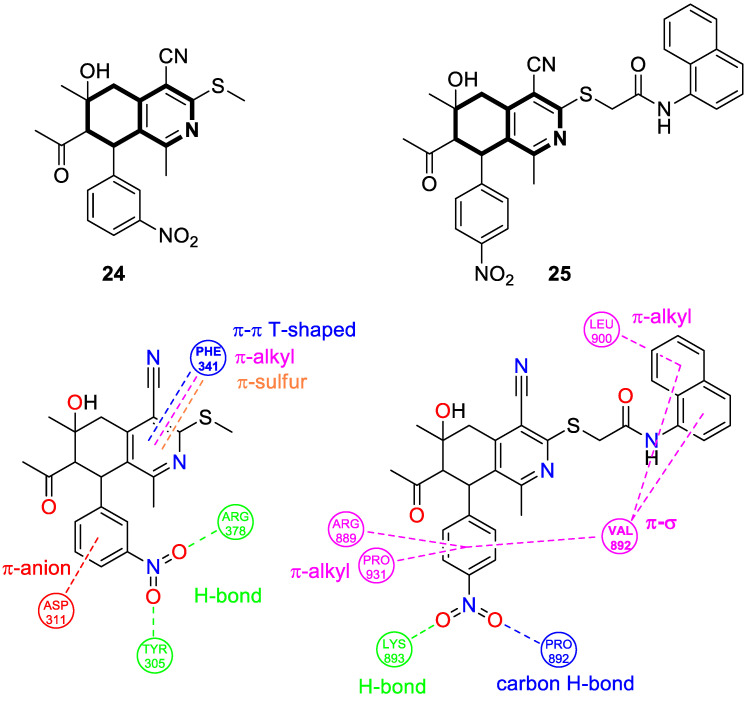
Tetrahydroisoquinolines bearing nitrophenyl group targeting HSP90 and RET enzyme, and docking results of compound **24** to chaperone HSP90 (**left**) and compound **25** to RET enzyme (**right**).

More recently, in 2025, Bakhite and colleagues synthesized analogous tetrahydroisoquinolines bearing a nitrophenyl group that target heat shock protein 90 (HSP90) and the RET enzyme (Figure 29) [91]. The compounds were evaluated for their antitumor activities against breast and liver cancer cell lines. It should be pointed out that many conditionally activated signaling proteins, as well as a number of mutant or overexpressed signaling proteins that promote the survival or proliferation of cancer cells, require the molecular chaperone HSP90 to function and maintain their stability. The interaction with HSP90 maintains their proper conformation and activity, which is essential to cancer cell growth. Therefore, HSP90 represents an important target for cancer therapies, as it can destabilize and degrade cancer-promoting proteins when inhibited.

Based on the in vitro test results, compound **24** (Figure 29) demonstrated cytotoxic activity, especially on HEPG2 cells, with an IC_50_ value of 75 μg/mL. The annexin V-FITC test and flow cytometry revealed that compound **24** in HEPG2 cells induced a 59-fold increase in apoptosis and cell cycle arrest at the G0–G1 and G2/M phases. Compound **25** (Figure 29) displayed the strongest antiproliferative activity against the MCF7 cell line, with an IC_50_ of 12 μg/mL. The molecular docking results showed that compound **24** exhibits a strong binding affinity with heat shock protein HSP90, with a relatively small difference in comparison to the reference HSP90 inhibitor (ΔG = −6.8 kcal/mol vs. Onalespib ΔG = −7.1 kcal/mol). Further in silico simulations of possible interactions revealed that the protein–compound **24** complex may be stabilized by three conventional hydrogen bonds with tyrosine 305 and arginine 378 residues, with distances ranging from 2.41 Å to 2.71 Å. In addition, compound **24** interacted with aspartic acid 311 (π-anion, 4.04 Å) and formed π-sulfur (5.27 Å), hydrophobic π-π T-shaped, and π-alkyl interactions with phenylalanine residue 341 (Figure 29). Compound **25** binds to a tyrosine kinase-type receptor (rearranged during transfection, RET) with a Gibbs free energy (∆G) of −6.8 kcal/mol. Reference Alectinib—a selective RET inhibitor—exhibited a binding energy of −7.2 kcal/mol. The cell signaling pathways controlled by RET are essential for cell growth, differentiation, and survival. The inhibition of RET enhances antiproliferative activity in several cancers. Compound **25** formed a classical hydrogen bond with lysine 893, a non-classical carbon–hydrogen bond with proline residue 892, a π-σ bond with valine 892, and π-alkyl bonds with arginine 889 and proline 931 residues (Figure 29).

**Figure 30:**
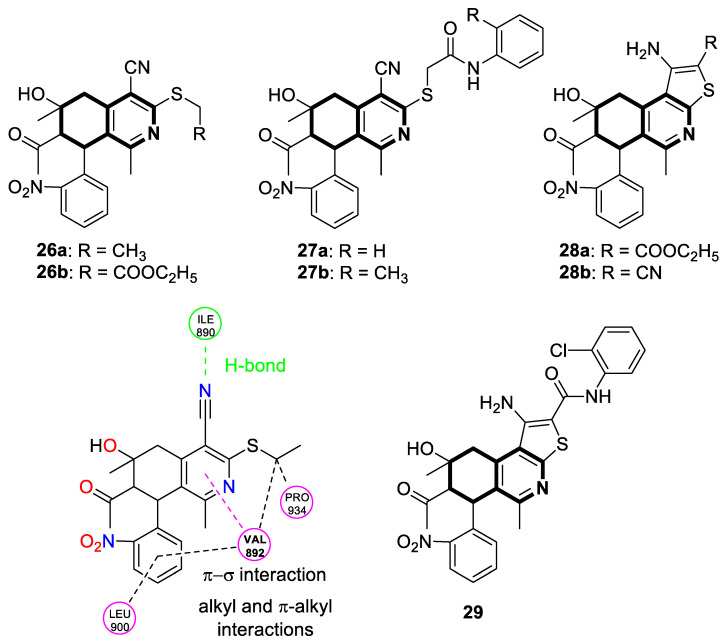
Cytotoxic 5,6,7,8-tetrahydroisoquinolines and 6,7,8,9-tetrahydrothieno[2,3-*c*]isoquinolines **26a**, **26b**, **27a**, **27b**, **28a**, **28b**, and **29**, designed by Saddik. In silico results obtained for 7-acetyl-3-(ethylthio)-6-hydroxy-1,6-dimethyl-8-(2-nitrophenyl)-5,6,7,8-tetrahydroisoquinoline-4-carbonitrile (**26a**) docked into RET protein.

In 2025, Saddik and colleagues reported two series of 5,6,7,8-tetrahydroisoquinolines and 6,7,8,9-tetrahydrothieno[2,3-*c*]isoquinolines to assess their anticancer potential on eight tumor cell lines and the safety profile using a normal human skin fibroblast cell line [92]. In comparison with the positive control—Doxorubicin—compounds **26a**, **26b**, **28a**, and **29** (Figure 30) showed the highest levels of cytotoxicity against the human liver cell line (HEPG2), with IC_50_ values ranging from 31 to 42 µg/mL. Among all of the isoquinolines tested, **27a**, **27b**, and **28b** had the most considerable IC_50_ values against the colon cancer cell line (HCT116) (IC_50_ = 50, 55, and 49 µg/mL, respectively).

It was evident that, after treatment with the most potent compound **26a** at an IC_50_ concentration of 31 µg/mL, the population of HEPG2 cells in the G2/M phase increased from 12.12% (in control cells) to 19.45%. A significant increase in the percentage of apoptotic cells was found at the early stage (from 0.31% to 15.3%) and at the late stage (from 0.20% to 11.41%). Furthermore, 5,6,7,8-tetrahydroisoquinoline-4-carbonitrile **26a** was docked into the RET receptor (rearranged during transfection), which is a key component of the cell-signaling pathway that regulates growth, differentiation, and survival. The docking study indicated a favorable binding interaction, with a Gibbs free energy ∆G value of −5.2 kcal/mol. Compound **26a** forms a hydrogen bond with isoleucine 890, with π-σ, alkyl–alkyl, and hydrophobic interactions (Figure 30). As part of the cell signaling pathway involved in growth, differentiation, and survival, RET plays a key role. Even though the synthesized compounds displayed a moderate overall selectivity, the selectivity indexes for compounds **26a**, **26b**, and **28a** were higher than 2. Consequently, these compounds prefer cancerous cells, making them potential candidates for further development.

**Figure 31:**
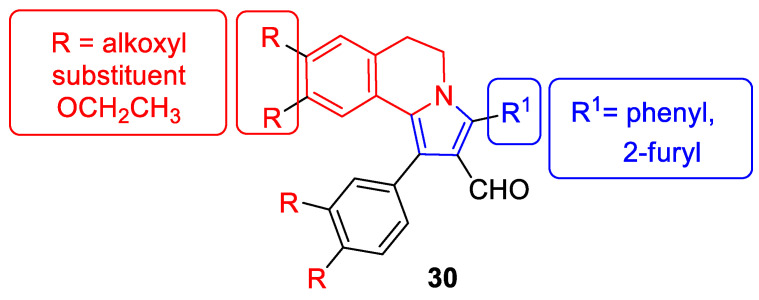
P-gp inhibitors **30** designed by Nevskaya [98].

There is significant interest in heterocyclic systems containing fused pyrrole and tetrahydroisoquinoline rings as potential anticancer drugs [93,94,95,96,97]. Based on these premises, Nevskaya et al. reported a novel class of 5,6-dihydropyrrolo[2,1-*a*]isoquinolines that prove to be selective P-glycoprotein (P-pg) efflux pump or multidrug resistance-associated protein 1 (MRP1) inhibitors **30** (Figure 31). Such P-gp and MRP1 modulators display the in vitro ability to reverse multidrug resistance in a Doxorubicin-resistant tumor cell model, reaching submicromolar IC_50_ values [98].

**Figure 32:**
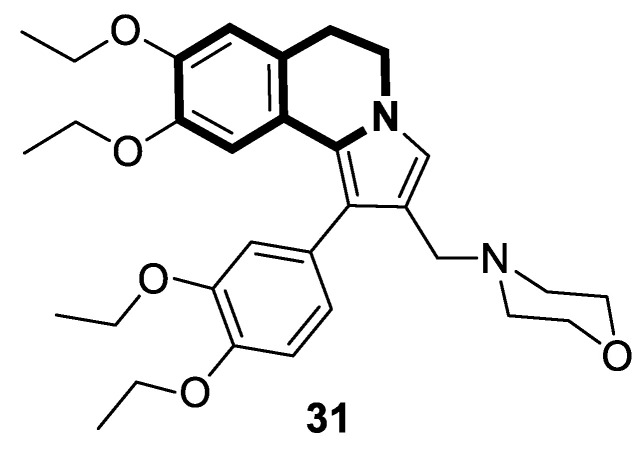
Novel P-gp inhibitor designed by Nevskaya: 4-((1-(3,4-diethoxyphenyl)-8,9-diethoxy-5,6-dihydropyrrolo[2,1-*a*]isoquinolin-2-yl)methyl)morpholine (**31**).

Another series of nature-inspired aza-heterocycles exhibiting significant antiproliferative activity and P-gp/MRP1 inhibition potency include derivatives based on the 1-phenyl-5,6-dihydropyrrolo[2,1-*a*]isoquinoline scaffold (1-Ph-DHPIQ) [99]. An example of a compound with antitumor potency in the submicromolar range of concentrations (IC_50_ < 20 μM) presented here is a DHPIQ bearing the basic 2-morpholinomethyl chain at C2 (Figure 32). Moderately selective Mannich base **31** prepared as water soluble HCl-salt (morpholinium chloride), stable at pH 2 and 7.4, was approximately 30-fold more potent against P-gp (IC_50_ = 0.45 µM P-gp vs. IC_50_ = 12.1 µM MRP1).

**Figure 33:**
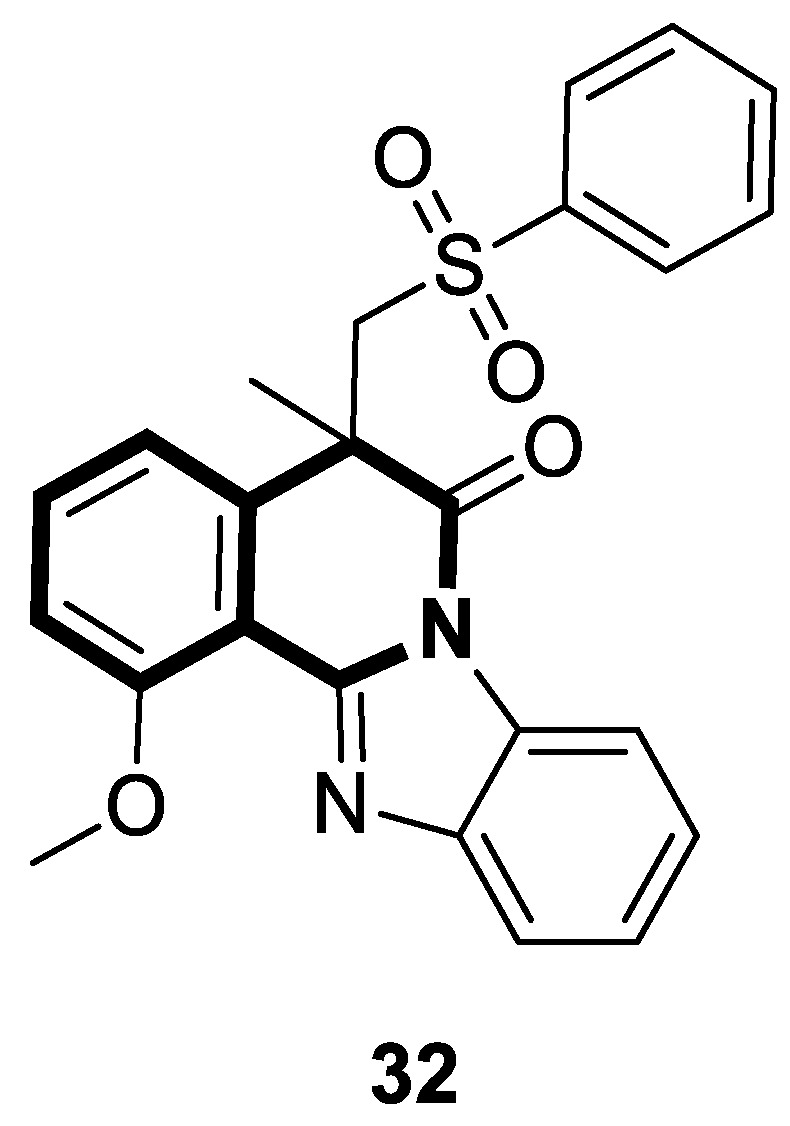
Benzimidazo[2,1-*a*]isoquinolin-6(5*H*)-one derivative **32** with antitumor activity.

A series of sulfonated indolo[2,1-*a*]isoquinolines and benzimidazo[2,1-*a*]isoquinolin-6(5*H*)-ones have been designed via a facile copper(II) bromide-induced radical-transmitted addition/cyclization starting from alkene derivatives and di-substituted-thiosulfonates. These sulfonated derivatives, incorporating isoquinoline, have been explored in vitro for antitumor activity using a cell counting colorimetric assay to determine the cell viability and proliferation of MGC-803, T-24, and HeLa tumor cell lines and Paclitaxel as a positive reference drug. The most prominent compound was benzo[4,5]imidazo[2,1-*a*]isoquinolin-6(5*H*)-one (**32**), which exhibited a potent inhibitory activity against human gastric cancer cells (MGC-803), with an IC_50_ value of 4.0 μM (Figure 33) [100].

**Figure 34:**
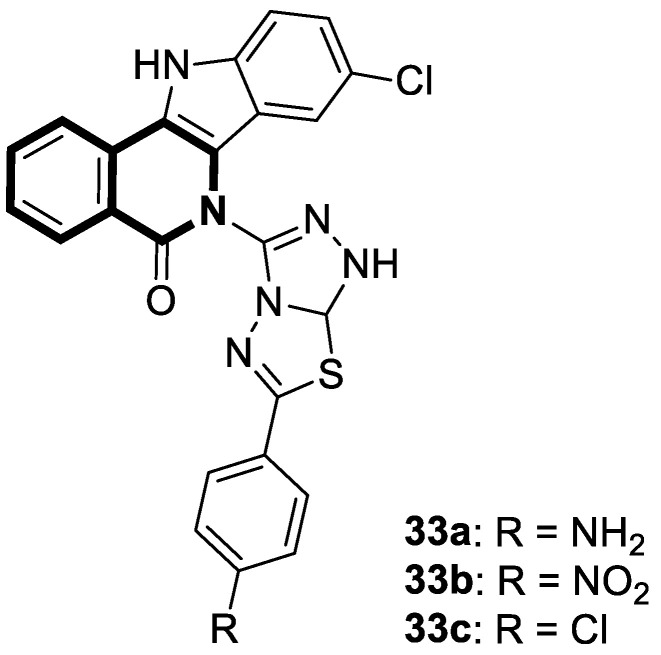
The promising anticancer drug candidates—8-chloro-6-(6-phenyl-1,7*a*-dihydro-[1,2,4]triazolo[3,4-*b*][1,3,4]thiadiazol-3-yl)-6,11-dihydro-5*H*-indolo[3,2-*c*]isoquinolin-5-one derivatives **33a**, **33b**, and **33c**.

Verma and co-workers have synthesized interesting indolo[3,2-*c*]isoquinolinyl[1,2,4]triazolo[3,4-*b*][1,3,4]thiadiazole derivatives whose cytotoxic effects have been evaluated on four different human cancer cell lines, such as MCF-7 (breast), A549 (lung), HeLa (cervical), and Panc-1 (pancreas) [101]. The results indicate that two compounds, **33a** and **33c** (Figure 34), might be the most promising in combating tumors. These derivatives showed a broad cytotoxic activity against MCF-7, A-549, HeLa, and Panc-1, achieving the following IC_50_ values: 0.43 µM, 0.42 µM, 0.55 µM, and 1.15 µM (for compound **33b**) and 0.32 µM, 0.43 µM, 0.51 µM, and 1.11 µM (for compound **33c**). The calculated IC_50_ values for reference Doxorubicin were 0.46 µM, 0.49 µM, 0.56 µM, and 1.17 µM, respectively. A significant cytotoxic effect was also observed for compound **33a** (Figure 34) in the context of MCF-7 and Panc-1, with IC_50_ values equal to and the same as the standard, 0.46 µM and 1.17 µM, respectively. The electron-withdrawing groups (-Cl, -NO_2_) present in their structures might be significant for cytotoxic activity. By inducing a dipole moment, these groups may increase water solubility and facilitate drug–target interactions.

**Figure 35:**
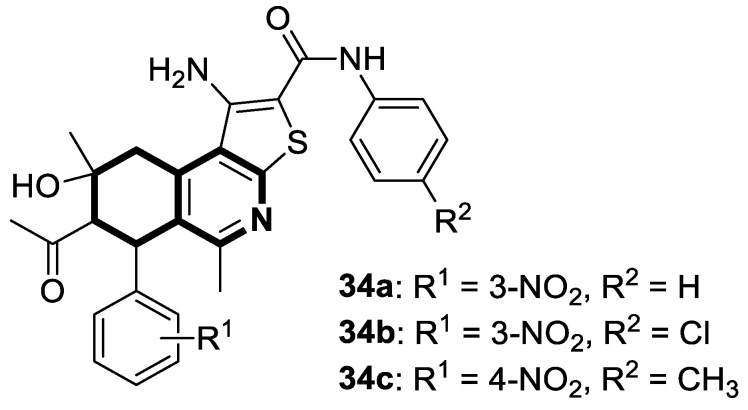
Cytotoxic 6,7,8,9-tetrahydrothieno[2,3-*c*]isoquinoline-2-carboxamides **34a**, **34b**, and **34c**.

In 2022, an interesting series of 6,7,8,9-tetrahydrothieno[2,3-*c*]isoquinoline-2-carboxamides were designed by Sayed et al. as potent anticancer agents [102]. Among them, three derivatives, **34a**, **34b**, and **34c** (Figure 35), at a concentration of 100 µM in an MTT test displayed cytotoxicity (IC_50_ = 34.9–57.6 µM) against the lung carcinoma cell line (A549) compared to the reference Doxorubicin (IC_50_ = 54.8 µM).

Moreover, 7-acetyl-1-amino-8-hydroxy-5,8-dimethyl-6-(3-nitrophenyl)-*N*-phenyl-6,7,8,9-tetrahydrothieno[2,3-*c*]isoquinoline-2-carboxamide (**34a**) in the DPPH assay proved to be a potent antioxidant. Compound **34a** at a concentration of 0.1 µg/mL showed a high scavenging activity of 92.2%.

**Figure 36:**
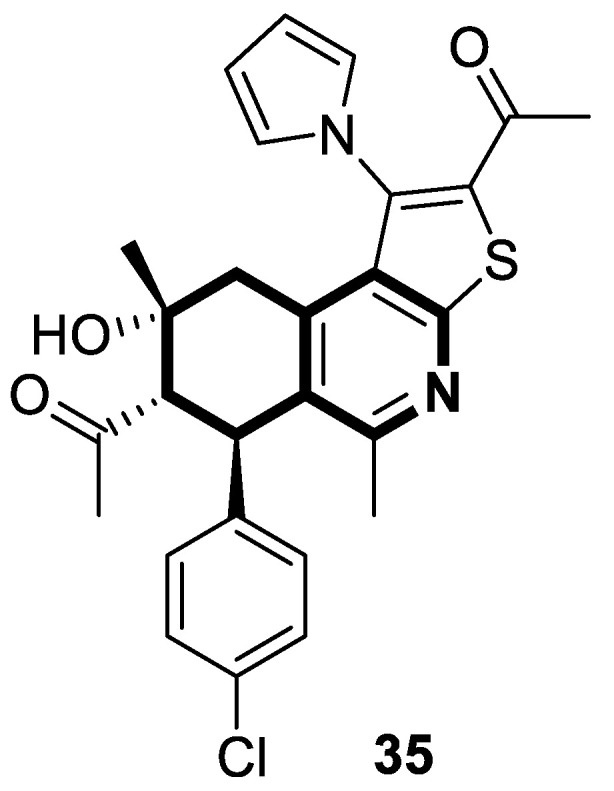
Thienoisoquinoline derivative **35** with anti-HepG2 potency.

In 2023, Marae et al. disclosed the above-mentioned chemotype containing an anticancer thieno[2,3-*c*]pyridine scaffold through a molecular merging strategy [103]. According to these studies, thienoisoquinolines were examined against HepG2 cells seeking curative interventions targeting hepatocellular carcinoma (HCC). Importantly, clinically used drugs against HCC are still deemed ineffective due to their high toxicity and limited efficacy. The discovery of the lead compound **35** (Figure 36) was facilitated through structural optimizations involving the introduction of acetyl and hydroxyl groups, a pyrrole ring, and *p*-halogen substituted phenyl.

Although the estimated mean IC_50_ value of thienoisoquinoline derivative **35** against HepG2 cells was higher than those estimated for the reference antineoplastic drugs Doxorubicine and Colchicine (IC_50_ = 168.59 µM vs. IC_50_ = 22.09 µM and IC_50_ = 0.65 µM, respectively), compound **35** retained a safe therapeutic profile towards non-cancerous Vero cells (cytotoxicity of 1.3% vs. 99.99% for Colchicine). Based on flow cytometry and Annexin/PI staining, it was demonstrated that compound **35** arrests HepG2 cells in the G2/M phase and increases cellular apoptosis. Further investigation revealed that compound **35** displayed in vitro tubulin polymerization inhibitory properties. The calculated IC_50_ value of 71 μM was comparable with that achieved for a potent tubulin inhibitor, Colchicine (IC_50_ = 14 μM). Finally, molecular docking simulations on the Colchicine binding site (CBS) of tubulin successfully reflect the potency of the synthesized derivative **35**. The authors suggested that the possible cytotoxicity of compound **35** against HepG2 cells might be connected with its tubulin inhibitory mechanism.

**Figure 37:**
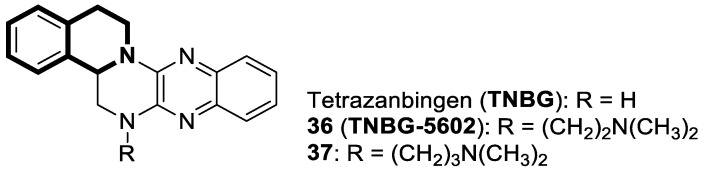
TNBG and its analogs: compounds **36** (**TNBG-5602**) and **37**.

In recent years, it was found that tetrazabingen—5,6,13,14-tetrahydro-4b*H*-6,7,12,12*b*-tetraaza-benzo[*b*]chrysene (**TNBG**, Figure 37)—belonging to sterol-isoquinoline derivatives has a potential chemotherapeutic activity [104]. It has been demonstrated that TNBG induces S-phase arrest and apoptosis in cancer cells. Moreover, **TNBG** has a strong ability to stimulate lipid accumulation. Recent studies have also revealed that induced lipid storage in cells may be connected to peroxisome proliferator-activated receptor gamma (PPARγ), which controls lipid uptake, storage, transport, and metabolism through fat-specific gene expression. It has been shown that excess lipids disturb fundamental cellular processes by interfering with the cellular substructures, leading to lipoapoptosis in differentiated cancer cells. In this way, a promising cancer treatments is lipoapoptosis induction in tumor cells. Since Tetrazanbigen (**TNBG**) possessed a poor water solubility, this compound did not meet the criteria for a candidate compound; efforts are made to obtain compounds that contain hydrophylic groups [105,106]. For example, Gan et al. synthesized a series of **TNBG** derivatives that incorporate flexible linkers with diverse hydrophilic substituents attached to them. It was found that such modifications may enhance both water solubility and antiproliferative potency, e.g., compound **36** (**TNBG-5602**), with a -CH_2_CH_2_- linker and *N*,*N*-dimethyl group, shown in Figure 37 [107].

In this study, Gan identified that the analog which contained a linker with three carbon atoms—3-(14,15-dihydro-4b*H*-isoquinolino[2′,1′:1,6]pyrazino[2,3-*b*]quinoxalin-6(5*H*)-yl)-*N*,*N*-dimethylpropan-1-amine (**37**) (Figure 37)—displayed the highest potency. The antiproliferative activity of the compound was evaluated against human cancer cell lines, including hepatoma (HepG2 and QGY-7701), colon (LOVO), and lung adenocarcinoma epithelial (A549) cells. It was shown that analog **37** exhibited a higher potency than **TNBG**, **TNBG-5602**, Sorafenib, and Erlotinib, which were used as positive controls in standard assays (IC_50_ values for **37** were in the range of 0.54–1.09 µM vs. IC_50_ for control drugs of 6.95–20.86 µM). Moreover, compound **37** blocked A549 cell colony formation through cytoplasmic vacuolation and lipid accumulation. According to the obtained results, derivative **37** is a partial agonist of PPARγ. It was demonstrated that **37** inhibits AKT phosphorylation and triggers apoptosis in A549 and HepG2 cells. Furthermore, in xenograft models, this compound at a dose of 10 mg/kg effectively reduced tumor growth by approximately 62%. Overall, the authors’ findings suggest that the promising **TNBG** analog **37** may be a lead structure for designing novel anticancer agents.

**Figure 38:**
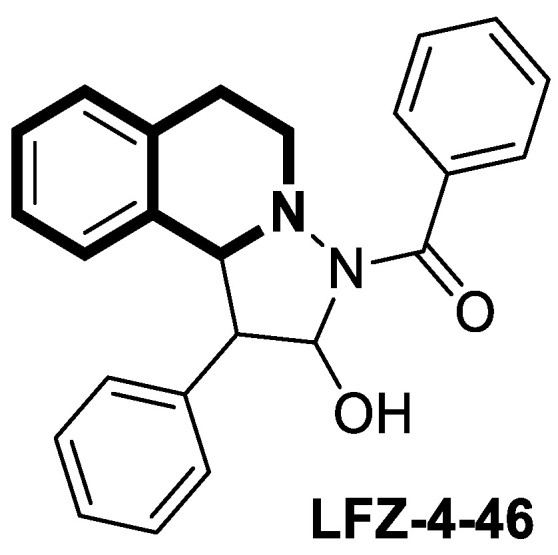
Tetrahydropyrazolo[5,1-*a*]isoquinoline derivative **LFZ-4-46** that induces apoptosis and cell cycle arrest via activation of MAPK pathway.

Xu and co-workers found that the tetrahydropyrazolo[5,1-*a*]isoquinoline derivative **LFZ-4-46** (Figure 38) activates the caspase-dependent apoptosis and mitogen-activated protein kinase (MAPK) pathways [108].

Compound **LFZ-4-46** remarkably inhibits in vitro the viability of human breast cancer cells (T47D, IC_50_ = 26.73 μM) and human prostate cancer cells (PC3, IC_50_ = 23.81 μM and DU145, IC_50_ = 36.44 μM). It causes G2/M phase cell cycle arrest and triggers DNA damage. Moreover, the in vivo antitumor effect of **LFZ-4-46** was confirmed in a xenograft BALB/c male nude mouse model of prostate cancer. These data suggest that **LFZ-4-46** may serve as a promising lead structure for developing novel antitumor drugs.

The unique structure of chalcones and their extensive biological activity make them intriguing candidates for potent drugs [109], especially chemotherapeutic agents designed to fight cancers [110,111,112,113].

**Figure 39:**
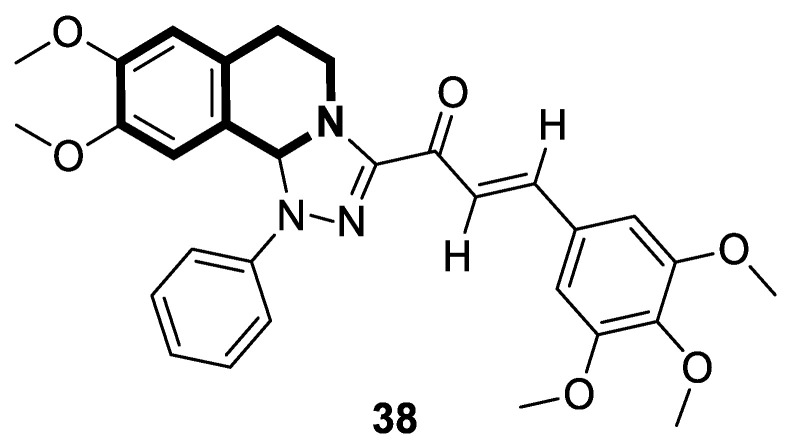
Isoquinoline-based chalcone **38** exhibiting anticancer activity.

In light of the fact that chalcones possess a promising antiproliferative activity, WalyEldeen and collaborators [114] examined the in vivo potency of a designed synthetic isoquinoline chalcone-based compound—1-(8,9-dimethoxy-1-phenyl-1,5,6,10*b*-tetrahydro-[1,2,4]triazolo[3,4-*a*]isoquinolin-3-yl)-3-(3,4,5-trimethoxyphenyl)prop-2-en-1-one (**38**)—depicted in Figure 39.

According to the author’s findings, an important mechanism of **38**, which exhibits antitumor activity, is the induction of oxidative stress and DNA damage, which lead to cell death. Upon the treatment of female BALB/c mice inoculated with Ehrlich ascites carcinoma cells with compound **38**, tumor weight decreased significantly. The total antioxidant concentration in tumor tissue was substantially depleted, resulting in increased oxidative stress and DNA damage. Moreover, the pro-apoptotic genes for proteins p53 and Bax were upregulated. The pilot studies also indicated that the maximum tolerated dose of the tested compound, adjusted for body weight, was 428 mg/kg. The histopathological examination revealed that treatment with **38** is not as toxic on the liver and kidneys as the reference drug—Doxorubicin, administered intraperitoneally (DOX; 4 mg/kg). Therefore, **38** could be a promising option for treating solid tumors.

**Figure 40:**
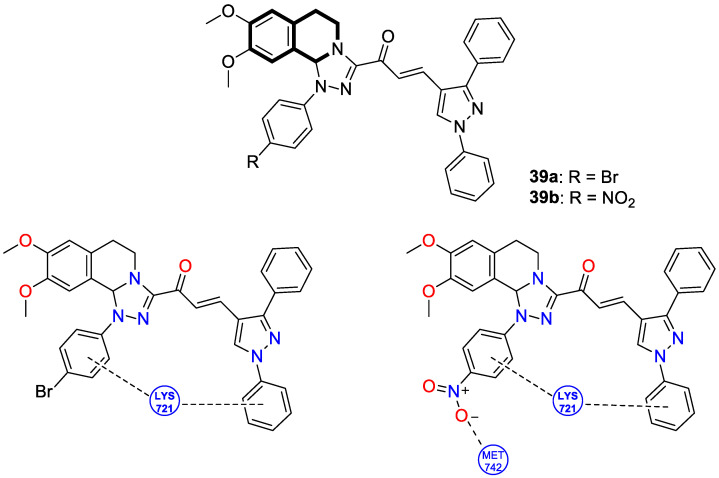
EGFR inhibitors—chalcones tethered through [1,2,4]triazolo[3,4-*a*]isoquinoline and 1,3-diphenyl-1*H*-pyrazole **39a** and **39b**, and interactions of compounds **39a** and **39b** with active site of EGFR protein (PDB ID: 1M17).

Utilizing the molecular hybridization approach, chalcones tethered through a [1,2,4]triazolo[3,4-*a*]isoquinoline and 1,3-diphenyl-1*H*-pyrazole scaffold were designed and synthesized in 2024 by Abdelaal and colleagues [115]. These novel hybrids were assessed for their cytotoxic activity against a panel of human cancer cell lines, A549, HCT116, PC3, HT29, and MCF-7, in comparison with the non-tumorogenic human lung cell line WI-38. In particular, compounds **39a** and **39b** (Figure 40) exhibited cytotoxic properties toward non-small-cell lung cancer (A549), with IC_50_ values of 2.3 μM and 1.15 μM, respectively. In contrast, when WI-38 cells were taken as a reference, minimal cytotoxicity was observed.

The tested compounds were found to be the most potent EGFR inhibitors, with IC_50_ = 0.031 μM and 0.023 μM, respectively. Particularly, triazolo[3,4-*a*]isoquinolines **39a** and **39b** demonstrated a high potency against two EGFR mutations—EGFR-L858R and EGFR-T790M, as well as the wild-type of EGFR (EGFR-wt). It should be noted that both compounds displayed selectivity against the EGFRL858R mutation. In comparison with Lapatinib, compound **39b** had a selectivity index for EGFR^T790M^-mutant NSCLC that was 81 times higher. Furthermore, triazolo[3,4-*a*]isoquinolines **39a** and **39b** promote cell cycle arrest in G2/M and pre-G1 phases and downregulate the anti-apoptotic protein Bcl2, while upregulating pro-apoptotic proteins p53, Bax, and caspases-3, -8, and -9.

In silico studies revealed that compound **39b** had the highest affinity for the active site of the EGFR protein (PDB ID: 1M17), with a binding score of −9.086 kcal/mol. This derivative forms a NO_2_-hydrogen bond with methionine 742, with a bond distance of 3.3 Å. The interaction also included lysine 721—one π-H with a bond distance of 3.66 Å and binding energy of −0.8 kcal/mol, and two π-cations with bond distances of 4.31 Å and 4.65 Å and binding energies of −0.6 kcal/mol and −1.8 kcal/mol, respectively (Figure 39). Compound **39a** exhibited the second highest binding score of −8.412 kcal/mol and an interaction through lysine residue 721—a π-H interaction with a bond distance of 3.73 Å and binding energy of −0.7 kcal/mol and two π-cation interactions with bond distances of 4.21 and 4.68 Å and energies of −0.8 kcal/mol and −1.7 kcal/mol, respectively (Figure 40). The authors claim that synthesized isoquinoline-based chalcones **39a** and **39b** may represent preliminary leads for the design of more potent EGFR inhibitors.

To date, many functional modifications have been reported for tetrahydro[1,2,4]triazolo[3,4-*a*]isoquinoline-based chalcones. Darwish and colleagues synthesized analogous methoxy derivatives that produced Luc-4T1 cell cycle arrest, increased Bax/Bcl2 mRNA ratios, and activated caspases-3 and -7. These chalcones can be considered potent antineoplastic agents for the treatment of breast cancer [116]. Another example of potential anti-cervical cancer drugs are promising chalcones incorporating thiadiazolyl-isoquinoline obtained by I.A. Abdelhamid’s team [117] or 5,6-dihydropyrrolo[2,1-*a*]isoquinoline-based chalcones designed by Teleb and collaborators [118].

**Figure 41:**
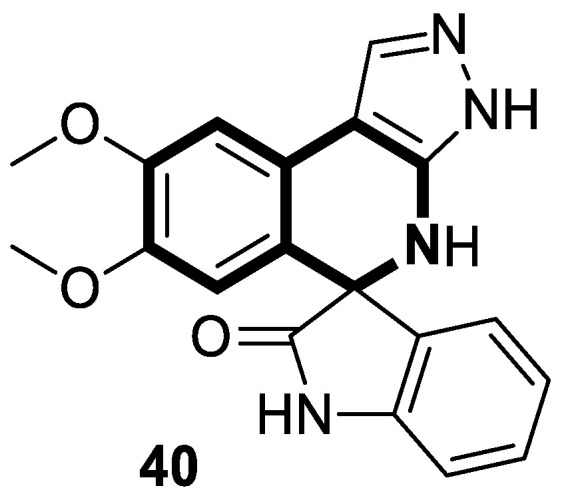
Spiroindolone derivative—7′,8′-dimethoxy-1′,3′-dimethyl-3′,4′-dihydrospiro[indoline-3,5′-pyrazolo[3,4-*c*]isoquinolin]-2-ones (**40**)—synthesized via a modified Pictet–Spengler reaction.

In 2023, the anticancer and immunomodulatory potential of 7′,8′-dimethoxy-3′,4′-dihydrospiro[indoline-3,5′-pyrazolo[3,4-*c*]isoquinolin]-2-one (**40**) (Figure 41), obtained via a modified Pictet–Spengler reaction (PSR), was investigated by de Castro Alves et al. [119].

The selectivity of compound **40** towards the myelogenous leukemia cell line (K562) was evaluated using non-cancerous peripheral blood mononuclear cells (PBMC) and the Vero kidney epithelial cell line. It was found that spiroindolone **40** exerted cytotoxic effects on K562 cells, with an IC_50_ value of 25.27 µg/mL, compared to untreated control cells. A colony formation test showed that this derivative inhibits the clonal proliferation of leukemia cells, with a minimal toxic effect on non-cancerous cells. It was demonstrated that spiroindolone inhibits leukemia cells’ entry into mitosis through the induction of S and G2/M cell cycle arrest. Furthermore, compound **40** also has the ability to modulate the immune response by upregulating the production of pleiotropic cytokine IL-6 (Interleukin-6) and IL-12/23p40. It should be noted that, in chronic myeloid leukemia, IL-6 has a crucial role in promoting tumor growth by increasing the number and size of cancer cells. Considering its potential therapeutic value in treating chronic myeloid leukemia and immunomodulatory effects, the spiroindolone-derived compound is a promising drug candidate.

**Figure 42:**
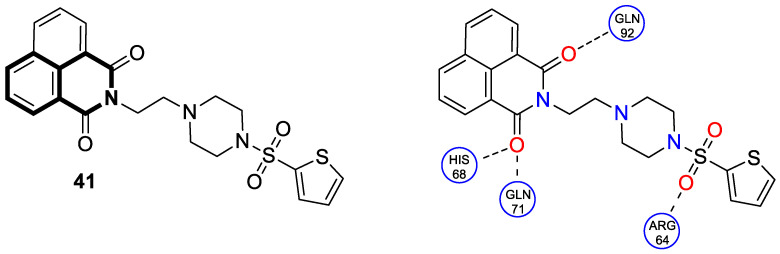
Compound **41**, displaying the highest cytotoxicity, and docking results for compound **41** showing binding residual amino acids of the CAIX protein.

In 2023, Haque reported a two-step synthesis of piperazine-linked 1,8-naphthalimide-arylsulfonyl derivatives incorporating an isoquinoline scaffold, which were evaluated in vitro for cytotoxicity and as potential fluorescence imaging agents. These compounds were tested using the mouse fibroblast cell line (3T3) and the murine mammary carcinoma cell line derived from BALB/cfC3H mice (4T1). In terms of growth and metastasis, line 4T1 shares a close resemblance to human breast cancer [120]. According to imaging studies, the probes possessed green fluorescence, good membrane permeability, and were dispersed throughout the entire cytoplasm of the cells. It has been found that the obtained compounds are nontoxic to normal cells, with viability levels between 82% and 95% at a concentration of 1 μg/mL. Compounds with such high cellular viability are beneficial for staining cells and tissues. Their ability to bind to non-cancerous cells at such a low concentration also confirms their suitability for imaging cells. The most promising, 2-(2-(4-(thiophen-2-ylsulfonyl)piperazin-1-yl)ethyl)-1*H*-benzo[*de*]isoquinoline-1,3(2*H*)-dione (**41**) (Figure 42), was determined to be the most cytotoxic compound against 4T1 breast cancer cells (viability lower than 80% at 0.7 μM).

In a docking study, carbonic anhydrase isoform-IX (CAIX) in complex with **41** had a favorable binding affinity of −7.95 kcal/mol. These results are similar to the well-established CAIX inhibitor 4-(3-(4-fluorophenyl)ureido)benzenesulfonamide, with a binding affinity of −8.39 kcal/mol. Additionally, compound **41** formed four hydrogen bonds with the protein, involving the following residues: arginine 64, histidine 68, glutamine 71, and glutamine 92 (Figure 42). Researchers suggest that piperazine-linked 1,8-naphthalimide-arylsulfonyl derivatives may provide promising therapeutic and research targets for cancer.

The reader will find more about naphthalimide derivatives in recently published papers focusing on their anticancer activities or fluorescent probes with biomedical applications [121,122,123,124,125,126,127,128,129,130,131,132,133,134,135,136,137,138,139,140].

### 2.2. Antibacterial Agents

**Figure 43:**
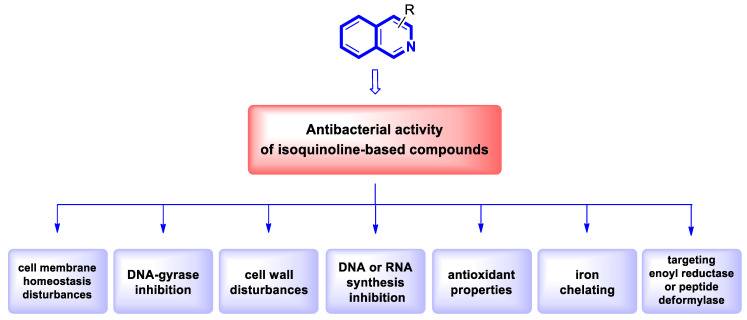
Possible modes of actions of antibacterial isoquinoline-based compounds.

Public health is particularly concerned about the microbes that cause most hospitals’ infections. It is becoming increasingly difficult to treat infections with commonly used antibiotics due to resistance. Infections caused by multidrug-resistant bacteria, for example, Gram-positive strains of methicillin-resistant *Staphylococcus aureus* (MRSA), Vancomycin-resistant *Staphylococcus aureus* (VRSA), or Vancomycin-resistant *Enterococcus faecium* (VRE *faecium*), are increasing because traditional antibiotics have been overused and new antibacterial classes are slowly being introduced. Despite advances in antimicrobial research, there are still limited therapeutic options. To successfully manage these multidrug-resistant strains, novel chemotherapeutics are needed. Notably, when it comes to finding novel antimicrobial drugs, special attention is being given to nitrogen-containing heterocyclic compounds [141]. Possible mechanisms of the antibacterial action of isoquinoline derivatives are depicted in Figure 43.

#### 2.2.1. Isoquinoline-Based Compounds

**Figure 44:**
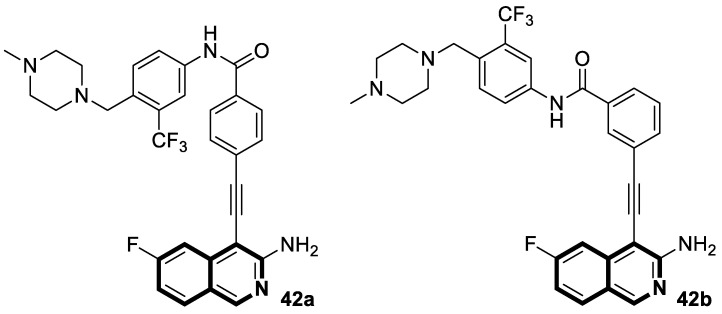
Alkenyl isoquinolines **42a** and **42b** exhibiting potent activity against MRSA.

Recently, Karanja and co-workers screened a library of alkynyl isoquinolines synthesized via Sonogashira coupling for potent broad spectrum Gram-positive antibacterial agents, including MRSA and VRSA [142]. The most active compounds in this class are **42a** and **42b** (Figure 44), which exhibit a potent activity against MRSA, VRE *faecium*, VRE *faecalis*, Methicillin-resistant *Streptococcus pneumoniae*, Cephalosporin-resistant *Streptococcus pneumoniae*, Methicillin-resistant *Staphylococcus epidermis*, and Linezolid-resistant *Staphylococcus aureus* (MICs = 4 μg/mL or 8 μg/mL). It was found that the isoquinoline present in the structure is essential for antibacterial potency. The replacement of the isoquinoline scaffold with a pyridine ring resulted in the loss of half of their activity.

Importantly, the identified alkynyl isoquinolines **42a** and **42b** exerted a potency against Ciprofloxacin-, Levofloxacin- and Norfloxacin-resistant *Staphylococcus aureus* (MICs = 4 µg/mL or 8 µg/mL). It was confirmed that both compounds **42a** and **42b** permeate macrophage cells. At the same concentration as Vancomycin, they reduce the burden of intracellular MRSA. The authors suggest that alkynyl isoquinolines may exert anti-*Staphylococcus aureus* activity through the cell wall disturbance or inhibition of DNA and RNA biosynthesis.

**Figure 45:**
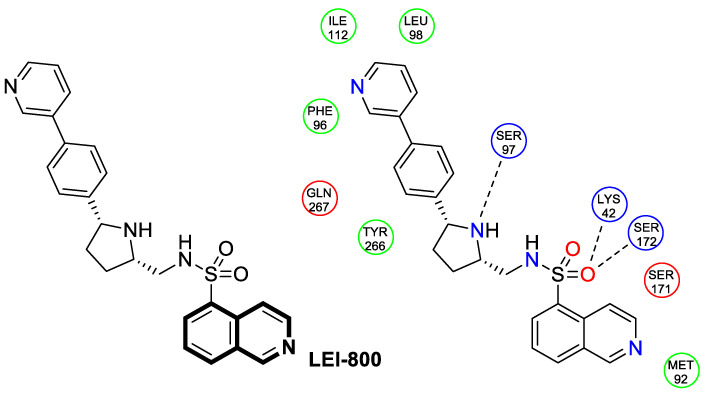
**LEI-800**—DNA-gyrase inhibitor and molecular interactions of **LEI-800** with GyrA.

Bakker and co-workers reported the discovery of a novel class of isoquinoline sulfonamides with potent antibiotic activity against clinically relevant Gram-negative bacteria [143]. The screening of a library of synthetic small molecules for activity against *Escherichia coli*, accompanied by a subsequent structure–activity relationship (SAR) analysis and a rational design program, led to a potent isoquinoline-bearing sulfonamide **LEI-800** (Figure 45), which achieved a minimum inhibitory concentration of 6.25 μM (2.6 μg/mL). This compound also exhibited an enhanced antimicrobial activity against the *Klebsiella pneumoniae* strain.

DNA-gyrase was identified as the molecular target of *N*-(((2S,5R)-5-(4-(pyridin-3-yl)phenyl)pyrrolidin-2-yl)methyl)isoquinoline-5-sulfonamide (**LEI-800**), and the diastereomer bearing the *cis*-configuration displayed a higher potency than the others. Clearly, the *cis*-enantiomer of **LEI-800** has a curved shape that matches the shape of the enzyme pocket. Cryogenic electron microscopy (cryo-EM) permitted the structure determination of a complex gyrase-**LEI-800** and DNA. It was demonstrated that **LEI-800** occupies an allosteric hydrophobic pocket in the GyrA subunit. Compared to other gyrase inhibitors, such fluoroquinolones, the compound **LEI-800** exhibits a unique mode of action. The pyridine ring is located between the isoleucine and phenylalanine of GyrA. Interestingly, **LEI-800**, through its pyrrolidine ring and sulfone moiety, forms hydrogen bonds. It was revealed that the gyrase pocket to which **LEI-800** binds is not targeted by other well-known inhibitors. **LEI-800** binding did not affect the interaction with double-stranded DNA, as was observed in the case of Ciprofloxacin, which promotes double-stranded cleavage. The authors suggest that **LEI-800** represents an interesting chemotype for the development of effective drugs that can overcome growing bacterial resistance to fluoroquinolones.

**Figure 46:**
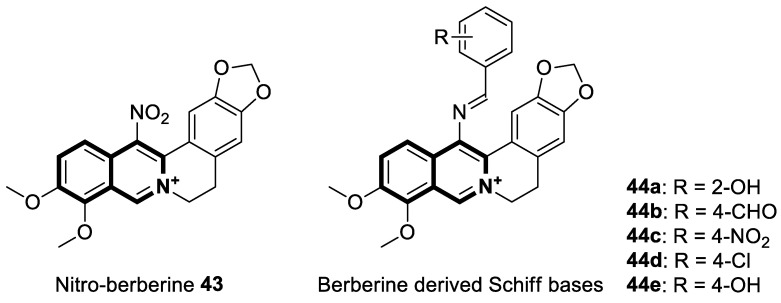
Nitro-berberine **43** and Schiff bases **44a**–**e** derived from Berberine, with antimicrobial activity.

A series of Schiff bases of plant-derived isoquinoline alkaloid Berberine were synthesized and tested on a wide range of Gram-negative and Gram-positive bacterial strains by Noghabi and collaborators [144]. The antibacterial activity of these Berberine synthetic derivative **43** analogs (**44a**–**e**) (Figure 46) was compared with their antioxidant properties. Among them, three Schiff bases, **44a**, **44b**, and **44d**, were effective and inhibited the growth of all tested microorganisms (IC_50_ values ≤ 37.74 μg/mL). The nitro-derivative of Berberine **43** turned out to be active against Gram-positive, aerotolerant strain *Streptococcus pyogenes*, whereas Schiff base **44c** displayed antimicrobial effects in the case of Gram-negative *Acinetobacter baumannii*.

Moreover, the in vitro antioxidant properties of synthesized Berberine analogs were evaluated using a colorimetric assay. In general, no correlation was found for the tested compounds in terms of antimicrobial and antioxidant activity. It should be pointed out that the presence of the electron-donating OH group at position para of the phenyl ring (compound **44e**) (Figure 46) may facilitate antioxidant activity by increasing the electron density.

#### 2.2.2. Tetrahydroisoquinoline-Based Compounds

**Figure 47:**
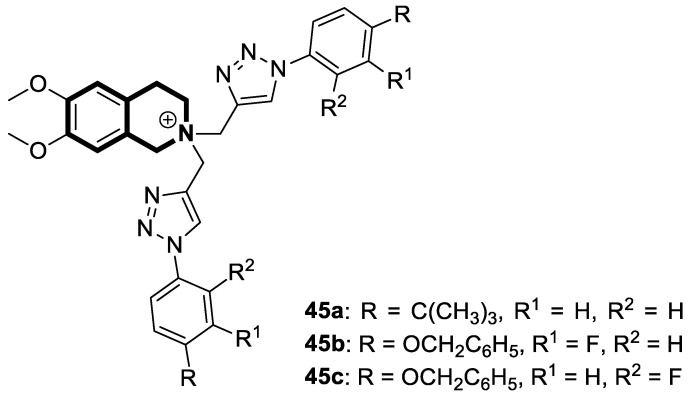
2,2-*Bis*((1-(aryl)-1*H*-1,2,3-triazol-4-yl)methyl)-6,7-dimethoxy-1,2,3,4-tetrahydroisoquinolin-2-ium derivatives **45a**–**c** as potent antibacterial agents.

In 2022, Payne et al. reported the synthesis of novel cationic 6,7-dimethoxy-1,2,3,4-tetrahydroisoquinolines bearing 1,2,3-triazole as a linker using copper(II)-catalyzed azide-alkyne cycloaddition (Figure 47) [145]. The designed compounds were tested against Gram-positive and Gram-negative bacteria, including clinical isolates sensitive to commonly used antibiotics.

From the eighteen compounds, three isoquinolines, **45a**, **45b** and **45c** (Figure 47), displaying prominent activity against *Staphylococcus aureus* and *Bacillus subtilis*, with MIC values of 4 µg/mL, were identified. Moreover, their bactericidal activity was confirmed at the same concentration. All compounds were inactive against Gram-negative strain *Escherichia coli* at a concentration of 128 µg/mL. The most prominent compound **45a** exerted a broad potency and was proven to eliminate *Mycobacerium tuberculosis* strain H37Rv with an MIC of 6 μg/mL. It may be concluded that the introduction of hydrophobic groups such as *tert*-butyl (the most active compound **45a**), naphthalene, biphenyl, or (benzyloxy)fluorophenyl substituent (compounds **45b** and **45c**) resulted in an increased activity against *Staphylococcus aureus* and *Bacillus subtilis*.

In further biological tests supported through phenotypic investigation using *Bacillus subtilis* as a model organism, it was found that compound **45a** may target cell wall or membrane homeostasis. It was also demonstrated that **45a** eliminated *Staphylococcus aureus* effectively, with no resistance observed after thirty days of sequential passage, even at sub-inhibitory concentrations. In light of these results, compound **45a** and its analogs may be potential candidates for further drug development that could help tackle the problem of antibiotic resistance.

**Figure 48:**
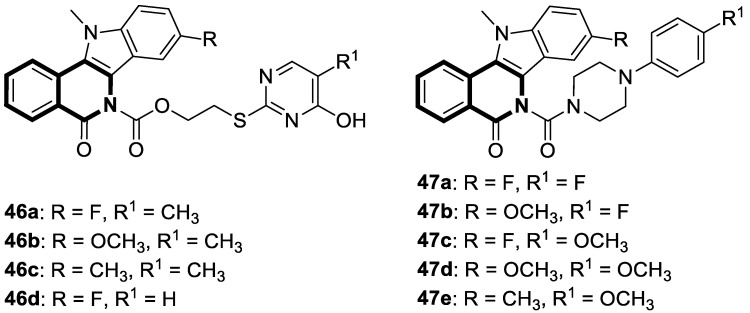
Isoquinoline (8-carboline) analogs bearing pyrimidine **46a**–**d** and piperazine **47a**–**e** cores displaying antimicrobial activity.

Using a molecular hybridization approach, Verma and co-workers have successfully synthesized a novel series of isoquinoline (8-carboline) analogs bearing pyrimidine and piperazine cores (Figure 48) [146]. In the study, the compound containing fluorine atom **46d** (R = F, R^1^ = H) showed a strong antibacterial activity against Gram-negative strains of *Escherichia coli* and *Klebsiella pneumoniae* (MICs = 1.5 µg/mL vs. Ciprofloxacin: MICs = 1.5 µg/mL), while the compounds of alkyl and alkoxyl substituents **46b** (R = OCH_3_, R^1^ = CH_3_), **47d** (R = OCH_3_, R^1^ = OCH_3_), and **47e** (R = CH_3_, R^1^ = OCH_3_) displayed antibacterial activity against Gram-positive bacteria (*Staphylococcus aureus* and *Bacillus subtillis*: MICs = 1.5 µg/mL vs. Ciprofloxacin: MICs = 3.12 µg/mL and 1.5 µg/mL). Antifungal properties against *Aspergillus niger*, *Aspergillus oryzae*, *Candida albicans*, and *Penicillium chrysogenum* have been demonstrated for compounds **46a** (R = F, R^1^ = CH_3_) and **47a** (R = F, R^1^ = F). Their calculated MICs (1.5 µg/mL) were comparable to reference Fluconazole (1.5–3.12 µg/mL). Furthermore, compound **46c** (R = CH_3_, R^1^ = CH_3_) showed broad antioxidant properties as a radical scavenger and iron chelating agent. Interestingly, in tests against *Mycobacterium tuberculosis* (H37Rv strain), piperazine-containing derivatives **47b** and **47c** showed good anti-tuberculosis activity. The authors suggest that the synthesized compounds may serve as preliminary leading structures for designing more potent multitarget chemotherapeutic agents.

### 2.3. Anti-Mycobacterial Agents

Globally, *Mycobacterium tuberculosis* (Mtb) poses a serious health concern to humans, which has led to the development of new anti-mycobacterial agents. This pathogen causes a contagious infectious disease known as tuberculosis (TB) [147]. In terms of mortality, the disorder has surpassed HIV/AIDS. It may primarily affect the lungs (pulmonary TB), but also other body parts (extrapulmonary TB). Recently, nearly 1.5 million deaths have been associated with this infectious disease, which affects about one-third of the world’s population. In the current generation of tuberculosis therapy, short-term regimens are effective in treating sensitive strains of Mtb. It is also becoming increasingly common in many countries for tuberculosis to be caused by multidrug-resistant (MDR) and extensively drug-resistant (XDR) strains of *Mycobacterium tuberculosis* [148]. As soon as the quinoline derivative Bedaquiline [149] (known under the brand name Sirturo) was used clinically for the treatment of MDR-TB, drug-resistant strains emerged, resulting in the rapid loss of its activity. Therefore, to combat multidrug-resistant and extensively drug-resistant tuberculosis successfully, new anti-tuberculosis molecules must continually be discovered [150].

#### 2.3.1. Isoquinoline-Based Compounds

**Figure 49:**
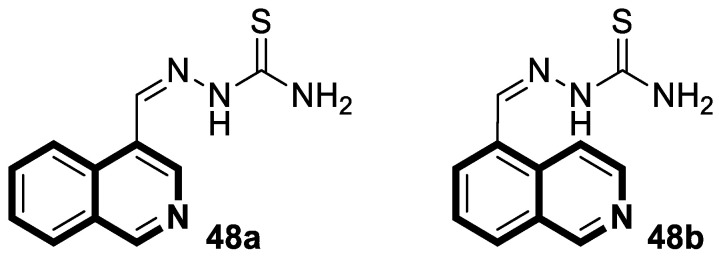
Isoquinolines **48a** and **48b**, with anti-*Mycobacterium tuberculosis* activity.

By condensing thiosemicarbazide with isoquinoline-4-carbaldehyde or isoquinoline-5-carbaldehyde in methanol or ethanol under mild conditions, Chun-Xiu Liu and co-workers have obtained 2-(isoquinolin-4-ylmethylene)hydrazinecarbothioamide (**48a**) and 2-(isoquinolin-5-ylmethylene)hydrazinecarbothioamide (**48b**) (Figure 49) [151].

An in vitro assay revealed that isoquinoline derivative **48a** had a moderate anti-mycobacterial potency (MIC = 4 μg/mL), whereas its constitutive isomer **48b** displayed a relatively weak anti-Mtb activity, with an MIC value of 8 μg/mL. The positive control in the tests was Isoniazid, which achieved an MIC value of 0.25 µg/mL. Calculated for both compounds’ ClogP values, the slightly higher activity of compound **48a** might be the result of its greater lipophilicity (ClogP of **48a** = 2.26 vs. ClogP of **48b** = 2.08). In light of these results, it seems clear that more lipophilic heterocyclic compounds might be more effective as anti-Mtb agents.

#### 2.3.2. Tetrahydroisoquinoline-Based Compounds

**Figure 50:**
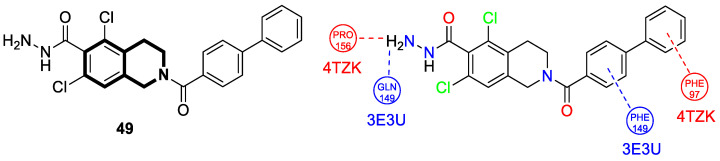
Designed 5,7-di-Cl-THIQ derivative **49** with anti-mycobacterial activity, and docked pose images of designed 5,7-di-Cl-THIQ derivative **49** with enoyl reductase 4TZK (marked in red) and peptide deformylase 3E3U (marked in blue).

In 2023, Kumar et al. reported novel hydrazides based on a 5,7-dichloro-1,2,3,4-tetrahydroisoquinoline scaffold. Apart from the cytotoxic effect of the most potent compounds, the authors also studied anti-mycobacterial activity against a luminescent strain of *Mycobacterium tuberculosis* (Mtb, H37ralux lab strain) [152]. In order to determine the antitubercular properties of the tested compounds, a luminescence reduction in the exposed H37Ralux strain was determined. The synthesized 5,7-di-Cl-THIQ derivative—bearing a biphenyl group—**49** (Figure 50) proved to be most active, with an IC_50_ of 4.7 μM.

**Figure 51:**
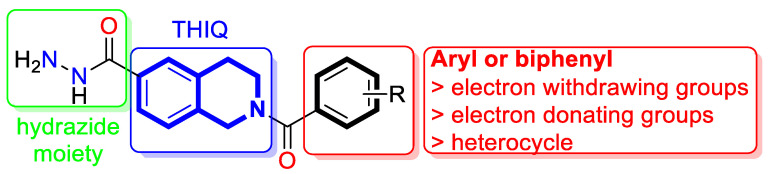
SAR summary of the designed THIQ-hydrazide hybrids.

For the purpose of determining the putative binding position of the designed ligand **49** at the active sites of *Mycobacterium tuberculosis* enoyl reductase (4TZK) and peptide deformylase (3E3U), a molecular docking study of the compound was performed. Based on the computed in silico results for compound **49** and *Mycobacterium tuberculosis* enoyl reductase (INHA) 4TZK, the ligand formed one hydrogen bond with proline 156 and one π-π stacking with phenylalanine 97, respectively (Figure 50). In the active site, hydrogen bonds were formed between the NH_2_ group of the hydrazide moiety and proline 156. The 4′-phenyl substituent participated in an additional hydrogen bond with phenylalanine 97. Similarly, in the case of *Mycobacterium tuberculosis* peptide deformylase 3E3U, the docked compound **49** formed a hydrogen bond via the hydrazide NH_2_ group and Gln149. The phenyl ring took part in a π-π stacking interaction with phenylalanine 149. The authors concluded that the essential source of the anti-mycobacterial activity of the designed compounds is present in their structure: the hydrazide moiety, which is critical for forming strong hydrogen bonds with the target proteins, tetrahydroisoquinoline core, and aryl, heterocyclic, or biphenyl groups, containing electron-withdrawing substituents (Figure 51).

### 2.4. Antifungal Agents

In recent years, there have been an increasing number of fungal species emerging and multiplying that are resistant to antifungal agents, presenting a particular problem for public health. A number of fungi are prevalent in our environment, and individuals who have weakened immune systems are particularly at risk from several fungal strains. Among the phytopathogenic fungi, *Aspergillus*, *Penicillium*, *Alternaria*, and *Fusarium* are the most prominent ones that are commonly found in the soil and in the air. Some species of these genera can cause decay during food storage, plant diseases, and even invasive diseases in animals. Food crops are susceptible to phytopathogenic fungi that colonize them and produce mycotoxins, which pose a significant risk to humans and animals if they accumulate in an edible part. Plant-derived food products contaminated with fungi and mycotoxins can cause many adverse conditions called mycotoxicosis. These compounds are also known as allergens and carcinogens, and are linked, in particular, to hepatocarcinoma [153,154,155].

#### 2.4.1. Isoquinoline-Based Compounds

**Figure 52:**
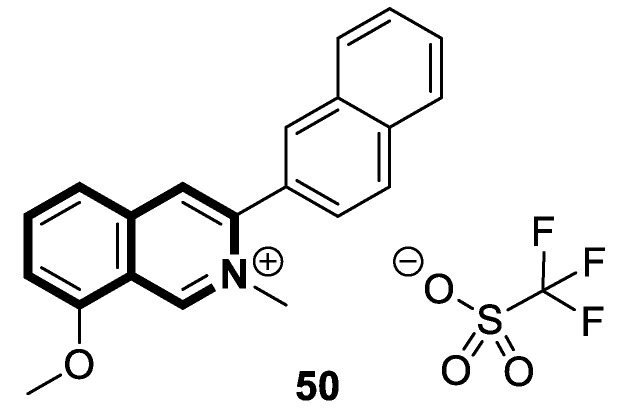
3-Arylisoquinoline derivative **50** with antifungal activity.

Based on the structures of natural isoquinoline alkaloids—Sanguinarine, Chelerythrine, and Berberine—novel quaternized isoquinoline derivatives were obtained through a two-step synthetic procedure involving [4+2] cyclization and Suzuki coupling [156]. The designed compounds showed in vitro broad-spectrum and higher than Sanguinarine antifungal activity at a concentration of 50 mg/L. Particularly, compound **50** (Figure 52) was found to be effective against plant pathogens such as *Alternaria solani*, *Alternaria alternata*, and *Physalospora piricola*. Moreover, the estimated in vivo EC_50_ of **50** (3.65 mg/L) was comparable with 2,4,5,6-tetrachlorobenzene-1,3-dicarbonitrile (Chlorothalonil, 3.87 mg/L). According to the molecular electrostatic map and docking analysis, compound **50** was covered by a positive potential, which made the interaction with succinate dehydrogenase’s negative amino acid residues optimal.

#### 2.4.2. Tetrahydroisoquinoline-Based Compounds

**Figure 53:**
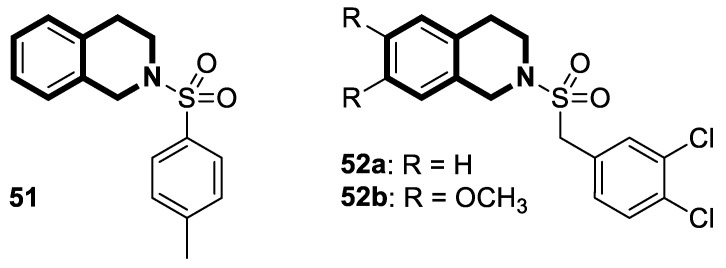
Antifungal *N*-sulfonyl-1,2,3,4-tetrahydroisoquinolines **51**, **52a**, and **52b**.

The study carried out by A. Fernández Baldo et al. revealed that the obtained *N*-sulfonyl-1,2,3,4-tetrahydroisoquinoline (NSTHIQ) derivatives **51**, **52a**, and **52b** (Figure 53) display significant antifungal properties against various fungal species [157].

Tetrahydroisoquinolines were synthesized under mild conditions using an environmentally friendly Preyssler heteropolyacid catalyst. It is worth noting that compound **51** inhibits *Botrytis cinerea* growth by 73.3%. Compound **52a** demonstrated a significant antifungal efficacy against *Aspergillus flavus* and *Aspergillus parasiticus* (inhibition growth by 77.8% and 94.1%, respectively). Moreover, it was found that compound **52a** inhibited the growth of *Penicillum expansum* (inhibition of growth by 73.2%) and *Penicillium verrucosum* (inhibition of growth by 80.9%). Additionally, compound **52a** exhibited moderate antifungal properties against *Botrytis cinerea* (inhibition of growth by 66.7%). Also worth mentioning is compound **52b**, which, in tests, showed antifungal activity against *Aspergillus niger*, *Aspergillus flavus*, *Aspergillus parasiticus*, and *Aspergillus ochraceus*, with an inhibition of growth by 71.4%, 63.9%, 73.5%, and 82.1%, respectively.

**Figure 54:**
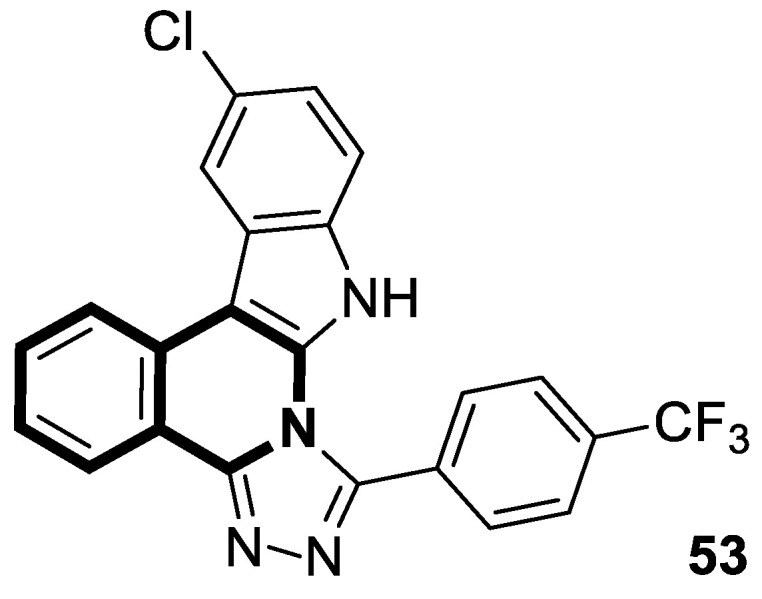
Identified 9-chloro-1-(4-(trifluoromethyl)phenyl)-12*H*-indolo[2,3-*c*][1,2,4]triazolo[3,4-*a*]isoquinoline (**53**) with antifungal and antimicrobial properties.

Basavarajaiah and colleagues identified a series of novel 9-chloro-1-(4-substituted-phenyl)-12*H*-indolo[2,3-*c*][1,2,4]triazolo[3,4-*a*]isoquinolines (**53**), depicted in Figure 54 [158].

An in vitro assay determined the lowest concentration of the tested compounds that is required to inhibit the visible growth of microorganisms. The calculated MIC values for *Staphylococcus aureus*, *Bacillus subtilis*, *Escherichia coli* (1.56 μg/mL), *Cadida albicans*, *Fusarium oxysporum*, and *Aspergillus flavus* (3.125 μg/mL) were compared to the standard drugs Ciprofloxacin (MICs = 3.125 μg/mL) and Itraconazole (MICs = 6.25 μg/mL). In a DNA cleavage assay using plasmid pBR322 DNA, compound **53** was found to fully cut supercoiled DNA.

**Figure 55:**
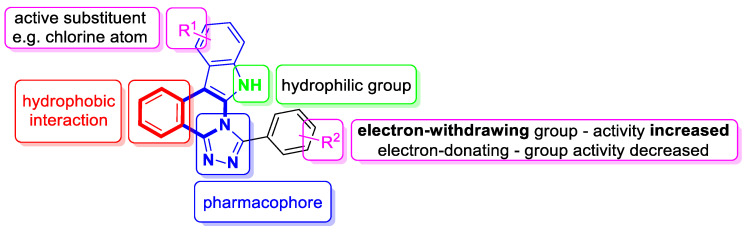
SAR for synthesized 12*H*-indolo[2,3-*c*][1,2,4]triazolo[3,4-*a*]isoquinoline derivatives.

The structure–activity analysis indicates that 1,2,4-triazole and indole systems constitute a significant element of the pharmacophore of the designed molecules. Furthermore, the biological activity can be significantly enhanced by the presence of halogen atoms (e.g., a chlorine atom R^1^) or strongly electron-withdrawing groups (e.g., a trifluoromethyl substituent R^2^). In turn, the aromatic core of the isoquinoline skeleton might be responsible for good hydrophobic interactions. Moreover, beneficial for their activity is also a group that enhances the hydrophilicity of molecules (e.g., NH) (Figure 55).

### 2.5. Antiviral Agents

According to the World Health Organization (WHO) data, there are over 4 million deaths a year associated with viral infections in the human population. Particularly, chronic infections such as hepatitis B or hepatitis C have been a global health problem. The estimation shows that there are more than 250 million HBV carriers and approximately 50 million people infected with HCV [159,160]. Therefore, a major part of the worldwide population is at risk of acute or chronic liver disease. Other problems are influenza A virus (IAV) and respiratory syncytial virus (RSV), belonging to widespread human pathogens, and Human Immunodeficiency Virus, whose infections have reached over 40 million people. As a result of the COVID-19 pandemic caused by SARS-CoV-2, almost 700 million humans have been infected, and the mortality rate is approximately 1% [161,162].

#### 2.5.1. Isoquinoline-Based Compounds

Isoquinoline derivatives are claimed to be particularly useful in the treatment and prophylaxis of Human Immunodeficiency Virus Type 1 (HIV-1) infections, especially as HIV-1 integrase inhibitors, HIV-1 integrase and reverse transcriptase dual inhibitors, HIV-1 transcription inhibitors, or HIV-1 Tat-TAR interaction inhibitors [163].

**Figure 56:**
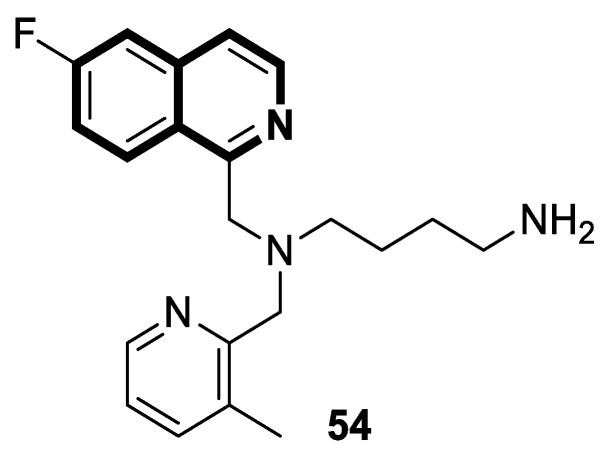
Anti-HIV isoquinoline-based derivative **54**.

A series of 1-methylene-isoquinolines developed by Shad and co-workers were found to be CXCR4 chemokine receptor antagonists, displaying encouraging results in anti-HIV assays [164]. In particular, *N*^1^-((6-fluoroisoquinolin-1-yl)methyl)-*N*^1^-((3-methylpyridin-2-yl)methyl)butane-1,4-diamine (**54**), depicted in Figure 56, prepared from the Boc-protected precursor, was the most promising candidate for the further development of antiviral agents. The isoquinoline-based derivative **54** competed with CXCL12 for binding to CXCR4, as evidenced by its nanomolar IC_50_ value (0.64 nM). However, compound **54** displayed a diminished potency in the calcium-induced flux assay, and its concentration needed to inhibit CXCL12-induced calcium signaling by 50% was approximately 10 times higher (IC_50_ = 6.2 nM). It has been discovered that compound **54** has an excellent antiviral activity against HIV-1 (EC_50_ = 6.2 nM) and HIV-2 (EC_50_ = 3.8 nM), showing no cytotoxicity against human T-cells MT4, which are highly susceptible to HIV infection (concentration required to reduce viability by 50%—CC_50_ = 30.77 µM).

#### 2.5.2. Dihydroisoquinoline-Based Compounds

Every year, more than a million new cases of chronic hepatitis B are reported worldwide, affecting a global population of 250 million people. Cirrhosis and hepatocellular carcinoma are the leading causes of death related to HBV infection. Standard medical armament against chronic hepatitis B is limited to the pegylated form of interferon-α (PEG-IFN-α) and nucleotide-based antiviral agents that inhibit viral replication [165,166]. Due to the serious side effects associated with each of these agents, effective small-molecule inhibitors of hepatitis B virus replication are needed. These compounds, known as capsid assembly modulators (CAMs), interfere with capsid assembly and the encapsidation of pregenomic RNA (pgRNA) and hepatitis B viral polymerase (P).

**Figure 57:**
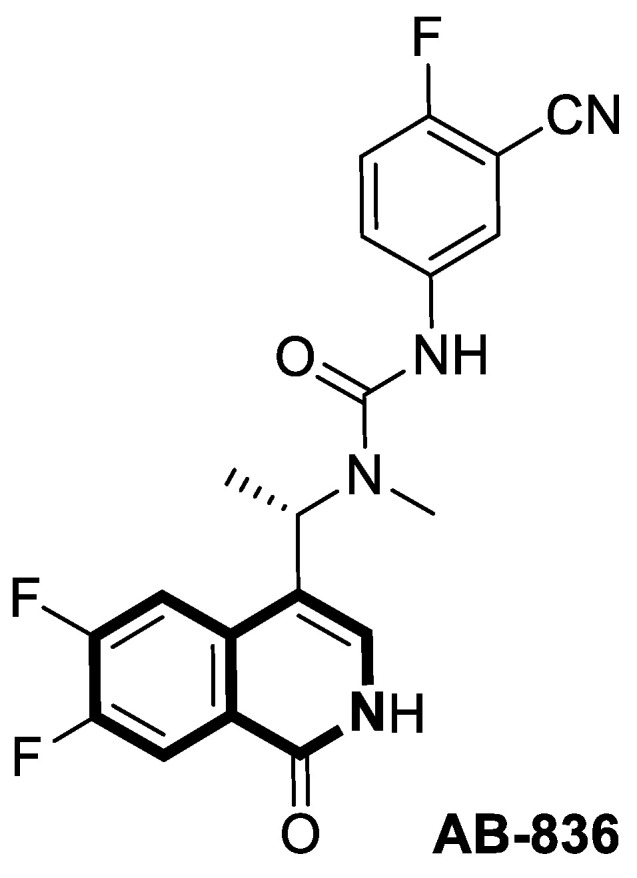
Isoquinoline-based clinical candidate, **AB-836**—HBV capsid assembly modulator.

Cole et al. identified a novel isoquinoline-based clinical candidate, (*R*)-3-(3-cyano-4-fluorophenyl)-1-(1-(6,7-difluoro-1-oxo-1,2-dihydroisoquinolin-4-yl)ethyl)-1-methylurea (**AB-836**), in a series of HBV capsid assembly modulators (Figure 57) [167]. This compound was selected for further profiling based on its combination of antiviral potency, ADME physicochemical properties, and good aqueous solubility.

In the hydrodynamic HBV transfection mouse model, **AB-836** demonstrated an increased oral efficacy. It was also observed that this compound showed good pharmacokinetics in rats and monkeys in addition to good oral exposure in mice and low-dose efficacy in an in vivo model based on daily dosing. The biochemical analysis of viral replication intermediates allowed the authors to determine the compound’s mode of action. By incubating at a high concentration in human hepatoblastoma cells (HepDE19), pgRNA, core protein, and capsid particle formation were maintained, and rcDNA and encapsidated pgRNA were inhibited. As a result, the observed profile represents a CAM-E-mediated mechanism of action, specifically maintaining capsid formation while inhibiting pgRNA encapsidation. In addition to excellent ADME properties, this compound was also highly aqueous-soluble and demonstrated suitable pharmacokinetics. Importantly, the compound **AB-836** as an advanced drug candidate has been shown to be non-mutagenic in an AMES fluctuation test up to 100 μM and possesses a clean CYP profile. In addition, this derivative entered a first-in-human trial development and, throughout 10 days, was well tolerated at single ascending dose (SAD) and multiple ascending dosage levels (MAD).

#### 2.5.3. Tetrahydroisoquinoline-Based Compounds

Additionally, it has been observed that human-transmitted viruses can spill over to wild animals and that animals can transmit viruses to humans. Due to this, it is expected that SARS-CoV-2 will persist for a long time. COVID-19 is being treated with small-molecule drugs, including an adenosine nucleoside triphosphate analog, Remdesivir, reversible covalent SARS-CoV-2 main protease inhibitor, Paxlovid, and synthetic ribonucleoside analog of cytidine, Molnupiravir, but additional alternatives will be needed as SARS-CoV-2 variants emerge continuously, especially those with drug resistance [168,169,170,171].

**Figure 58:**
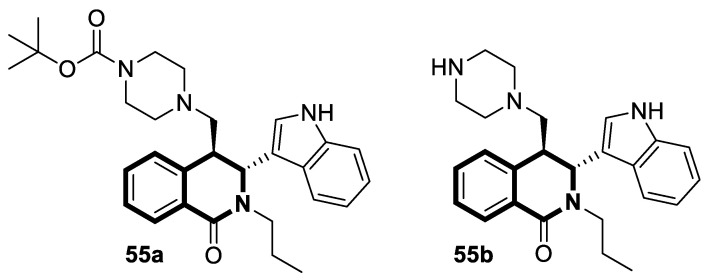
Novel compounds based on 1,2,3,4-tetrahydroisoquinoline scaffold **55a** and **55b** with anti-SARS-CoV-2 activity.

In 2023, Wang and co-workers described the synthesis of two novel heterocyclic compounds based on 1,2,3,4-tetrahydroisoquinolinone cores **55a** and **55b**, depicted in Figure 58 [172]. In preliminary studies, it was proven that these compounds inhibited SARS-CoV-2 replication in a cell line derived from an African green monkey kidney (Vero E6).

Compound **55a** exhibited a higher anti-SARS-CoV-2 activity than **55b**, with an EC_50_ of 3.15 μg/mL and SI exceeding 63.49. It was found that **55a**, with a Boc-protective group, inhibited SARS-CoV-2 replication in human lung cells Calu-3 under 10 μM (EC_50_ = 2.78 μM, SI: >71.94). The estimated EC_50_ value for chloroquine was EC_50_ = 44.90 μM. To elucidate the mechanism of inhibition of viral replication, a time-of-addition assay was performed.

Compared with Chloroquine in Vero E6 and lung adenocarcinoma Calu-3 cells, **55a** inhibits post-entry viral replication primarily. The authors suggest that compound **55a** is a promising drug candidate and, in comparison to Chloroquine, possesses a different, unique mechanism of antiviral action. It was observed that, for *tert*-butyl 4-(((3R,4S)-3-(1*H*-indol-3-yl)-1-oxo-2-propyl-1,2,3,4-tetrahydroisoquinolin-4-yl)methyl)piperazine-1-carboxylate-targeting host cells, the pH value in the endosome was not increased. Viral entry demands lower values of endosomal pH. In the case of Chloroquine, an elevated pH of the endosome, which inhibits SARS-CoV-2 entry into host cells, is observed.

**Figure 59:**
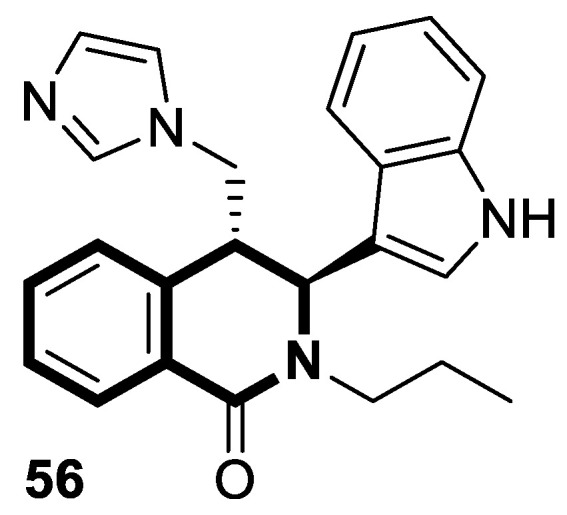
Anti-coronavirus synthetic 3,4-dihydroisoquinolin-1(2*H*)-one derivative **56**.

Kandinska and colleagues described anti-coronavirus preliminary studies of previously reported trisubstituted 3,4-dihydroisoquinolin-1(2*H*)-ones bearing different heteroaryl cores [173]. Considering the extensive work that has been carried out on antiviral investigations, the authors claimed that (3S)-4-((1*H*-imidazol-1-yl)methyl)-3-(1*H*-indol-3-yl)-2-propyl-3,4-dihydroisoquinolin-1(2*H*)-one **56** (Figure 59) synthesized by Burdzhiev in 2019 inhibited coronavirus (strains OC-43 and 229E) in vitro replication (IC_50_ = 0.5 μM for 229E and IC_50_ = 1 μM for OC-43) [174]. The prominent antiviral activity of compound **56** was also accompanied by its influence on the viral absorption stage (229E) after 60 min of exposure to healthy human fibroblasts (MRC-5 cell line).

There is strong evidence that influenza RNA-dependent RNA polymerase (RdRp) is a crucial protein for influenza replication, and its subunit polymerase acidic *N*-terminal (PA_N_) endonuclease was confirmed to be a potential target for anti-influenza agents. This subunit—polymerase acidic protein (PA)—in the second step of viral replication, cleaves nucleotides, producing capped RNA fragments that serve as primers for viral mRNA elongation. Therefore, PA_N_ endonuclease—which is highly conserved in all influenza A strains (IFA) and subtypes—has become an attractive antiviral drug target [175]. In recent years, much effort has been made to identify novel PA_N_ inhibitors of this widespread human pathogen. These inhibitors chemically belong to diverse classes of heterocyclic compounds or polyphenols.

**Figure 60:**
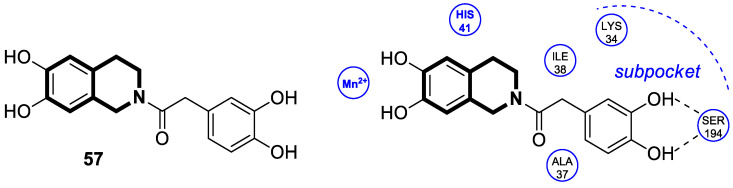
Constrained dopamine-based PA_N_ endonuclease inhibitor—1-(6,7-dihydroxy-3,4-dihydroisoquinolin-2(1*H*)-yl)-2-(3,4-dihydroxyphenyl)ethenone (**57**)—and molecular docking of compound **57** binding within PA protein.

Liao and colleagues, in 2020, found that the conformationally constrained analog of dopamine-amide bearing 1,2,3,4-tetrahydroisoquinoline-6,7-diol moiety **57** (Figure 60) displayed strong in vitro anti-influenza A virus activity (EC_50_ = 2.58 µM vs. Peramivir EC_50_ = 5.26 µM and Epigallocatechin gallate EGCG EC_50_ = 28.93 µM) [176].

Tetrahydroisoquinoline **57** in Madin–Darby canine kidney (MDCK) cells reduced the cytopathic effect (CPE), indicating its ability to protect the cells from influenza virus-induced CPE. Moreover, it was demonstrated that compound **57** exhibited a strong inhibition of endonuclease activity (EC_50_ = 489.39 nM).

Regarding that PA_N_ endonuclease is a potential drug target, compound **57** was docked into the structure of the H1N1 protein in the presence of BXA (Figure 60). It was noticed that the 1,2,3,4-tetrahydroisoquinoline-6,7-diol moiety could form hydrophobic contact with the key residue histidine 41 in endonuclease and may assist in metal (manganese) chelation. It was found that the rigid 1,2,3,4-tetrahydroisoquinoline-6,7-diol part of the molecule allowed for an orientation toward the subpocket and formed double hydrogen bonds with serine residue 194. On the other hand, the aryl ring could form a greater number of hydrophobic contacts, e.g., with lysine 34, isoleucine 38, or alanine 37.

**Figure 61:**
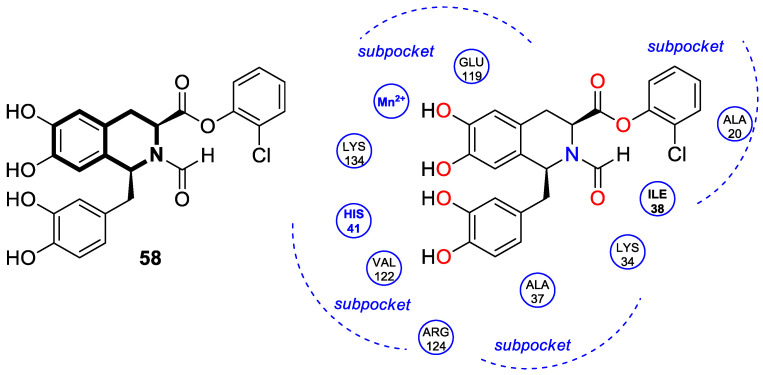
Designed novel anti-IFA 1,3-*cis*-2-substituted-1-(3,4-dihydroxybenzyl)-6,7-dihydroxy-1,2,3,4-tetrahydroisoquinoline-based derivative **58**, and molecular docking results of **58** into PAN protein.

Encouraged by these findings and based on the recently discovered chemotype of PAN endonuclease inhibitors, in 2022, Liu and co-workers developed a novel series of anti-influenza 1,3-*cis*-2-substituted-1-(3,4-dihydroxybenzyl)-6,7-dihydroxy-1,2,3,4-tetrahydroisoquinoline-3-carboxylic acid derivatives with optimal physicochemical properties [177]. The promising results of the antiviral activity of this series of compounds allowed for a more detailed structure–activity relationship analysis. As a result, the most prominent anti-IFA compound **58** was identified, as shown in Figure 61 (EC_50_ = 4.50 µM vs. Peramivir EC_50_ = 5.26 µM and cytotoxicity concentration CC_50_ > 100 µM, selectivity index SI (CC_50_/EC_50_) > 22.2 vs. Peramivir SI > 19.0). In vitro protection against influenza viral infection in A/WSN/33-virus-infected MDCK cells was confirmed.

It was found that compound **58** fits well into a subpocket of the PAN protein, with an *ortho*-chlorophenyl substituent at position 3 of the 6,7-dihydroxy-1,2,3,4-tetrahydroisoquinoline moiety where the aryl ring is bonded to the hydrophobic wall (alanine residue 20). The halogen atom makes favorable contact with isoleucine residue 38. The authors speculated that the enhanced potency could be a result of the good complementarity between designed ligand **58** and protein subpockets, as well as its additional interactions with the undeveloped pocket.

In Africa, a particular concern for public health is Ebola virus disease (EVD) caused by the Sudan virus (SUDV) and Ebola virus (EBOV), which, since 1976, has emerged and spread across the continent. There is a high mortality rate associated with EVD, making it one of the most lethal transmissible infections. Although monoclonal antibodies have been approved for targeting the EBOV-GP epitopes, the production of vaccines during an outbreak of the virus is not practical. It is essential therefore to seek small-molecule drugs with stable storage properties and suitability for oral administration and that exhibit antiviral efficacy against EBOV. There are many reports about the discovery of Ebola virus entry inhibitors that have been extensively studied and synergistic drug combinations which are effective at blocking the EBOV [178].

**Figure 62:**
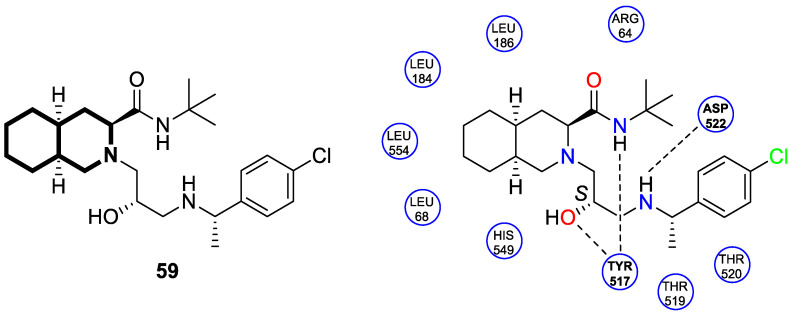
Designed Ebola virus entry inhibitor—(3*S*,4a*S*,8a*S*)-*N*-(*tert*-butyl)-2-((*S*)-3-(((*S*)-1-(4-chlorophenyl)ethyl)amino)-2-hydroxypropyl)decahydroisoquinoline-3-carboxamide (**59**)—and predicted binding mode of compound **59** with EBOV-GP.

In 2022, through the high-throughput screening of diverse libraries, Han and collaborators found a hit structure based on a decahydroisoquinoline-3-carboxamide scaffold, which upon further optimization led to a very potent Ebola entry (*S*)-hydroxylated inhibitor **59** (Figure 62). This compound was obtained from commercially available (*S*)-1,2,3,4-tetrahydroisoquinoline-3-carboxylic acid in a several-step procedure, including cyclization, aminolysis, and reduction [179].

It was found that compound **59** exerted an approximately 3 times better activity against Ebola virus glycoprotein (EBOV-GP) than the reference Toremifene, with an IC_50_ value of 0.05 μM and SI = 98. By interacting with EBOV-GP, Toremifene has the ability to destabilize the viral protein. Molecular docking and the prediction of binding modes of compound **59** to EBOV-GP may support its high inhibitory activity (Figure 62). The saturated isoquinoline ring is incorporated into a relatively flexible hydrophobic pocket composed of leucine residues 68, 184, 186, and 554. In the (*S*)-OH configuration, three hydrogen bonds are generated, two with tyrosine 517 and one aspartic acid 522, which might improve the binding with protein and enhance the inhibitory effect. A one-carbon aliphatic linker—instead of any steric, bulky groups—between the 4-chlorophenyl moiety and secondary amine group is optimal.

**Figure 63:**
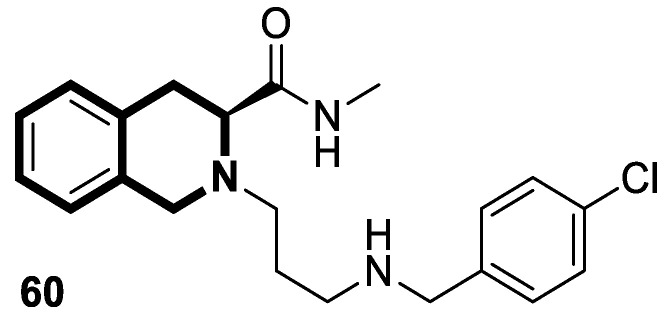
Novel Ebola virus entry 1,2,3,4-tetrahydroisoquinoline-based inhibitor **60**.

Based on the above-mentioned studies by Han on decahydroisoquinoline-3-carboxamide derivatives, in 2025, novel Ebola entry inhibitors bearing 1,2,3,4-tetrahydroisoquinoline, indoline, and 1*H*-indole scaffolds were designed and synthesized. In a study carried out by Lin and co-workers, an experimental model of the defective Ebola virus was used to confirm their antiviral activity and evaluate their cytotoxicity [180]. The most potent and easy-to-synthesize compound **60** (Figure 63) displayed an inhibitory activity compared with the previously obtained Ebola entry inhibitors and Toremifene (IC_50_ = 1.50 µM and IC_90_ = 2.01 µM vs. IC_50_ = 0.57 µM and IC_90_ = 2.86 µM). Compound **60** also demonstrated a marked reduction in toxicity towards human embryonic kidney cells (HEK293, CC_50_ = 64.22 µM). The estimated selectivity index (SI = CC_50_/IC_50_) of 42.81 was comparable to the reference drug (SI = 39.52). The designed (*S*)-2-(3-((4-chlorobenzyl)amino)propyl)-*N*-methyl-1,2,3,4-tetrahydroisoquinoline-3-carboxamide (**60**) in the future may be a promising anti-Ebola drug.

### 2.6. Antimalarial Agents

Malaria (*paludismus*) is a recurring disease caused by protozoan single-celled parasites of the *Plasmodium* genus (the malaria parasite), which are transmitted by female *Anopheles* mosquitoes. After being bitten by an infected mosquito, the parasites enter the human body and multiply in red blood cells, leading to their lysis. This process leads to the shedding of the parasite’s progeny, merozoids, into the plasma, during which attacks of fever and chills are observed. The disease occurs primarily in Africa, Southeast Asia, and the Mediterranean region. The global incidence of malaria remains significant, and, in areas where it occurs, resistance to the drugs used is often observed [181,182,183]. Among them, there are many aromatic heterocyclic compounds, which include quinoline derivatives, i.e., quinine, chloroquine, hydroxychloroquine, or mefloquine, and pyrimidines—pyrimethamine.

#### 2.6.1. Isoquinoline-Based Compounds

**Figure 64:**
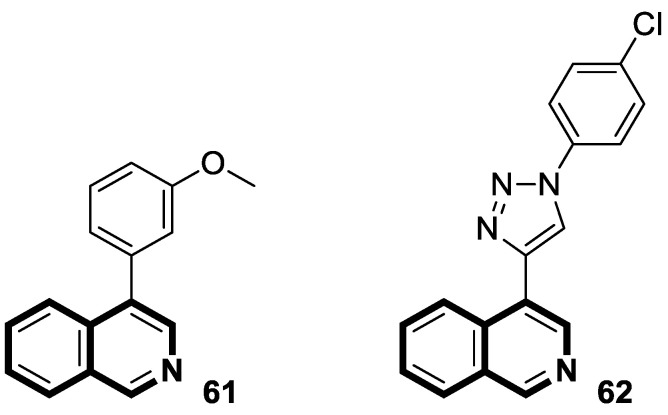
Isoquinolines **61** and **62** with anti-malarial activity.

In 2020, Sewan Theeramunkong and colleagues developed two series of novel isoquinolines containing 4-phenyl and 1,2,3-triazole rings [184]. In vitro, these compounds demonstrated differential inhibitory activity against the reference chloroquine-sensitive (3D7) and drug-resistant (K1) strains of *Plasmodium falciparum*. The determined IC_50_ values ranged from 2.31 to 364.93 µM (for 3D7) and 1.91 to 1104.51 µM (for K1). Depicted in Figure 64, 4-(3-methoxyphenyl)isoquinoline (**61**) proved to be the most promising compound that achieved a moderate efficacy against both strains. The determined IC_50_ values for 3D7 and K1 were 2.31 µM and 1.91 µM, respectively. In turn, 4-(1-(4-chlorophenyl)-1*H*-1,2,3-triazol-4-yl)isoquinoline (**62**) demonstrated the highest activity against strain K1 (IC_50_ 4.55 µM) (Figure 64). Chloroquine, quinine, mefloquine, and dihydroartemisinin were used as reference drugs in the study. The obtained IC_50_ values were in the following ranges: 0.00456–0.098 µM (for *Plasmodium falciparum* 3D7 strain) and 0.00216–0.161 µM (for *Plasmodium falciparum* K1 strain).

**Figure 65:**
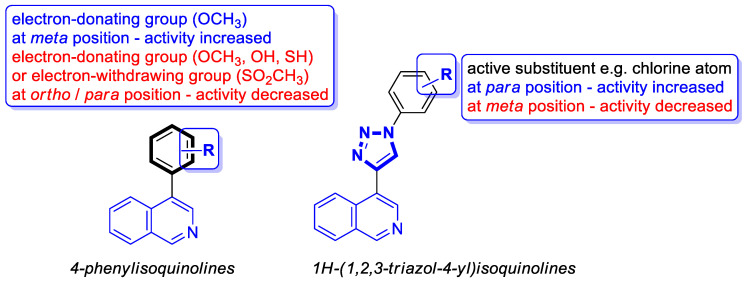
SAR for a series of phenylisoquinolines and triazole-bearing isoquinoline derivatives with anti-malarial activity.

The structure–biological activity analysis conducted for a series of isoquinolines (Figure 65) indicates that, in the group of phenyl derivatives, substitution with one electron-donating group -OCH_3_ significantly enhances antimalarial activity. In turn, both electron-donating (OCH_3_, OH, SH) and electron-withdrawing (SO_2_CH_3_) substituents in the *ortho*/*para* position limit the biological activity of the designed compounds. It could be indicated that the steric effects, rather than electronic properties of the substituents on the phenyl ring, may contribute to the anti-malarial activity of 4-phenylisoquinolines. Similarly, in a series of triazole isoquinoline derivatives, the appropriate positioning of the substituents on the phenyl ring was crucial (Figure 65). The presence of a chlorine atom in the *para* position was desirable due to its high antimalarial activity. Significantly lower in vitro activity was observed for the derivative in which the halogen atom remained in the *meta* position. Moreover, it was found that the isoquinoline ring is essential for maintaining the biological activity of derivatives. Replacing the heteroarylamine system with an isosteric naphthalene ring resulted in a decrease in antimalarial activity. It was also noted that the introduction of a 7-chloroquinoline ring, which is present in the structure of the reference drug chloroquine, into the proposed compounds also reduced its efficacy. This highlights the importance of the isoquinoline ring in the potency of this class of compounds.

**Figure 66:**
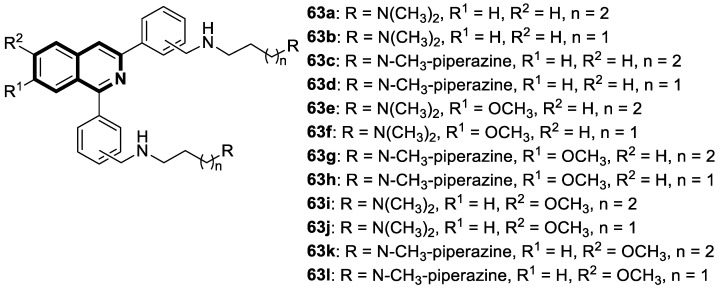
Novel 1,3-bis[(substituted-aminomethyl)phenyl]isoquinolines **63a**–**l** with antiprotozoal activity.

Guillon and colleagues have prepared a series of novel aza-heterocyclic compounds—1,3-bis[(substituted-aminomethyl)phenyl]isoquinolines **63a**–**l** (Figure 66)—which were evaluated in vitro against medically important protozoan strains: *Plasmodium falciparum* CQ-sensitive (3D7) strain, *Plasmodium falciparum* CQ-resistant (W2) strain, *Leishmania donovani*, and *Trypanosoma brucei* [185]. Additionally, human liver cancer cells (hepatocellular carcinoma-G2, HepG2) were used to assess the in vitro cytotoxicity of these compounds (IC_50_ values in the range of 2.01 µM to 14.85 µM), and the authors calculated their index of selectivity—the ratio of cytotoxic to antiparasitic activity (SI = CC_50_/IC_50_).

It was found that non-R^1^ or R^2^-substituted isoquinolines **63a**–**d** (Figure 66) were the more potent compounds in this series against the 3D7 strain compared with their methoxylated analogs (compounds **63e**–**l**). The exception was compound **63d**, for which the IC_50_ value was 5.77 µM. The introduction of a methoxy group in the isoquinoline ring appeared to be unprofitable—mainly at position 6 (compounds **63i**–**l**), for which the IC_50_ was found between 7.21 and 9.66 µM (against the 3D7 strain). The isoquinoline bearing methylpiperazinylpropylamino side chains, **63h**, was identified as the most potent antimalarial candidate, with an IC_50_ = 1.81 µM against the *Plasmodium falciparum* CQ-sensitive strain 3D7 and IC_50_ = 1.28 µM against the *Plasmodium falciparum* CQ-resistant strain.

As a result of the substitution of dimethylaminoalkyl chains on position 4 of the benzyl moieties, isoquinolines **63a** and **63b** demonstrated a better antitrypanosomal activity, with IC_50_ values of 0.64 µM and 0.35 µM compared with their *N*-methylpiperazinealkyl analogs **63c** and **63d**, whose IC_50_ values were 1.10 µM and 1.28 µM, respectively. An interesting observation was also made with their 6- and 7-OCH_3_-substituted isoquinoline analogs (compounds **63e**–**h** and **63i**–**l**), where the dimethylamino terminal amine on the polyaminoalkyl chains was substituted with an *N*-CH_3_-piperazine group, resulting in a decrease in the anti-trypanosomal activity. The calculated IC_50_ values for derivatives **63e** and **63f** were 0.52–0.55 μM vs. 0.81–1.00 μM, achieved for **63g** and **63h**, and 0.59–0.64 μM for **63i** and **63j** vs. 0.99–1.34 μM for **63k** and **63l**.

#### 2.6.2. Tetrahydroisoquinoline-Based Compounds

**Figure 67:**
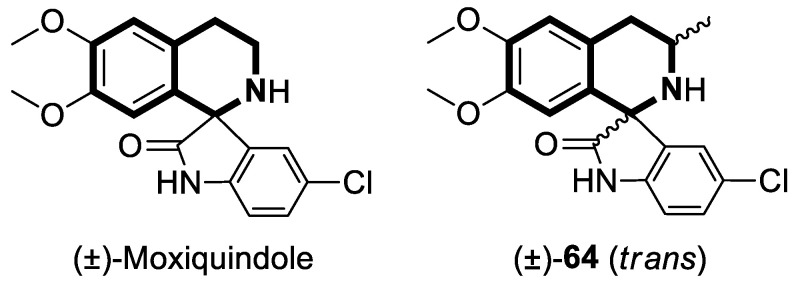
A hit compound—(±)-Moxiquindole—displaying antimalarial activity, and its homolog—(±)-5-chloro-6′,7′-dimethoxy-3′-methyl-3′,4′-dihydro-2′*H*-spiro[indoline-3,1′-isoquinolin]-2-one ((±)-**64**-*trans*).

It is worth noting that spirofused tetrahydroisoquiniline-oxindoles demonstrate a strong antiplasmodial activity with multiple modes of action. This class of heterocyclic compounds is closely related to well-known spiroindolones or spirotetrahydro-beta-carbolines, which were claimed to effectively kill all blood stages of *Plasmodium falciparum* and *Plasmodium vivax* clinical isolates in the nanomolar concentration range. There is evidence that spiroindolones inhibit the sodium efflux pump (Pf ATP4) of *Plasmodium falciparum*, a key antimalarial target, and have deleterious effects on all phases of the parasite life cycle, inhibit transmission, and act in vivo [186]. Novel compounds—3′,5′-dihydro-2′*H*-spiro[indoline-3,1′-isoquinolin]-2-ones (DSIIQs)—were designed as molecular hybrids of two privileged scaffolds—tetrahydroisoquinoline (THIQ) and oxindole (OX) [187]. In preliminary screening, these hybrids also demonstrated a promising antiproliferative activity [188]. The authors identified a hit compound—(±)-Moxiquindole, shown in Figure 67—that exerted micromolar activity against a chloroquine-sensitive (CQ-sensitive) *Plasmodium falciparum* strain 3D7 (50% inhibitory concentration IC_50_ = 1.87 μM), as well as multidrug-resistant strain *Plasmodium falciparum strain* Dd2 (IC_50_ = 1.73 μM) [189]. A study shows that (±)-Moxiquindole inhibits hemoglobin metabolism, disrupts vacuolar lipid dynamics, and causes abnormal vacuolation in parasites. This attractive pharmacological profile makes this compound a potent rapid-acting multi-stage antimalarial agent.

Further modification of the (±)-Moxiquindole structure was undertaken to obtain more potent analogs. In the oxindole fragment, changes were carried out at positions C-5 and C-6, while, at the THIQ core, modifications were performed at N-2 and C-3. The most significant increase in activity was due to the introduction of a methyl group at the C-3 position of THIQ. As a result, the designed compound **64** (Figure 67) demonstrated low-nanomolar antiplasmodial properties. Its biological activity was approximately 90 times stronger than the parent (±)-Moxiquindole (*Plasmodium falciparum* strain 3D7, IC_50_  =  21   nM). It may be concluded that the methyl group at the C-3 position of the THIQ is crucial for effective interaction with the target.

### 2.7. Anti-Trypanosoma Agents

The human African trypanosomiasis (HAT), or sleeping sickness, is another neglected disease caused by *Trypanosoma* parasites. It is almost always fatal unless treated. Humans are infected with this disease when they are bitten by infected Tsetse flies. When the brain is involved, it causes a variety of neurological disturbances, including sleep disorders, comas, and death in the end. HAT comes in a slowly progressing form due to infections with *Trypanosoma brucei gambiense* and a fast-progressing form due to infections with *Trypanosoma brucei rhodesiense*. The recently approved novel drug Fexinidazole offers an alternative to existing treatments for gambiense HAT, opening up new possibilities for managing these cases [190,191].

**Figure 68:**
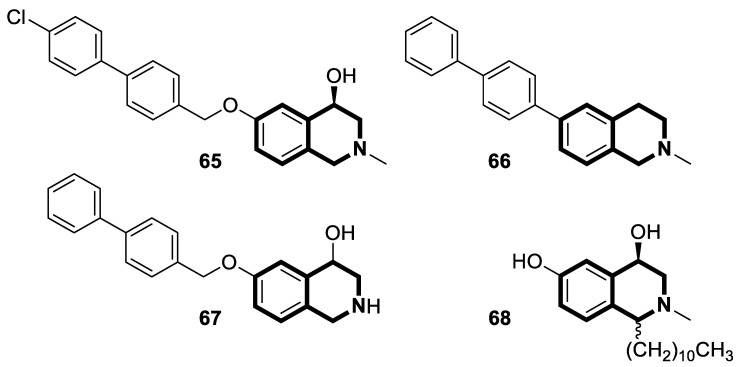
Isoquinoline derivatives **65**, **66**, **67**, and **68** with antitrypanosomal activity.

With regard to the anti-*Trypanosoma* activity of isoquinoline-based compounds, Cullen and co-workers evaluated the biological potency of a series of 2-methyl-1,2,3,4-tetrahydroisoquinoline-4,6-dioles modified at the 1, 2, 4, and 6 positions of the heterocyclic core [192]. Among them, the highest *Trypanosoma brucei rhodesiense* growth inhibitory properties were displayed in the four derivatives **65**, **66**, **67**, and **68**, shown in Figure 68.

The compounds exhibited an in vitro antitrypanosomal activity, with IC_50_ values ranging from 0.25 μM to 0.39 μM, and an optimal selectivity over mammalian Vero cells (SI > 50). Based on the calculated logD values and preliminary ADME studies, these compounds are predicted to have good absorption and metabolic stability, along with passive blood–brain barrier penetration. Consequently, these compounds offer excellent prospects for developing blood–brain barrier-permeable antitrypanosomal agents in the future.

### 2.8. Antileishmanial Agents

There is an urgent need to develop novel compounds to treat leishmaniasis, aggravated by drug toxicity and resistance, of which morbidity and mortality have recently increased. This neglected parasitic disease is transmitted by sandflies. *Leishmania* species can be categorized as either flagellated or non-flagellated based on their morphology—promastigotes that move in a fly vector (or motile form) and a non-flagellated intracellular form (amastigotes). The known types of leishmaniasis include visceral leishmaniasis (VL), dermal leishmaniasis (DL), and mucocutaneous leishmaniasis (MCL), and the most common is cutaneous leishmaniasis (CL). On the other hand, the VL form of leishmaniasis is the most severe, leading to death if untreated. A major limitation of the current compounds used against leishmaniasis is their narrow therapeutic margin and their decreased efficacy. Therefore, searching for novel therapeutic alternatives with reduced toxicity is becoming increasingly important [193,194,195,196,197].

**Figure 69:**
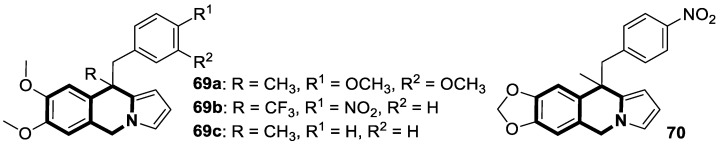
C-10-arylmethyl substituted pyrrolo[1,2-*b*]isoquinolines **69a**, **69b**, **69c**, and **70**, synthesized by Barbolla and co-workers, displaying anti-*Leishmania* activity.

In 2021, Barbolla and co-workers examined 10-benzyl-7,8-dimethoxy-10-methyl-5,10-dihydropyrrolo[1,2-*b*]isoquinoline derivatives against two selected species of *Leishmania*, i.e., *Leishmania amazonensis* and *Leishmania donovani*, which are responsible for CL and VL, respectively [198,199]. It was noticed that the introduction of a benzyl moiety was beneficial for the potency of this class of derivatives. In particular, among the tested C-10-arylmethyl-substituted pyrrolo[1,2-*b*]isoquinolines, the most active compounds against the cutaneous form of *Leishmania amazoniensis* were **69a** (IC_50_ = 3.30 μM) and **69b** (IC_50_ = 3.93 μM) (Figure 69), with high selectivity index (CC_50_/IC_50_) values of 77 and 59, respectively. These compounds appeared to be approximately 10 times more potent and selective than the reference Miltefosine (IC_50_ = 30.70 μM, SI = 4.43), followed by 10-methyl-10-(4-nitrobenzyl)-5,10-dihydro-[1,3]dioxolo[4,5-*g*]pyrrolo[1,2-*b*]isoquinoline (**70**) (Figure 69), which achieved an IC_50_ value of 8.00 μM and SI > 34. In an in vitro amastigote assay, however, the unsubstituted compound **69c** (R^1^, R^2^ = H) was generally the most active (IC_50_ = 33.6 μM, SI > 8.9).

### 2.9. Antischistosomal Agents

In the recent decade, more attention has been devoted to schistosomiasis—after malaria, the second neglected tropical disease caused by blood flukes belonging to the *Schistosoma* genus [200]. Praziquantel—(*RS*)-2-(cyclohexylcarbonyl)-1,2,3,6,7,11*b*-hexahydro-4*H*-pyrazino[2,1-*a*]isoquinolin-4-one (PZQ)—has been used for treating schistosomiasis in humans since the 1980s. Developing new therapies to combat drug resistance is necessary in order to combat this disease [201].

**Figure 70:**
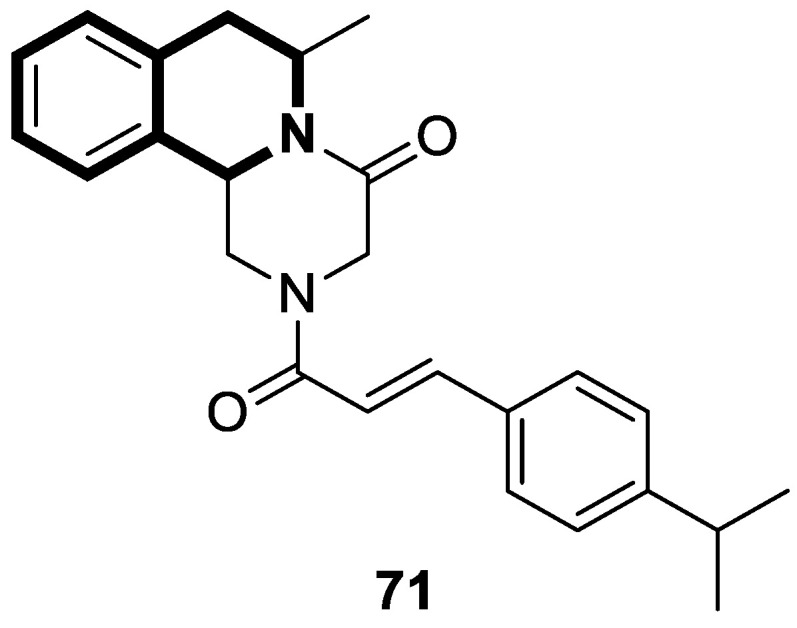
Isoquinoline derivative **71** with antischistosomal activity.

Kasago and colleagues synthesized a series of molecular hybrid compounds based on praziquantel and cinnamic acid [202]. Among the designed compounds, the PZQ-hybrid-containing isopropyl substituent at the para position on the cinnamic acid moiety **71** (Figure 70) showed a prominent in vitro activity against larval stage newly transformed schistosomula (NTS), which may actively infect humans and adult form *Schistosoma mansoni* (10 µM—78.2% activity; IC_50_ = 1.9 µM vs. IC_50_ = 0.54 µM for PZQ). As a result, (6*R*,11*bR*)-2-((*E*)-3-(4-isopropylphenyl)acryloyl)-6-methyl-2,3,6,7-tetrahydro-1*H*-pyrazino[2,1-*a*]isoquinolin-4(11*bH*)-one (**71**) proved to be effective as a reference drug. The isoquinoline derivative was identified as a potential candidate for further drug development that could help fight schistosomiasis.

## 3. Anti-Alzheimer’s Disease Activity

The multifactorial nature of Alzheimer’s disease (AD) makes it difficult to design treatments that are effective. In such complex pathology, a single-target pharmacotherapy has not been sufficient. The lack of successful and long-lasting therapies for AD motivates medicinal chemists to harness multitarget drug design. Thus, new potent anti-AD drugs should be discovered by investigating multiple targets. Also there is evidence that natural isoquinoline alkaloids can exert multitarget effects on cholinergic depletion, beta amyloid aggregation, and oxidative stress, which are involved in AD pathogenesis [203,204,205,206,207].

### 3.1. Isoquinoline-Based Compounds

**Figure 71:**
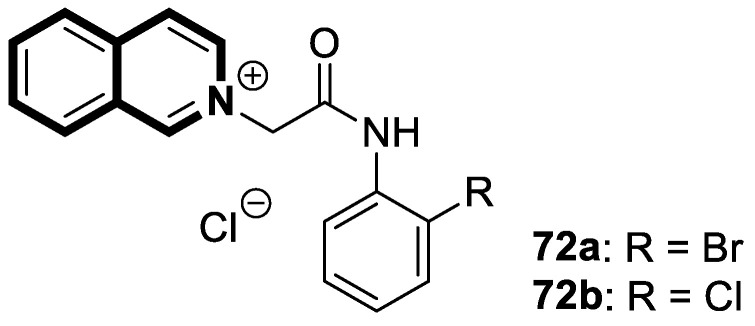
Isoquinoline-based dual inhibitors of AD-related targets.

Based on the structure of Tramiprostate—a highly specific amyloid beta (Aβ) inhibitor—Chakravarty and co-workers designed a series of heterocyclic compounds that inhibit acetylcholinesterase (AChE), butyrylcholinesterase (BuChE), and amyloid beta (Aβ) aggregation [208]. It was found that the addition of an isoquinolinium ring to Tramiprosate enhanced the binding affinity for the AChE catalytic site, as well as the exclusion of the sulfonic group. Cholinesterase inhibition assays indicated that the designed compounds achieved over 70% inhibition in both AChE and BuChE at a concentration of 100 µM. In vitro Aβ aggregation tests suggested that this class of novel anti-cholinesterase agents inhibits over 10% of Aβ aggregation at a 1 mM concentration. Among them, 2-(2-((2-bromophenyl)amino)-2-oxoethyl)isoquinolin-2-ium chloride (**72a**) (Figure 71) at a concentration of 1 mM displayed an inhibitory effect comparable to Tramiprostate on Aβ aggregation (18.07% vs. 19.68%), along with impressive AChE and BuChE inhibitory potentials (IC_50_ = 12.56 µM and IC_50_ = 12.09 µM, respectively). Compound **72a** displays the capacity to cross the blood–brain barrier. Also, there is no evidence—according to in vitro tests with the SH-SY5Y cell line—that compound **72a** could be a neurotoxic agent. The neuroprotective effects of compound **72a** in the triple transgenic mouse model of Alzheimer’s disease were recently investigated by Wei and colleagues. They found that compound **72a** weakened Aβ42-induced cytotoxicity by inhibiting glycogen synthase kinase-3 β (GSK-3 β), associated with neurological disfunctions and apoptosis. Consequently, cognitive dysfunction was effectively modulated in AD mice at an early stage of the illness as a result of these effects [209].

Comparable results against AChE and BuChE were achieved for a dual inhibitor of ChE and Aβ aggregation bearing chlorine at the position of phenyl ring **72b** (Figure 71). The compound was further extensively evaluated pharmacologically using in vitro models and a triple transgenic AD mouse model (3xTg-AD). In general, it was found that 2-(2-((2-chlorophenyl)amino)-2-oxoethyl)isoquinolin-2-ium chloride (**72b**) displayed an inhibition of Aβ aggregation by more than 30% (1 mM) and an over 80% inhibition of cholinesterase activity (100 µM). Compound **72b** penetrated the blood–brain barrier (BBB) and elevated acetylcholine levels in triple transgenic AD mice (3xTg-AD). Furthermore, **72b** significantly reduced the amyloid burden and cognitive deficits in 3xTg-AD mice after one month of treatment. As a result of **72b** treatment, animals showed reduced *tau* pathology, reduced amyloid precursor protein (APP) processing, and mitigated synaptic dysfunction [210].

**Figure 72:**
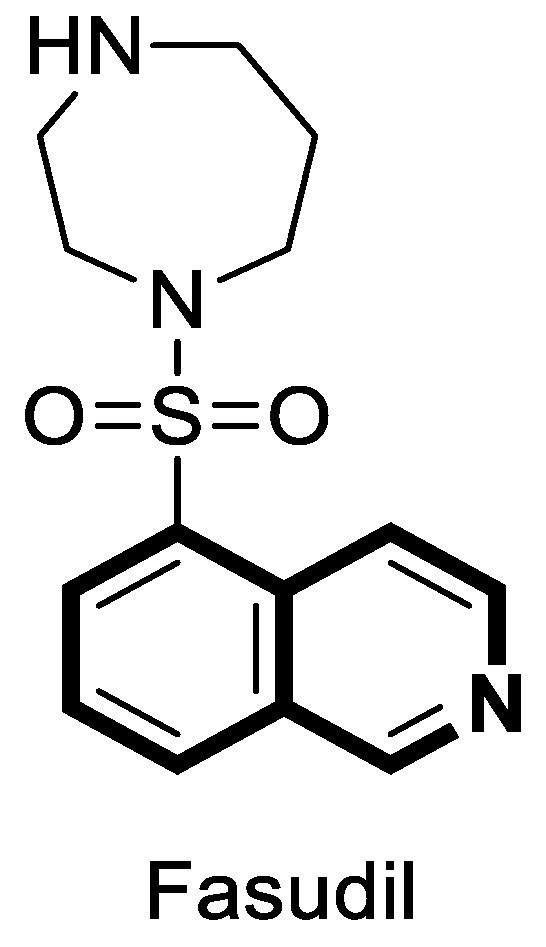
ROCK inhibitor—5-((1,4-diazepan-1-yl)sulfonyl)isoquinoline—Fasudil.

Much work has been performed on the pharmacological aspects of a small-molecule isoquinoline-based compound—Fasudil (Figure 72)—which has been approved for use in Japan and China since the year 1995 [211,212]. Fasudil belongs to a group of ROCK inhibitors, specific serine-threonine kinase (Rho-associated protein kinase). Such ROCK inhibitors have the ability to dilate blood vessels and are used to treat cerebral vasospasm, which often occurs after a subarachnoid hemorrhage.

Recently, it was found that Fasudil can also improve cognitive function in post-stroke patients and is still studied for potential benefits in other neurological conditions linked with the development of dementia, such as Alzheimer’s disease [213]. More is known about Alzheimer’s disease’s neuropathology. It is immediately clear that the progressive accumulation of extracellular β-amyloid (Aβ) plaques and intracellular neurofibrillary tangles (NFTs) involves different signaling pathways. Apart from the well-known signaling pathways, like *tau*-protein hyperphosphorylation or Aβ production, the modulation of ROCK though the activation of β-secretase may facilitate the formation of β-amyloid plaques. Moreover, ROCK signaling plays an important role in oxidative stress and neuroinflammation. Using specific inhibitors for RhoA-ROCK signaling, such as Fasudil, may help decrease the neuropathology associated with AD [214].

With regard to the subpopulation of patients suffering from Alzheimer’s disease presenting with an overload of copper, it may be noted that some chelating ligands that contain aromatic *N*-donor groups could potentially be used in Alzheimer’s disease therapies. It is known that an excess of Cu^2+^ can catalyze the production of reactive oxygen species and aggravate characteristic markers of Alzheimer’s disease progression.

**Figure 73:**
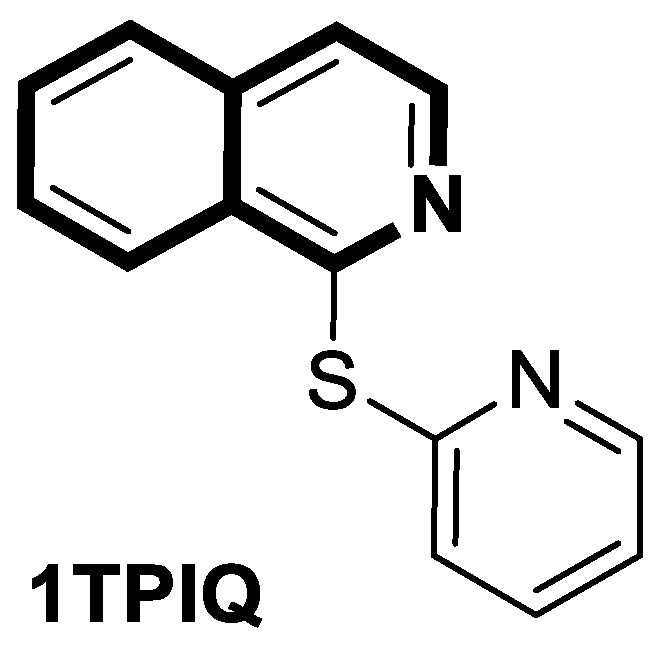
Chelator for Cu^2+^—1-(pyridin-2-ylthio)isoquinoline (**1TPIQ**).

Crnich and co-workers investigated the chelating ability of the sulfur-bridged isoquinoline-based ligand 1-(2′-thiopyridyl)isoquinoline (**1TPIQ**) obtained from 1-chloroisoquinoline and 2-mercaptopyridine in the presence of caesium carbonate (Figure 73) [215].

The effectiveness of **1TPIQ** was evaluated in the presence of different redox agents under physiological conditions. The authors measured the electrochemical response of the tested compound to copper, employing hydrogen peroxide (H_2_O_2_) and ascorbic acid (AA). The cyclic voltammetry (CV) results were compared with those obtained for similar *N*-donor aromatic ligands without sulfur atoms in their structure. The authors claimed that ROS production in a solution could be limited by sulfur-bridged ligands due to the altered Cu-electrochemistry in the presence of ascorbic acid.

**Figure 74:**
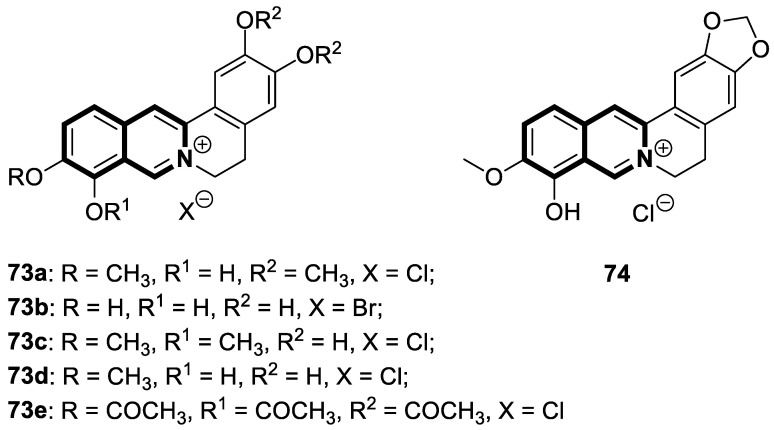
Six isoquinolinium derivatives, **73a–e** and **74**, based on palmatine and Berberine structures.

In recent years, there has been growing interest in the role of mitophagy in the development of neurodegenerative diseases. Mitophagy is a type of selective autophagy in which inefficient mitochondria are degraded. It has been shown that impaired mitochondrial function and the loss of mitochondrial capacity in nerve cells may contribute to their death. In Alzheimer’s and Parkinson’s diseases, several abnormalities related to the expression and function of proteins involved in this process have been observed. The modulation of mitophagy currently appears to be a crucial solution to maintaining the homeostasis of nerve cells [216,217]. It was found that isoquinoline-based alkaloids such as Berberine, Epiberberine, Palmatine, and Coptisine display mitophagy-inducing activity. Based on these premises, six isoquinolinium derivatives closely related to Palmatine and Berberine were synthesized by Um and colleagues through simple transformations of phenolic methyl and methylene ethers into corresponding hydroxyl groups (Figure 74) [218].

Among these six compounds, one 2,3,9,10-tetrahydroxy-5,6-dihydroisoquinolino[3,2-*a*]isoquinolin-7-ium bromide (**73b**) exerts specific mitophagy-inducing activity. Compound **73b,** with increased hydrophilicity, was obtained though the deprotection of all phenolic ether groups on Palmatine or Berberine with boron tribromide (BBr_3_).

Both in vitro and in vivo assays showed that isoquinoline derivative **73b** specifically induces mitophagy in human neuroblastoma cell line SH-SY5Y-expressing mutant APP through the ULK1-Rab9-dependent alternative pathway. Additionally, compound **73b** is nontoxic to mitochondria in SH-SY5Y cells. These effects were also estimated on transgenic mouse models of Alzheimer’s disease (five-FAD mutations and PS2APP), where **73b** reversed mitochondrial dysfunction and cognitive defects. Based on the findings, compound **73b** appears to be a potent agent in treating AD due to its ability to relieve mitochondrial dysfunction. The study also showed that the ULK1-Rab9 alternative mitophagy pathway can be considered a good therapeutic target. In view of the above, this compound is still the subject of further research [219].

### 3.2. Tetrahydroisoquinoline-Based Compounds

**Figure 75:**
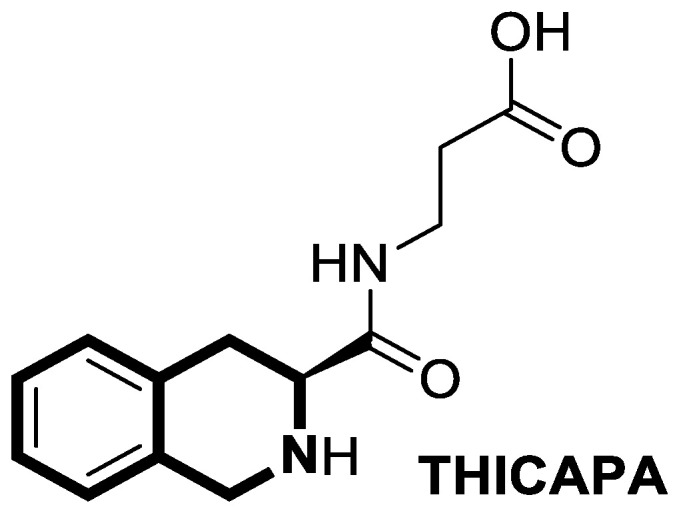
Chemical structure of **THICAPA**—(*S*)-3-(1,2,3,4-tetrahydroisoquinoline-3-carboxamido)propanoic acid.

Recently, Tan et al. reported an interesting tetrahydroisoquinoline derivative, also known under the acronymic name **THICAPA** (Figure 75) [220].

Chemically, **THICAPA**—(*S*)-3-(1,2,3,4-tetrahydroisoquinoline-3-carboxamido)propanoic acid—is composed of a THIQ scaffold and β-alanine chain. The neuroprotective potential of this compound was investigated by the Japanese Institute of Physical and Chemical Research, and data were collected by RIKEN Natural Products Depository (RIKEN NPDepo) [221]. **THICAPA** has proven to be the ligand for an amyloid-beta that reduces fibrillary Aβ42 aggregation and the accumulation of extracellular senile plaques. The biological properties of isoquinoline were performed using both in vitro and in vivo models—the PC12 cell model and transgenic *Drosophila melanogaster*-expressing human Aβ42 model. The treatment of neuronal cells (PC12) exposed to Aβ42 peptides (10 μM) with **THICAPA** at a concentration of 100 μM resulted in an over 92% cell viability. In comparison, the cell viability of controls treated with only Aβ42 was 40%. Additionally, in the *Drosophila* model, it was observed that cell proliferation, motor functions, and life span are enhanced. The authors indicated that **THICAPA**, due to the presence of β-alanine in its structure, exhibits an excellent cell-protective effectiveness, diminishing Aβ42-induced toxicity.

**Figure 76:**
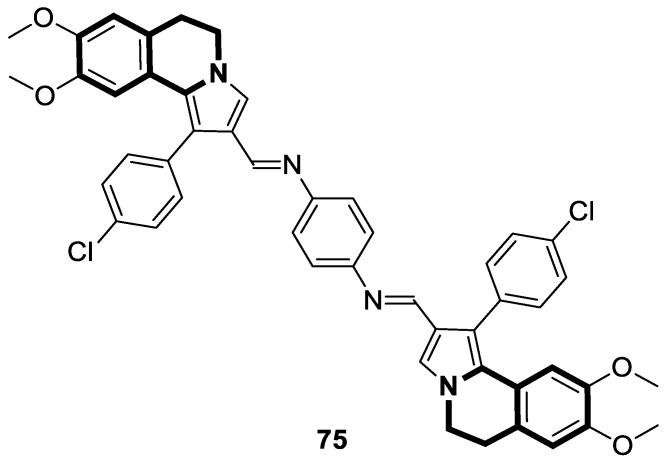
DHPPIQ-derived Schiff base **75** as a promising inhibitor of AChE.

The P-glycoprotein efflux transporter, associated with the blood–brain barrier (BBB), causes the vast majority of bioactive compounds not to permeate easily into brain tissue. Since molecules that circumvent the P-gp efflux pump can readily cross the BBB and reach the central nervous system, Nevskaya and colleagues investigated whether synthesized 5,6-dihydro-1-phenylpyrrolo[2,1-*a*]isoquinoline derivatives (DHPPIQs) might interfere with drug targets for neurological diseases, displaying promise as novel anti-Alzheimer drug candidates [222]. The authors supported their assumptions using the multi-fingerprint similarity search algorithm (MuSSeL) and expanded the series of previously reported derivatives with novel aliphatic and aromatic Schiff bases of DHPPIQ 2-carbaldehyde [223]. The MuSSeL predictions confirmed that the designed homobivalent Schiff bases assembled on 1,4-phenylenediamine could serve as novel multitarget-directed ligands (MTDLs) that target the proteins associated with Alzheimer’s disease (AChE, MAO, or Aβ40). It was found that DHPPIQ-derived Schiff base **75** (Figure 76) constitutes a promising inhibitor of acetylcholinesterase (AChE, IC_50_ = 7.3 µM, Ki = 4.69 μM) and prevents Aβ40 self-aggregation (IC_50_ = 13 μM). Furthermore, compound **75** showed moderate MAO A inhibitory properties (IC_50_ = 12.1 µM) and a low neuronal cytotoxicity (IC_50_ = 29.3 µM). The cell viability was estimated for the neuroblastoma cell line SH-SY5Y using camptothecin as a positive control (IC_50_ = 0.272 µM).

## 4. Anti-Inflammatory Activity

It is worth noting that isoquinoline can be a valuable lead molecule in the development of potent anti-inflammatory agents. Isoquinoline-derived alkaloids have proven to be rich sources of molecules with such properties. Research has shown that natural isoquinoline alkaloids display the potential to work on multiple mechanisms associated with inflammation. In this context, much of the efforts are focused on isoquinolines as synthetic analogs of these natural substances [224,225,226,227,228,229,230,231,232].

### 4.1. Dihydroisoquinoline-Based Compounds

The family of phosphodiesterases 1-11 (PDEs 1-11) constitutes intracellular enzymes that regulate the levels of cyclic monophosphates, such as cyclic adenosine monophosphate (cAMP) and cyclic guanosine monophosphate (cGMP). Specifically distributed in certain tissues, PDEs catalyze the hydrolysis of the second messenger molecules cAMP and cGMP and are associated with a wide spectrum of physiological processes [233]. It was found that PDEs may be related to certain pathological issues such as inflammation, autoimmune responses, metabolic dysfunction, or neurological disorders [234]. For example, PDE4 is present predominantly in epithelial cells, smooth muscle cells, and inflammatory cells. Studies have shown that patients with severe inflammation have an upregulated PDE4 expression in peripheral blood mononuclear cells (PBMCs) and inflamed tissues [235,236,237]. Inhibiting PDE4 promotes intracellular cAMP accumulation, which in turn regulates inflammation and maintains immune balance.

**Figure 77:**
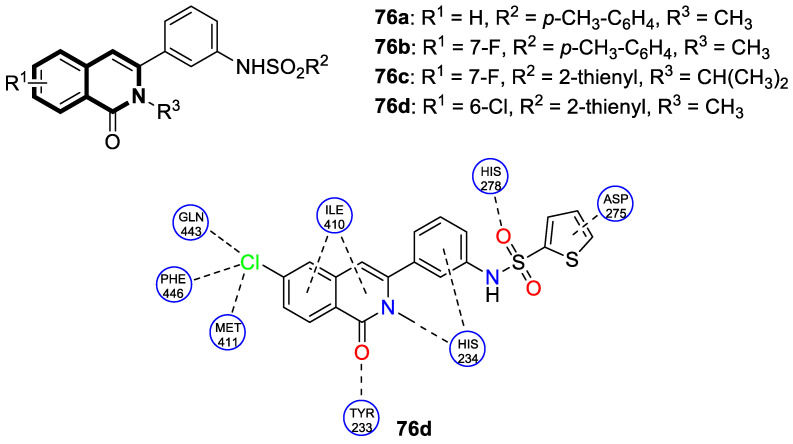
Novel PDE4 inhibitors **76a**–**d** and *N*-(3-(6-chloro-2-methyl-1-oxo-1,2-dihydroisoquinolin-3-yl)phenyl)thiophene-2-sulfonamide (**76d**) docked into the PDE4B using the iGEMDOCK.

Concerning the PDE4 inhibitors, Thirupataiah and co-workers reported a convenient approach for the synthesis of novel isoquinolin-1(2*H*)-one derivatives via a coupling–cyclization strategy (Figure 77) [238].

**Figure 78:**
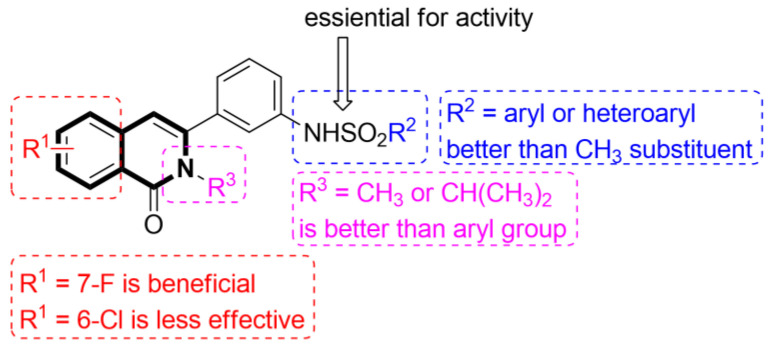
SAR of PDE4 inhibitors.

First, the inhibitory properties of the synthesized isoquinolin-1(2*H*)-ones (isocarbostyril) were tested in vitro at a concentration of 10 μM against the phosphodiesterase subtype B (PDE4B). In this assay, Rolipram was a positive control. Inhibition in the range of 46–90% was observed for compounds bearing the aminosulfonyl moiety (−NHSO_2_R_2_). A lack of this group in the structure resulted in less active compounds, displaying inhibition lower than 35% at 10 µM. Moreover, the structure–activity relationship (SAR) analysis (Figure 78) revealed that an aryl or thienyl scaffold was suitable instead of a methyl substituent (R^2^). Moreover, the methyl or isopropyl group (R^3^) was better than the aryl scaffold. On the other hand, a fluorine atom at the C-7 position of the isocarbostyril moiety was also beneficial, while a chlorine atom at the C-6 position appeared to decrease the compound’s activity. Among isoquinolin-1(2*H*)-ones, the compounds that showed inhibition higher than 75% were chosen as ‘*hit molecules*’ for further evaluations at various concentrations. The most active ones, **76a** (R^1^ = H; R^2^ = *p*-CH_3_-C_6_H_4_; R^3^ = CH_3_), **76b** (R^1^ = 7-F; R^2^ = *p*-CH_3_-C_6_H_4_; R^3^ = CH_3_), **76c** (R^1^ = 7-F; R^2^ = 2-thienyl; R^3^ = CH(CH_3_)_2_), and **76d** (R^1^ = 6-Cl; R^2^ = 2-thienyl; R^3^ = CH_3_), achieved IC_50_ values of 3.77 µM (PDE4B: 82.86%), 2.43 µM (PDE4B: 89.93%), 3.26 µM (PDE4B: 79.42%), and 3.63 µM (PDE4B 82.80%), respectively (Figure 77). These compounds also show anti-inflammatory effects when tested against TNF-α in vitro. The most prominent compound, **76b**, showed an inhibition of TNF-α in the range of 29.8% to 60.5% (0.3–10 µM). The reference drug, Rolipram, at a concentration of 0.1 µM, displayed 50.9% of TNF-α inhibition.

The authors supported the in vitro studies with in silico experiments, which suggested favorable interactions with the PDE4B protein. In the case of compounds **76b** and **76c**, the C=O group present in the isocarbostyril moiety and the N-H of the aminosulfonyl group formed hydrogen bonds with the glutamine 443 and histidine 234 residues, respectively. Other interactions between amino acid residues, such as phenylalanine, isoleucine, tyrosine, and methionine, and docking compounds in the catalytic site of PDE4B include *π*-σ, *π*-*π* stacking, hydrophobic, and van der Waals.

Interestingly, in the case of molecule **76d** (Figure 77), shifting the halogen atom from position C-7 to C-6 altered the interaction pattern. This molecule creates two hydrogen bonds with tyrosine 233 and glutamine 443 through a carbonyl group and a halogen atom, respectively. Moreover, in silico docking studies demonstrated additional interactions with isoleucine 410, methionine 411, phenylalanine 446, histidines 234 and 278, and aspartic acid 275. It should be stressed that the weaker nature of the hydrogen bonds formed by chlorine in comparison to the bond created by fluorine could be the reason for the slightly lower inhibitory activity of compound **76d**.

**Figure 79:**
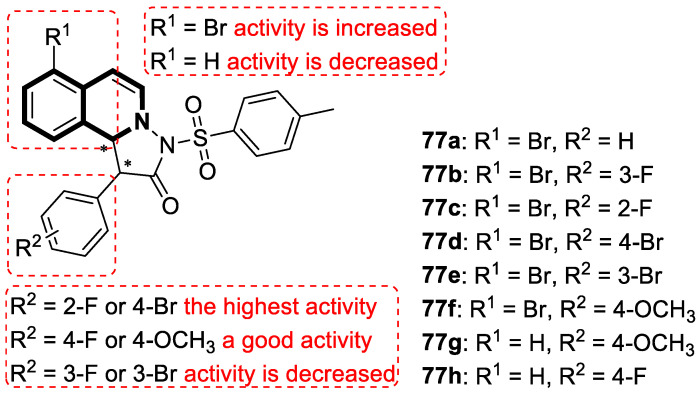
Designed 1-phenyl-3-tosyl-1,10*b*-dihydropyrazolo[5,1-*a*]isoquinolin-2(3*H*)-ones **77a**–**h**, with anti-inflammatory activity.

In 2023, Akhtar and colleagues [239] obtained promising anti-inflammatory results concerning fused pyrazolo-isoquinolines **77a**–**h** (Figure 79). A proposed mechanism of anti-inflammatory activity of synthesized 1-phenyl-3-tosyl-1,10*b*-dihydropyrazolo[5,1-*a*]isoquinolin-2(3*H*)-ones is associated with the inhibition of inducible nitric oxide synthase (iNOS) in a dose-dependent manner. By releasing nitric oxide from arginine, iNOS stimulates certain pro-inflammatory cytokines which generate a systematic inflammatory response syndrome. Compound **77d** was the most active one, reaching an IC_50_ value of 20.76 μM. The reference positive control was celecoxib (IC_50_ = 23.1 μM).

Based on an SAR analysis, it was found that the anti-inflammatory potency of pyrazolo-isoquinolines may be related to the polarizability, location, and steric effects of substituents on the phenyl ring. For example, their activity depends on the presence and position of a halogen atom. The presence of two bromide atoms increases its anti-inflammatory capacity (compound **77d**). Derivatives **77b** (IC_50_ = 33.8 μM) and **77h** (low activity at concentration of 80 μM) showed a weaker activity due to fluoro substitution at different positions, which appeared to be less favorable for their activity.

### 4.2. Tetrahydroisoquinoline-Based Compounds

**Figure 80:**
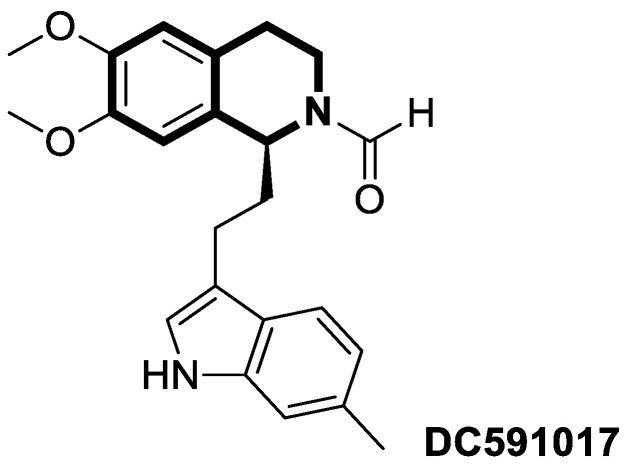
Tetrahydroisoquinoline-based phosphodiesterase-4 (PDE4) inhibitor **DC591017**.

According to recent findings on tetrahydroisoquinoline-based phosphodiesterase-4 (PDE-4) inhibitors with anti-psoriatic properties [240], Li and co-workers designed a highly selective PDE-4 inhibitor—(*S*)-6,7-dimethoxy-1-(2-(6-methyl-1*H*-indol-3-yl)ethyl)-3,4-dihydroisoquinoline-2(1*H*)-carbaldehyde **DC591017** (Figure 80)—which demonstrated an excellent in vitro and in vivo anti-inflammatory activity [241].

Compound **DC591017** showed a strong inhibition both on PDE4 (IC_50_ = 0.24 μM) and TNF-α release (IC_50_ = 0.65 μM) from LPS-stimulated PBMCs. This derivative notably decreased TNF-α in a dose-dependent manner, increased the secretion of anti-inflammatory interleukin-10 (IL-10) from LPS-stimulated murine macrophage-like cells (RAW 264.7 macrophages), and markedly upregulated the intracellular cAMP concentration. In addition, CREB (cAMP response element-binding protein) phosphorylation was promoted by **DC591017** without affecting the total protein levels. Furthermore, this was confirmed by immunofluorescent staining. The high selectivity of the isoquinoline derivative towards the PDE-4 isoform was confirmed through an analysis of the co-crystal structure of the compound with protein, showing interactions of methoxy groups of a tetrahydroisoquinoline scaffold with a conservative glutamine 369 and the formation of hydrogen bonds between carbonyl group and histidine 160. Moreover, the introduction of a methyl group on the indole ring contributes to the formation of a more stable interaction in the hydrophobic pocket of the protein, which is in agreement with the thermodynamic profiles determined through isothermal titration calorimetry (ITC).

Cyclooxygenase or COX is an enzyme involved in the biosynthesis of prostaglandins—endogenously produced molecules with strong pro-inflammatory properties. An effective method of reducing inflammation and pain is to inhibit COX pharmacologically [242]. This enzyme is found in two isozymic forms, known as cyclooxygenase-1 (COX-1) and cyclooxygenase-2 (COX-2). COX-1 is normally constitutively expressed under normal healthy conditions and is present in most tissues, whereas COX-2 as an inducible isoform is activated during inflammation. Therefore, COX-2 has become a target for compounds that could relieve certain pathological conditions by reducing the production of pro-inflammatory prostaglandins. In this context, it could be beneficial in selective COX-2 blockage over COX-1. It may also reduce the gastrointestinal adverse effects that are associated with non-specific COX inhibition—these include pathologies such as mucosal damages and small intestine hemorrhages [243,244].

**Figure 81:**
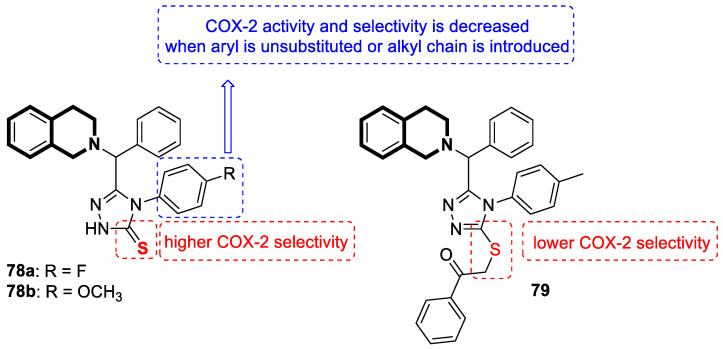
Hybrids composed of tetrahydroisoquinoline moiety and 1*H*-1,2,4-triazole-5(4*H*)-thione or 4*H*-1,2,4-triazol-3-yl)thio)-1-phenylethan-1-one scaffolds with anti-inflammatory properties.

In 2024, a novel series of 1,2,4-triazole-tetrahydroisoquinoline hybrid compounds was synthesized and evaluated as potent inhibitors of COX-1 and COX-2 [245]. Hybrid compounds **78a**, **78b**, and **79** (Figure 81) were designed based on structure–activity relationship studies of classical non-steroidal anti-inflammatory drugs (NSAIDs). It was assumed that a heterocyclic core that is directly connected to diaryl moieties is responsible for COX-2 selective inhibition. Moreover, one of these aromatic rings must have a sulphonamide or sulfone substitute at the para position. The acidic carboxylic group taken from conventional NSAIDs in this series of compounds was replaced with a stable 1,2,4-triazole as a less ulcerogenic heterocyclic bioisostere to decrease local GI side effects.

Synthesized hybrid compounds displayed a lower than reference Indomethacin activity on COX-1. This may suggest an acceptable safety profile and lower gastrointestinal adverse effects. The most effective against COX-2 were compounds **78a**, **78b**, and **79**, which were the most potent derivatives in this study against COX-2, with IC_50_ values of 0.87, 1.27, and 0.58 µM, respectively. In comparison, the estimated IC_50_ value for reference celecoxib was 0.82 µM. Interestingly, these three compounds have moderate selectivity indices (3.66–13.6) compared to celecoxib; SI = 18.3. The authors suggest that these results may be beneficial in terms of minimizing the cardiovascular side effects associated with highly selective COX-2 inhibitors.

## 5. Antidiabetic Activity

Diabetes mellitus is classified as a metabolic disease characterized by high blood glucose levels. There are two types of the disorder: type 1 and type 2. Type 1 diabetes mellitus (T1DM) is characterized by hyperglycemia resulting from defects in insulin secretion. High levels of sugar result from inappropriate hormone action—in particular, insulin resistance and decreased insulin secretion from the pancreas are observed in type 2 diabetes mellitus (T2DM). Long-term diabetes mellitus and chronic hyperglycaemia can damage, malfunction, or fail the critical organs, particularly the eyes, kidneys, nerves, heart, and blood vessels. Consequently, managing, controlling, and treating the disease is difficult. It depends mostly on precise blood glucose monitoring, an appropriate diet, frequent exercise, and effective and safe medications. The treatment of comorbid conditions such as hypertension or hyperlipidemia is also crucial. Estimated data show that T2DM affects millions of patients worldwide. Its heavy burden has been demonstrated by high morbidity and mortality [246].

**Figure 82:**
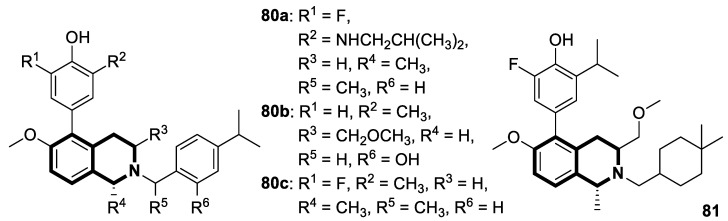
Novel 6-methoxy-3,4-dihydro-1*H*-isoquinolines **80a**, **80b**, **80c**, and **81**, useful in the treatment of diabetes.

Ordinarily, hyperglycemia can be treated in a variety of ways, including the administration of exogenous insulin, sulfonylurea derivatives as secretagogues agents, biguanides for increasing hepatic sensitivity to the pancreatic hormone, or the administration of insulin sensitizers—thiazolidinediones. A promising approach to diabetic patients’ treatment includes the delivery of insulinotropic peptides belonging to the incretin family, e.g., glucagon-like-peptide-1 (GLP-1). The benefits of incretin hormones, such as GLP-1, in lowering blood glucose have gained increased attention in recent years. This peptide with incretin hormone activity is secreted by intestinal enteroendocrine L-cells. In response to glucose, GLP-1 stimulates the release of insulin from pancreatic beta cells. Therefore, T2DM can be treated with a number of recently approved GLP-1 receptor agonists (GLP-1 RAs). In response to glucose consumption, a glucose-dependent insulinotropic polypeptide (GIP) produced in the upper small intestine stimulates insulin release from the pancreas. Recently, a novel dual-glucose-dependent insulinotropic peptide (GIP) and GLP-1 RA, tirzepatide, was approved in 2022 for the treatment of diabetes as a subcutaneous injectable. However, there is still a need for novel agents to treat diabetes and its complications. Examples of such compounds are represented by novel 6-methoxy-3,4-dihydro-1*H*-isoquinolines **80a**, **80b**, **80c**, and **81** (Figure 82) [247]. The pharmaceutical composition comprising compounds **80a**, **80b**, **80c**, and **81** and their pharmaceutically acceptable salts according to the patent document WO/2022/076503 [248] may be used as positive allosteric modulators (PAMs) of the glucagon-like peptide-1 (GLP-1) receptor (GLP-1R) and glucose-dependent insulinotropic polypeptide (GIP) receptor (GIPR) (Figure 82).

A ligand-dependent nuclear receptor, the peroxisome proliferator-activated receptor (PPAR), is a regulator of adipocyte differentiation that plays a crucial role in adipocyte transformation and proliferation [249]. Compounds with PPARγ agonistic activity increase adiponectin and decrease tumor necrosis factor α (TNFα) levels, enhancing insulin sensitivity. Since PPARγ agonists reduce insulin resistance in type 2 diabetics, they may be used as effective antidiabetic drugs [250]. Unfortunately, PPARγ full activation increases the risk of heart failure and bone fracture. Thus, PPARγ partial agonists or PPARγ modulators—compounds that partially activate PPARγ and antagonize PPARγ activation by PPARγ full agonists—are considered to be a safe treatment option. Such PPARγ modulators, which fully induce insulin sensitization, partially activatating PPARγ, display no adverse effects [251].

**Figure 83:**
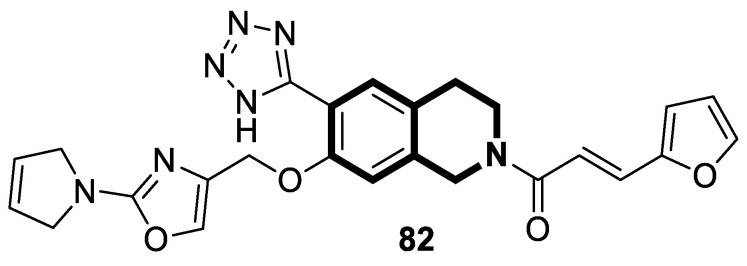
Selective peroxisome proliferator-activated receptor partial agonist 2,7-substituted-6-tetrazolyl-1,2,3,4-tetrahydroisoquinoline derivative **82**, useful in the treatment of diabetes.

The benefits of PPARγ modulation in diabetes have increasingly been studied for many years. In 2021, Morishita and co-workers synthesized a series of novel 2,7-substituted-6-tetrazolyl-1,2,3,4-tetrahydroisoquinolines as selective PPARγ partial agonists [252]. The identified 1-(7-((2-(2,5-dihydro-1*H*-pyrrol-1-yl)oxazol-4-yl)methoxy)-6-(1*H*-tetrazol-5-yl)-3,4-dihydroisoquinolin-2(1*H*)-yl)-3-(furan-2-yl)prop-2-en-1-one (**82**) (Figure 83)—an analog of the previously reported 1,2,3,4-tetrahydroisoquinoline derivative [253]—exhibited strong PPARγ agonist (EC_50_ = 6 nM) and antagonist activities (IC_50_ = 101 nM).

The in vivo tests with male Zucker fatty rats treated with compound **82** at 10 and 30 mg/kg for 4 weeks revealed—similar to Pioglitazone—reductions in plasma triglyceride levels, increases in plasma adiponectin levels, and the attenuation of increases in plasma glucose levels in the Oral Glucose Tolerance Test (OGTT). Derivative **82** showed optimal oral absorption and metabolic stability (C_max_ = 11.4 μg/mL). Compared to the reference, Pioglitazone, the tested compound **82** did not reduce the hematocrit value at a dose of 30 mg/kg. The results of this study show that the tetrahydroisoquinoline derivative **82** was equal to Pioglitazone in terms of insulin-sensitizing properties, with minor adverse effects.

The docking studies of 1,2,3,4-tetrahydroisoquinoline **82** to the PPARγ protein have shown that the tetrazolyl ring forms a hydrogen bond with serine 342 and interacts electrostatically with the positively charged side chain of arginine 288. It was found that the 2,5-dihydro-1*H*-pyrrol-1-yl substituent on the oxazole ring sterically fits into the subpockets composed of cysteine 285, serine 289, isoleucine 326, tyrosine 327, leucine 330, phenylalanine 363, and methionine 364.

## 6. Other Biomedical Applications

Although the synthetic or semisynthetic isoquinoline-based derivatives may find applications in the medicinal field due to their wide range of pharmacological activities, they have also attracted considerable attention due to their optical or fluorescent properties. Thus, isoquinolines can also broadly be used in biology and chemistry as fluorescent sensors or probes. Fluorescence-labeled probes have great potential in this area, since fluorescence microscopes can directly visualize drug uptake. It has been proven that fluorescently labeled natural compounds such alkaloids can be used to visualize biological processes in living systems using cells or tissues [254,255].

Recently, positron emission tomography (PET)—a functional imaging technique using radiotracers to visualize and measure changes in biochemical and metabolic processes or physiological activity—has attracted considerable attention. For example, when the brain is affected by pathologies such as Alzheimer’s disease (AD), both glucose and oxygen metabolism are greatly reduced. On the other hand, at various stages of Alzheimer’s disease, *tau* protein—which normally helps maintain the proper structure of nerve cells and function of axons—may undergo abnormal phosphorylation and aggregation. This leads to the formation of neurofibrillary tangles and damage to neurons. Therefore, PET of the brain may also be used to perform an early diagnosis of AD [256,257].

### 6.1. Isoquinoline-Based Compounds

**Figure 84:**
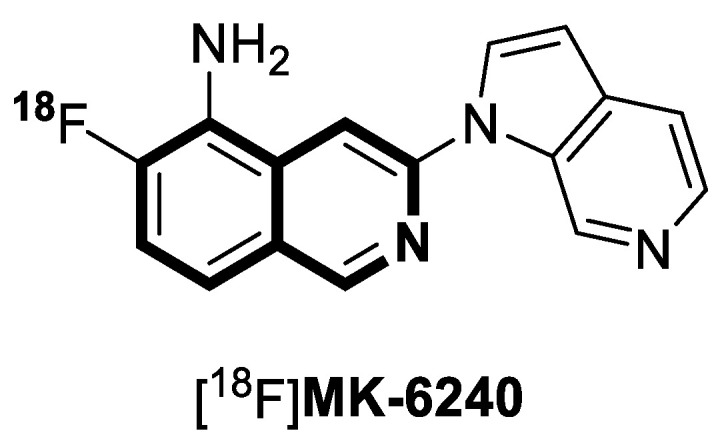
[^18^F]**MK-6240**—6-fluoro-3-(1*H*-pyrrolo[2,3-*c*]pyridin-1-yl)isoquinolin-5-amine—a promising PET tracer for in vivo NFT quantification.

Depicted in Figure 84, 6-^18^fluoro-3-(1*H*-pyrrolo[2,3-*c*]pyridin-1-yl)isoquinolin-5-amine or [^18^F]**MK-6240** is a second-generation *tau* PET tracer that targets neurofibrillary tangles (NFTs), which may be used for the detection and quantification of in vivo cerebral *tau*-pathology in AD (*K*_D_ = 0.42 nM) [258]. Isoquinoline derivative [^18^F]**MK-6240** displays a high affinity for tangled tau filaments and is frequently used in clinical trials to detect tau protein tangles in the brain, which are characteristic of Alzheimer’s disease and other neurodegenerative diseases.

### 6.2. Tetrahydroisoquinoline-Based Compounds

The altered levels of transient metal ions such as copper or iron in living cells are associated with severe pathological conditions, including cancer and neurodegeneration. Copper appears to contribute to the aggregation of amyloid peptides and their neurotoxicity. The second essential metal is iron, which is involved in basic physiological and catalytic processes that include oxygen transport, nitrogen conversion by nitrogenases, metabolism, and signaling. An excess of iron may result in hemochromatosis, liver disorders, diabetes, and even cancer. In light of this, copper or iron determination is vital for both their presence in the environment and potential hazards. Fluorescent sensors proved to be powerful tools for the detection of extremely low levels of particular metal ions through colorimetric or chelation-induced enhanced fluorescence (CHEF), or ‘turn-on’ and chelation enhancement quenching effect (CHEQ) or ‘turn-off’ responses. It should be noted that, in ‘turn-off’ response chemosensors, copper chelation may induce intrinsic fluorescence quenching. For this reason ‘turn-on’ sensors with fluorescence enhancement are more promising due to their simplicity [259].

**Figure 85:**
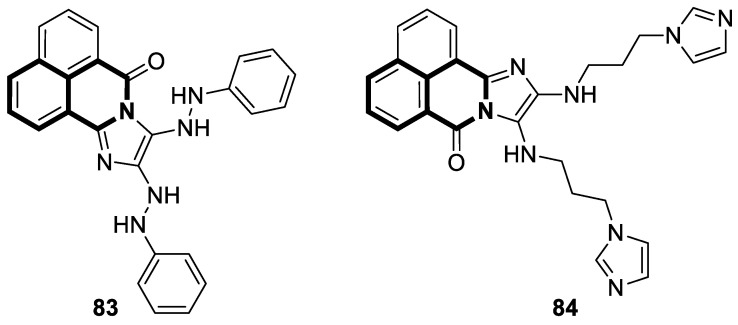
Fluorescence sensors **83** and **84** are suitable tools for detecting Cu^2+^, Cl^−^, and Fe^3+^ ions.

In 2025, Murugaperumal and co-workers reported novel isoquinoline-fused benzimidazole derivatives **83** and **84** (Figure 85), which may find applications as highly selective sensors for detecting copper (Cu^2+^), iron (Fe^3+^), and chloride (Cl^−^) ions [260].

The designed probes **83** and **84** contain donor nitrogen atoms that are capable of binding metals. The compounds display a high selectivity towards copper, iron, and chlorine. Upon the chelation of Cu^2+^ or Cl^−^, a significant increase in the fluorescence of **83** was detected. When **84** was treated with Fe^3+^, a bright-red- to dark-brown-colored solution was observed, suggesting that **83** and **84** could serve as potential detecting systems for Cu^2+^, Fe^3+^, or Cl^−^ ions. Furthermore, the effect of **83** and **84** on HepG2 cells was assessed in order to include them in biological networks. Both compounds displayed a good cell permeability. Based on estimated IC_50_ values, it may be concluded that **83** (IC_50_ = 154.5 μg/mL) is less toxic than **84** (IC_50_ = 95 μg/mL). HepG2 cells treated with **83** and **84** exhibited blue fluorescence.

**Figure 86:**
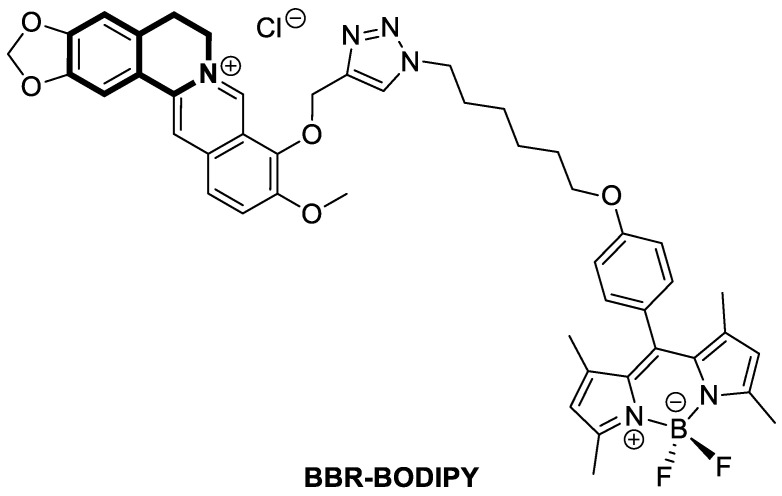
Fluorescence sensor **BBR-BODIPY** based on Berberine.

Jin and colleagues demonstrated the potential for the fluorescently labeled natural alkaloid Berberine in subcellular localization studies [261]. In this work, which may inspire ideas for the further improvement of isoquinoline-related compounds as fluorescent probes, alkylated Berberine was attached to the 4,4-difluoro-4-bora-3a,4a-diaza-s-indacene derivative to produce **BBR-BODIPY** (Figure 86). The authors assessed its cellular uptake and mitochondria targeting ability in human breast cancer cells (MCF7).

The probe **BBR-BODIPY** was found to rapidly penetrate MCF7 human breast carcinoma cells at low concentrations, and green fluorescence was observed in the tested cells while they were incubated for 5 min. A significant increase in Bax, cytochrome C, and cleaved caspase-9 levels was demonstrated in MCF7 cells treated with Berberine and **BBR-BODIPY**. A rise in cleaved caspase-3 and its substrate, cleaved PARP, was associated with the decreased expression of Bcl2 and Pro-caspase-3. Compared with free Berberine, **BBR-BODIPY** induced larger changes in protein levels, suggesting the improved anticancer effects of Berberine when modified with a 5,5-difluoro-1,3,7,9-tetramethyl-10-phenyl-5*H*-dipyrrolo[1,2-*c*:2′,1′-*f*][1,3,2]diazaborinin-4-ium-5-uide fluorescent probe. In addition to providing insight into the molecular mechanisms of the anticancer activity of Berberine, these results could help develop new drugs based on Berberine to treat cancer.

## 7. Metal Complexes of Isoquinoline-Based Compounds with Biological Activities

The isoquinoline core can serve as a neutral monodentate ligand in the formation of metal complexes through the lone pair of electrons of the donor-character aromatic nitrogen atom. Among all transient metals, platinum, ruthenium, iridium, and copper are especially prominent, since their complexes exert pharmacological activity, specifically cytotoxicity. Therefore, they attract attention as promising antitumor agents. In comparison with their parent ligands, these coordination compounds may exert particularly enhanced biological potency. Also, these complexes were reported to be active against drug-resistant tumor cells and are excellent agents for preventing chemoresistance [262,263,264].

### 7.1. Anticancer Agents

**Figure 87:**
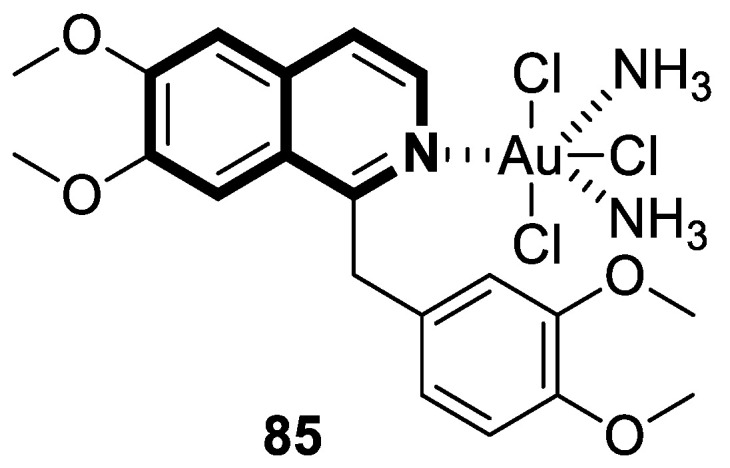
Au(III)–Papaverine complex **85** with anticancer activity.

Recently, Gaber and colleagues described a novel gold complex that was synthesized starting with Papaverine hydrochloride and gold(III) chloride in methanol [265]. The Au(III)–Papaverine complex **85** (Figure 87) showed cytotoxic activity against MCF-7 and Hep-G2 cell lines. Towards MCF-7, the complex exhibited a stronger anticancer activity (IC_50_ = 2.87 μg/mL) than the reference drug Cisplatin (IC_50_ = 5.71 μg/mL). As a result of these findings, Papaverine alkaloid compounds are more potent after complexation with metal ions.

**Figure 88:**
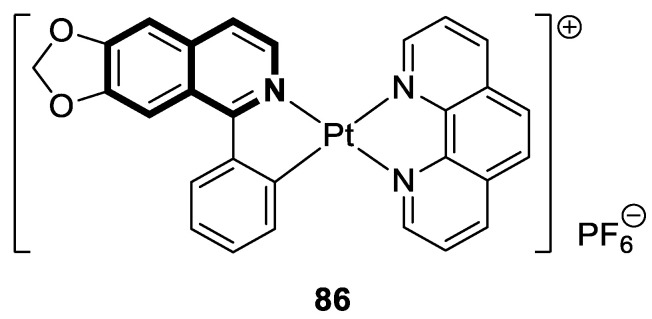
Cytotoxic Pt(II)-complex **86** with isoquinoline-based ligand.

Wang and co-workers synthesized four mononuclear cycloplatinated(II) complexes with an isoquinoline-based ligand—5-phenyl-[1,3]dioxolo[4,5-*g*]isoquinoline—and different types of auxiliary *N*^*N* ligands, which were introduced to modify the compound’s electronic and hydrophobic properties. Among them, the Pt(II)-complex **86** with the 1,10-phenanthroline *N*^*N* ligand depicted in Figure 88 exhibited selective cytotoxicity against triple-negative breast cancer (TNBC) and improved in vivo potency to Cisplatin in a mouse model [266].

The authors suggested that the platinum complex **86** could trigger the expression of autophagy-associated proteins, leading to autophagy-dependent cellular ferritin degradation. In this way, the **86** chelate can initiate ferritinophagy-dependent ferroptosis. As a result of this process, ferritin is degraded via a selective form of autophagy (ferritinophagy), releasing free iron into the cell. Moreover, it was demonstrated that, in MDA-MB-231 cells upon **86** treatment, the levels of nuclear receptor coactivator 4 (NCOA4)—a cargo receptor which targets ferritin for degradation—were decreased in a time-dependent manner. By utilizing NCOA4, the autophagy machinery recognizes ferritin and delivers it to lysosomes for degradation, increasing the labile iron pool (LIP) through ferritin liberation.

In chemotherapy, the search for ruthenium complexes as alternatives to platinum compounds is proposed because of their unique properties. In contrast to Cisplatin and its analogs, ruthenium-based compounds could be cytotoxic in both *trans* and *cis* configurations. Such complexes are promising not only as anticancer drug candidates but also as photosensitizers [267,268,269,270,271].

**Figure 89:**
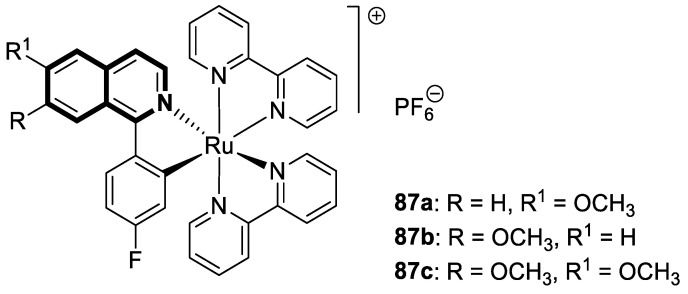
Ruthenium(II) cyclometalated complexes **87a**, **87b**, and **87c**.

In this line, Chen and co-workers focused on ruthenium(II) cyclometalated anticancer complexes bearing 1-(4-fluorophenyl)isoquinoline (1-(4-F-Ph)-IQ) and 2,2′-bipyridine (bpy) scaffolds [272]. Synthesized ruthenium(II)-chelates **87a**, **87b**, and **87c** (Figure 89) were found to exhibit a higher cytotoxic potency than Cisplatin towards the following cell lines: human lung carcinoma (A549), Cisplatin-resistant lung carcinoma epithelial cell line (A549/DDP), human liver cancer cell line (HepG2), and human breast cancer cell line (MCF-7). Also, these compounds displayed a high selectivity against cancer cells compared with non-tumorigenic cells.

Particularly of interest as a candidate for further anticancer drug development is the complex containing the 6,7-dimethoxyisoquinoline scaffold [Ru(bpy)2(1-(4-F-Ph)-6,7-(OCH_3_)_2_-IQ], **87c**, which had a superior activity compared with Cisplatin. Inductively coupled plasma mass spectrometry (ICP-MS) quantification showed that the penetration and cellular uptake of complex **87c** in A549 and A549/DDP tumor cells is different from Cisplatin. This may be explained by their higher lipophilicity than Cisplatin, which is correlated with metal cellular content (log*P*_o/w_ = 1.25 vs. log*P*_o/w_ = −4.53 measured for Cisplatin). Complex **87c** displayed in vitro low toxicity towards the human bronchial epithelial cell line (HBE) and zebrafish embryos in vivo. With respect to both A549 and A549/DDP cell lines, **87c** showed the highest cellular uptake and a higher antiproliferative potency. Moreover, **87c** was predominantly distributed in the nucleus when absorbed by A549/DDP cells. Additionally, it was found that **87c** can induce ROS-mediated apoptosis by disrupting mitochondrial function, damaging DNA, and arresting the cell cycle in S and G2/M phases in A549 and A549/DDP cells. Signaling pathway analysis indicated that ruthenium(II) complexes can be developed as efficient nuclear factor erythroid 2-related factor 2 (Nrf2) inhibitors and can overcome the Cisplatin resistance in a highly metastatic and aggressive subtype—non-small-cell lung cancer (NSCLC).

**Figure 90:**
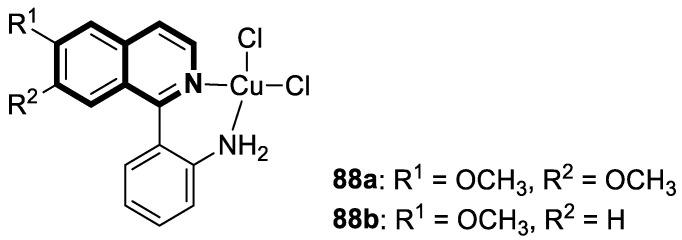
Cytotoxic copper(II) complexes **88a** and **88b**.

Another transient metal—copper—has also emerged as a potential and valuable alternative to platinum. Two mononuclear copper complexes, **88a** and **88b**, depicted in Figure 90, containing 2-(6,7-dimethoxyisoquinolin-1-yl)aniline and 2-(6-methoxyisoquinolin-1-yl)aniline ligands, were synthesized in 2020 by Gul and co-workers [273]. These complexes were evaluated against a broad spectrum of tumor cell lines, including MGC80-3, T-24, SKOV-3, HeLa, A549, BEL-7704, and MDA-MB-216 in the MTT colorimetric assay (MTT). To further confirm the safety of these complexes, non-cancer human liver cells, HL-7702, were used.

It should be mentioned that synthesized Cu(II) compounds **88a** and **88b** showed a higher effectiveness against the human lung adenocarcinoma cell line (A549) (IC_50_ = 3.05 μM for **88a** and IC_50_ = 6.04 μM for **88b**) than the corresponding ligands (IC_50_ = 52.0 μM for **88a**-**ligand** and IC_50_ = 60.1 μM for **88b**-**ligand**) and the reference Cisplatin (IC_50_ = 20.7 μM). The least susceptible to the effects of the tested complexes was the human gastric cancer cell line (MGC-803) (IC_50_ = 21.3 μM for **88a** and IC_50_ = 36.2 μM for **88b**).

It has been demonstrated that **88a** and **88b** induce cytotoxicity through mitochondrial-mediated apoptosis, leading to caspase cascades, reactive oxygen species generation, and ER stress, ultimately leading to cell death through the MAPK-mediated pathway. Aside from that, compounds **88a** and **88b** induce autophagy by regulating proteins, such as Beclin 1, p62, and LC3, which are crucial for the autophagy process. Finally, the in vivo anticancer experiments using a xenograft mouse model showed that Cu(II) complexes **88a** and **88b** effectively inhibit A549 tumor growth (21.1% and 41.3% for **88a** and 19.5% and 41.7% for **88b**), exhibiting a higher in vivo safety profile than Cisplatin during the 15-day treatment. This makes these isoquinoline-based copper(II) complexes potentially useful for the development of anticancer drugs.

Cancer-specific immune responses can be activated by several clinically used chemotherapeutic drugs, such as Oxaliplatin, by inducing immunogenic cell death (ICD). Thus, the concept of ICD has received considerable interest as a potential synergistic cancer chemotherapy approach. The induction of ICD may vary depending on the type of tumor, so the current ICD-inducing drugs are insufficient [274].

**Figure 91:**
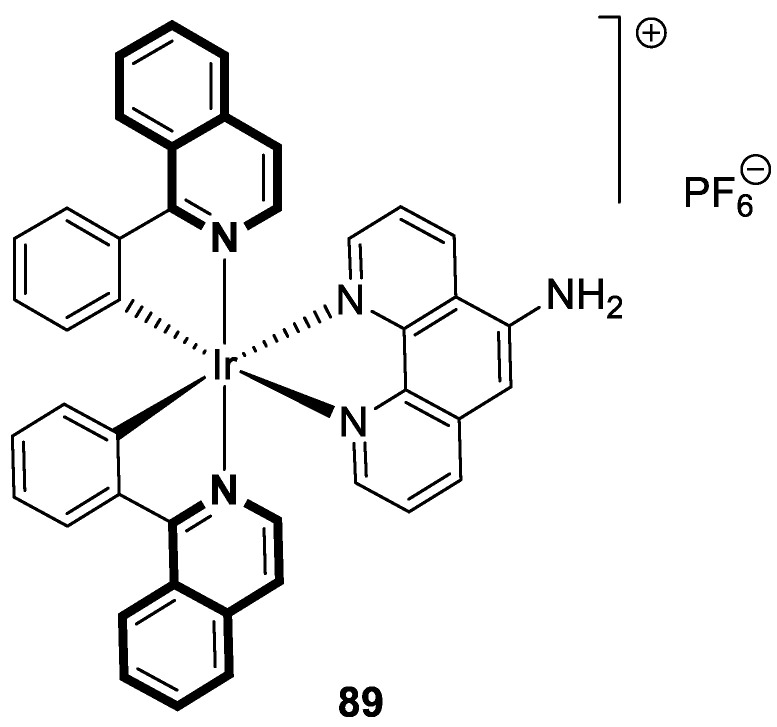
Iridium(III) complex **89** composed of 1-phenylisoquinoline and 1,10-phenanthrolin-5-amine.

In this line, several early studies have shown that iridium(III) complexes that display cytotoxic activity attract attention as promising antitumor agents. In particular, they cause tumor cells to undergo apoptosis via ER targeting, along with the immunomodulation of the ICD to promote tumor cell death. Chen et al. described, in 2021, cyclometalated iridium(III) complex **89**, depicted in Figure 91, composed of 1-phenylisoquinoline (piq) and 1,10-phenanthrolin-5-amine moieties [275]. This Ir(III)-complex **89** was synthesized and reported by Lu and collaborators in 2014 [276]. Multiple methods have been used to evaluate its anticancer effects, including subcellular organ localization, mitochondrial dysfunction, ROS production, and mitochondria-associated signaling pathways, as well as autophagy, also known as ‘self-eating’, coordinated with apoptosis.

Upon 24 h of treatment in the presence of **89** at a concentration of 10 μM, the proportion of A549 cells showing atypical nuclei increased to 53.5%. Due to its excellent photophysical properties, **89** was capable of detection through fluorescence microscopy. Compound **89** also showed a moderate antibacterial activity against the representative Gram-positive strain *Staphylococcus aureus* and Gram-negative strain *Psuedomonas aeruginosa*.

**Figure 92:**
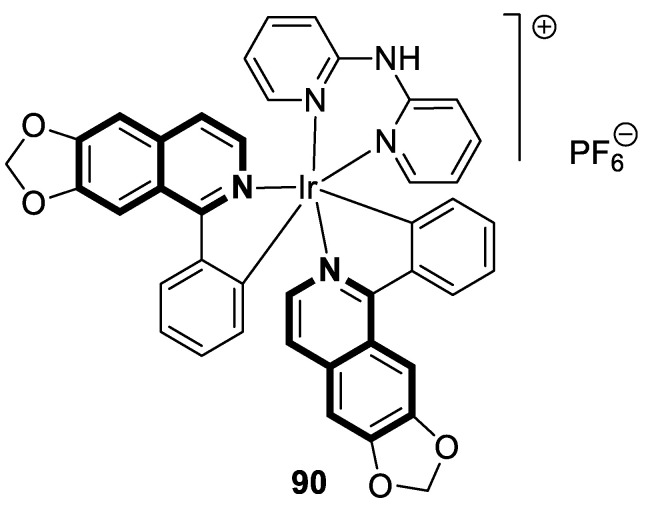
The cationic cyclometalated Ir(III) complex **90**.

A challenge remains in developing anticancer drugs for treating triple-negative breast cancer (TNBC) due to the fact that TNBC is, however, not considerably affected by a large number of drugs that trigger an ICD response. None of the drugs exert a large clinical effect on the disease. A promising candidate for chemoimmunotherapy to combat triple-negative breast cancer (TNBC) is an octahedral cyclometalated iridium(III) complex based on structurally diverse CN and NN ligands **90** (Figure 92) [277]. The structure of novel Ir(III)-complex **90** designed by Lu was confirmed through single crystal X-ray analysis. Compound **90** is composed of two 3,4-methylenedioxy-1-phenylisoquinolines and an ancillary bidentate di(pyridin-2-yl)amine molecule. Introducing a phenyl group to an isoquinoline scaffold at position 1 facilitates the formation of the CN structure with a metallic center. Commonly used as a pharmacophore, the 1,3-dioxolane ring regulates the lipophilicity and bioactivity of the Ir(III) complex. The obtained Ir(III) complex in vitro exhibited a potency higher than Cisplatin against NCI-H460, A549, MGC-803, and MDA-MB-231 cell lines (IC_50_ 1.82–2.60 µM vs. IC_50_ 12.23–18.29 µM).

It was demonstrated that iridium(III) complex **90**, by inducing ROS-mediated endoplasmic reticulum stress, can trigger autophagy-dependent ferroptosis and ferroptosis-dependent ICD response in the MDA-MB-231 cell line. Compound **90** has also been found to be a more effective ICD inducer than Oxaliplatin in an in vivo metastatic cancer vaccination murine model. This result confirmed that **90** could significantly alter in vivo the immune microenvironment. By vaccinating BALB/c mice with TNBC cells treated with **90**, a CD8+ T-cell response and Foxp3+ T-cell depletion were observed, and long-lasting antitumor immunity was induced. In a tumor growth experiment, the iridium(III) complex showed a higher anticancer activity and lower toxicity than Oxaliplatin. Moreover, the combination of **90** and anti-PD1 therapy significantly enhanced in vivo therapeutic effects.

**Figure 93:**
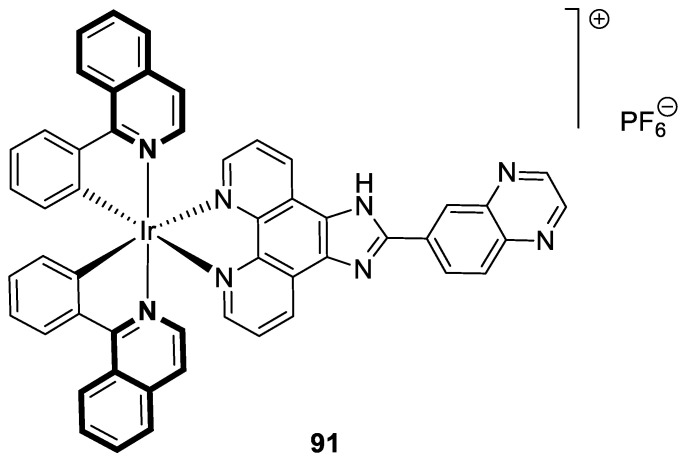
The Ir(III) complex **91** based on 1-phenylisoquinoline and 2-(quinoxalin-6-yl)-1*H*-imidazo[4,5-*f*][1,10]phenanthroline.

Similarly, Liao and colleagues synthesized and studied the activity of a novel cyclometalated iridium(III) complex **91** (Figure 93) based on 1-phenylisoquinoline and the ancillary ligand 2-(quinoxalin-6-yl)-1*H*-imidazo[4,5-*f*][1,10]phenanthroline, derived from 1,10-phenanthroline-5,6-dione and quinoxaline-6-formaldehyde [278].

Upon co-incubation with liver cancer cells (HepG2 line), complex **91** was found to induce paraptosis as well as endoplasmic reticulum stress (ER stress). As a result of ER stress, CD8+ co-receptors were exposed to the surface T cells; high mobility group box 1 protein (HMGB1) and ATP were released into the extracellular space. In addition, **91** induced the immunogenic cell death of the human liver cancer cell line (HepG2) without altering the cell cycle or reactive oxygen species. Upon treatment with **91**, the maturation of dendritic cells was accelerated, effector T cells were more likely to migrate to tumor tissue sites, and immune suppression was alleviated by reducing the proportion of Treg cells in tumor tissues. It is worth noting that **91** did not increase cellular ROS, as other metal complexes with ICD effects have—e.g., Oxaliplatin.

### 7.2. Anti-Alzheimer Agents

Both symmetrical and asymmetrical Schiff bases bearing a di-imine moiety, due to the metal ions’ chelating properties, constitute compounds with promising pharmacological properties. To date, a large group of active metal complexes based on these ligands has been reported [279,280].

**Figure 94:**
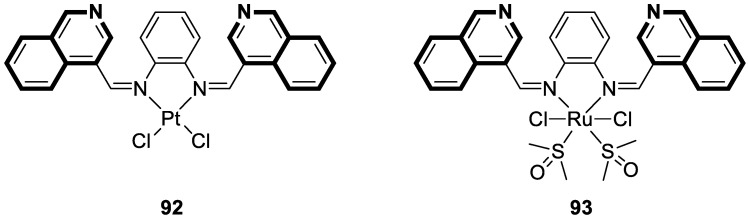
Platinum and ruthenium complexes **92** and **93** with anti-AD activity.

The treatment of the Schiff base—(*N*^1^E,*N*^2^E)-*N*^1^,*N*^2^-bis(isoquinolin-4-ylmethylene)benzene-1,2-diamine—with *cis*-dichloro-bis(dimethylsulfoxido)platinum(II) and dichlorotetrakis-(dimethylsulfoxide)ruthenium(II) in toluene or carbon tetrachloride, at a molar ratio of 1:1, furnished two mononuclear metal complexes bearing di-imine skeletons, **92** and **93** (Figure 94) [281].

Interestingly, the synthesized complexes **92** and **93** display the ability to inhibit in vitro amyloid beta (Aβ_1–42_) aggregation in a dose-dependent manner in the SH-SY5Y cell line. The interaction between the complexes and amyloid beta (Aβ_1–42_) was tested at different concentration ranges. The most effective interaction was observed when the complex/β-amyloid ratio equaled 1:1 and the concentration of **92** or **93** and Aβ_1–42_ was 20 μM. It was demonstrated that both complexes at a concentration of 10 μM were nontoxic to the SH-SY5Y cell line, which shows a high cellular viability. Moreover, it was proven that both complexes displayed optimal lipophilic properties, and therefore they could cross the blood–brain barrier. Complexes **92** and **93** may constitute promising anti-amyloid agents to prevent amyloid aggregation and reduce amyloid cytotoxicity.

### 7.3. Photosensitizers

In the field of photosensitizers, porphyrin-based compounds with intense near-infrared (NIR) absorption are currently being investigated. During near-infrared photodynamic therapy (NIR-PDT), such compounds generate reactive oxygen species (ROS), which may lead to cancer cell death. Typically, many porphyrins can absorb visible light; therefore, research focuses on modifying their structure to develop NIR-absorbing compounds. Moreover, to increase the targeting of cancer and the effectiveness of treatment, efforts are currently being made to combine these compounds with specialized nanocarriers [282].

**Figure 95:**
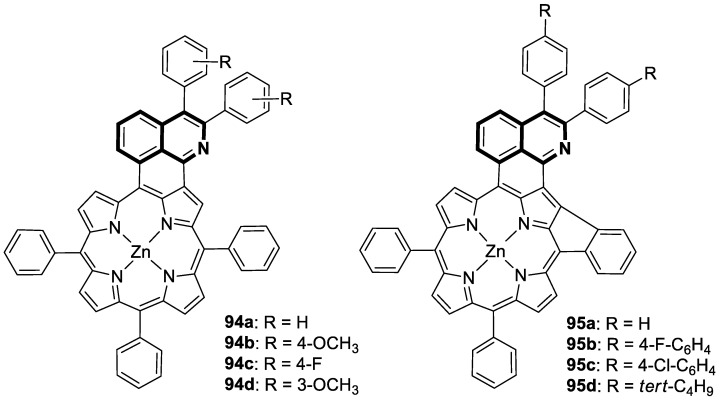
Isoquinoline-based porphyrins **94a**–**d** and **95a**–**d**, designed for near-infrared photodynamic anticancer treatment.

Li and colleagues disclosed the synthesis of isoquinoline-fused porphyrins **94** and **95** (Figure 95), with an impressive NIR absorbing capacity, through a direct rhodium-catalyzed [4+2] cycloaddition reaction [283].

For compounds **94a**–**d** in the DMSO solution, a strong light absorption in the near-infrared (NIR) region (an intense NIR Q-band) was observed. Due to the expanded π-conjugation system of derivatives **95a**, **95b**, **95c**, and **95d**, the recorded Q-bands were red-shifted. In particular, in the absorption spectrum of compound **95a**, red-shifted Soret bands at λ = 527 nm and λ = 554 nm were visible, accompanied by broad Q-bands extending to 1000 nm. Due to the remarkable singlet oxygen generation properties of porphyrin type **94** upon NIR laser (0.24 W/cm^2^, 10 min) irradiation in the phototherapeutic window (λ = 660 nm), the representative nanoparticles assembled (**94a**-NPs) were examined in HeLa cells, displaying excellent tumoricidal activity and good biocompatibility. The compound showed cytoxicic properties in a concentration-dependent manner, with a calculated half-maximal inhibitory value of 35 μg/mL (IC_50_ = 39 μM).

## 8. Conclusions

Isoquinolines are a significant class of heterocyclic aromatic organic compounds composed of a benzene ring fused to a pyridine ring. Among the abundant groups and classes of heterocyclic compounds, the isoquinoline scaffold is recognized as a promising privileged structure for drug development.

In nature, isoquinoline forms the structural backbone of many alkaloids known as isoquinoline-based alkaloids—widely distributed in plants, particularly in species of the families *Papaveraceae*, *Berberidaceae*, *Ranunculacae*, and *Menispermaceae*. These natural products include pharmacologically valuable compounds such as Papaverine, a spasmolytic agent, or Morphine, a well-known analgesic derived from the opium poppy (*Papaver somniferum*). Berberine—in traditional Chinese medicine—was used as an antimicrobial and anti-inflammatory drug. Apart from Berberine, isoquinoline-based alkaloids such as Epiberberine, Palmatine, Coptisine, Sanguinarine, and Chelerythrine display antioxidant, antifungal, or antiviral properties. The diversity and complexity of isoquinoline alkaloids make them a fascinating subject for study in natural product chemistry and pharmacognosy.

From a synthetic point of view, numerous methods have been developed to efficiently produce functionalized isoquinoline derivatives with potential biological activities. Isoquinoline and its derivatives play a crucial role in various industrial and pharmaceutical applications. In the synthesis of dyes, pesticides, and specialty chemicals, they frequently serve as major intermediates.

In the context of drug discovery, a wide range of their diverse pharmacological properties has been discovered. Isoquinoline derivatives are claimed to be particularly promising agents in cancer treatment. Several synthetic compounds have shown promise as inhibitors of enzymes and receptors, exerting an array of antiproliferative mechanisms. Isoquinoline derivatives have shown promise as inhibitors of enzymes or receptor modulators involved in Alzheimer’s disease and inflammatory or diabetic disorders. They also possess good antibacterial, antifungal, antiviral, and antiprotozoal properties. Therefore, isoquinoline-based compounds may find potential applications in medicine as candidates for effective therapeutic agents used in the treatment and prophylaxis of Human Immunodeficiency Virus, hepatitis B virus, or influenza infections.

Furthermore, isoquinoline-based compounds are also valuable in materials science due to their ability to form stable chelates with different metals. Due to the presence of a donor-character aromatic nitrogen atom, an isoquinoline core can function as an appropriate ligand for the synthesis of stable metal complexes. The metal-coordinated isoquinolines exhibit diverse pharmacological activities and have recently attracted attention as promising drug candidates. In comparison with free ligands, these metal chelates may exert a higher potency. Moreover, several complexes demonstrate unique optical and electronic properties, which are advantageous for the development of fluorescent-labeled probes or other biomedical electronic materials. In the field of photosensitizers, isoquinoline-fused zinc-containing porphyrins display an impressive NIR absorption capacity.

In summary, isoquinolines represent a versatile and scientifically significant group of nitrogen-containing heterocyclic compounds. Their rich chemistry, diverse biological activities, and broad range of practical applications continue to attract attention from chemists, pharmacologists, and material scientists. The study of isoquinolines not only contributes to a deeper understanding of heterocyclic chemistry but also provides valuable insights into the development of novel drugs and functional materials. On the other hand, several challenges persist. Many existing studies still do not provide thorough assessments of safety or pharmacokinetic profiles, and the mechanisms underlying the activity of numerous isoquinoline-based compounds are limited. A more detailed understanding of their molecular targets and modes of action is crucial for fully exploiting their therapeutic potential. Furthermore, the use of advanced computational modeling and quantitative structure–activity relationship (QSAR) approaches is expected to support the development of isoquinoline derivatives with a greater target selectivity, reduced toxicity, and improved pharmacokinetic properties. The development of appropriate formulations may also enhance the bioavailability and therapeutic index of these molecules. Despite these ongoing challenges, the progress made across diverse applications underscores the importance of the isoquinoline scaffold as a privileged structure in modern drug design. Examples of valuable and promising isoquinoline-based compounds and their metal complexes discussed in this review are presented in Table 1 and Table 2.

## Figures and Tables

**Table 1:** Examples of valuable isoquinoline-based compounds, their biological activities and molecular targets.

Structure	Biological Activity	Molecular Target	Name/ Number	Ref.
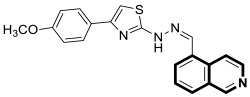	Anticancer	Akt	**1a**	[34]
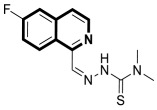	Anticancer	Oxidative stress, mitochondrial-dependent toxicity, oxidative phosphorylation (OXPHOS)	**2**	[38]
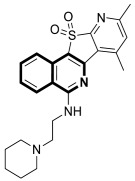	Anticancer	Topo I	**4**	[40]
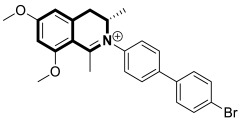	Anticancer	PI3K/Akt/mTOR signaling pathway	**Toyaburgine**	[51]
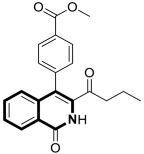	Anticancer	MAPK/ERK or Ras-Raf-MEK-ERK and p38 MAPK signaling pathways, caspase-3 clevage of GSDME	**8**	[52]
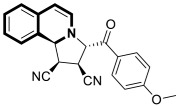	Anticancer	α,β-tubulin colchicine binding site	**9a**	[53]
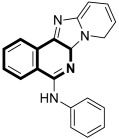	Anticancer	Apoptosis via mitochondrial dysfunction, caspase-3 and PARP-1	**10a**	[58]
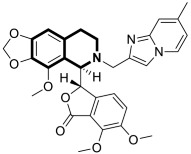	Anticancer	Apoptosis, G2/M phase	**12**	[60]
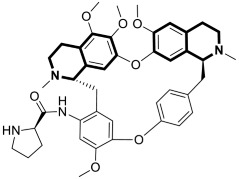	Anticancer	Autophagy, anti-angiogenic effect	**13**	[71]
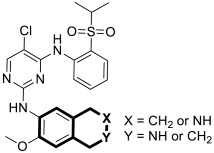	Anticancer	ALK	**16a-b**	[81]
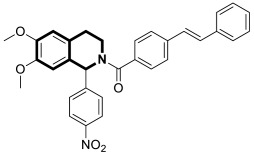	Anticancer	G2/M phase, mitochondrial-dependent apoptotic pathway	**17**	[82]
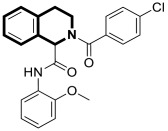	Anticancer	NF-κB signaling pathway	**18**	[83]
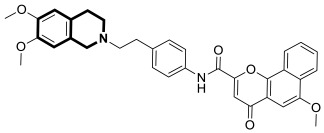	Anticancer	gp-P and CYP1B1	**20**	[87]
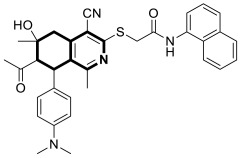	Anticancer	G2/M phase, CDK2	**22**	[90]
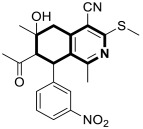	Anticancer	Apoptosis, G0-G1 and G2/M phases, HSP90	**24**	[91]
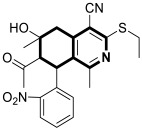	Anticancer	G2/M phase, RET	**26a**	[92]
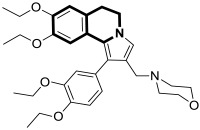	Anticancer	P-gp/MRP1	**31**	[99]
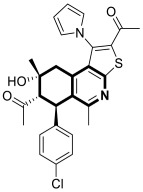	Anticancer	G2/M phase, cellular apoptosis, tubulin polymerization	**35**	[103]
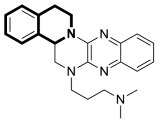	Anticancer	PPARγ, AKT	**37**	[107]
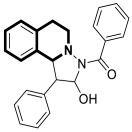	Anticancer	MAPKs signaling pathway	**LFZ-4-46**	[108]
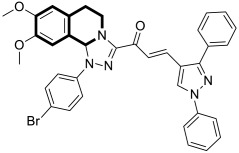	Anticancer	EGFR	**39a**	[115]
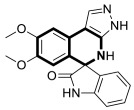	Anticancer	IL-6	**40**	[119]
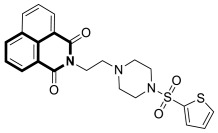	Anticancer	Carbonic anhydrase isoform IX (CAIX)	**41**	[120]
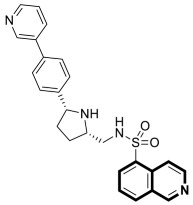	Antibacterial	DNA-gyrase	**LEI-800**	[143]
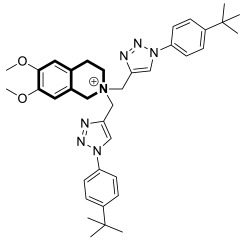	Antibacterial	*S. aureus*, *B. subtilis*, *M. tuberculosis* H37Rv	**45a**	[145]
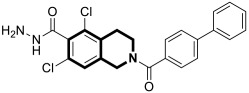	Anti-mycobacterial	Enoyl reductase 4TZK	**49**	[152]
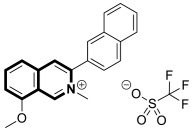	Antifungal	*A. solani, A. alternata, P. piricola*	**50**	[156]
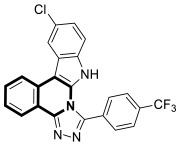	Antimicrobial	*S. aureus, B. subtilis, E. coli*, *C. albicans*, *F. oxysporum*, *A. flavus*, supercoiled DNA	**53**	[158]
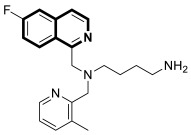	Antiviral	Chemokine receptor CXCR4, HIV-1 and HIV-2	**54**	[164]
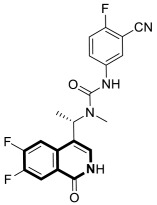	Antiviral	HBV capsid assembly	**AB-836**	[167]
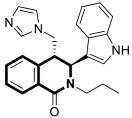	Antiviral	Coronavirus OC-43 and 229	**56**	[173]
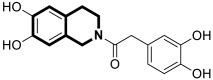	Antiviral	Influenza A, PA_N_ endonuclease	**57**	[176]
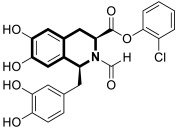	Antiviral	Influenza A, PA_N_ endonuclease	**58**	[177]
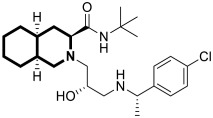	Antiviral	Ebola virus glycoprotein (EBOV-GP)	**59**	[179]
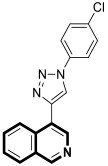	Antimalarial	*P. falciparum 3D7* and *K1*	**62**	[184]
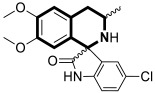	Antimalarial	*P. falciparum 3D7*	**(±)-64**	[189]
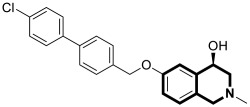	Anti-trypanosomal	*T. b. rhodesiense*	**65**	[192]
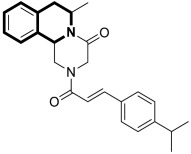	Antischistosomal	*S. mansoni*	**71**	[202]
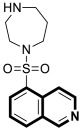	Anti-Alzheimer’s disease activity	ROCK	**Fasudil**	[211,212]
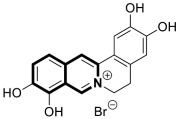	Anti-Alzheimer’s disease activity	Modulation of mitophagy	**73b**	[218]
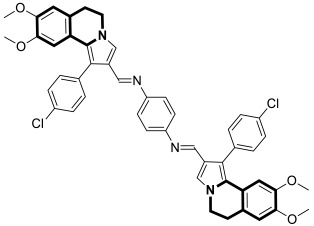	Anti-Alzheimer’s disease activity	AChE, MAO A, Aβ40	**75**	[223]
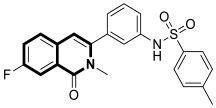	Anti-inflammatory	PDE-4, TNF-α	**76b**	[238]
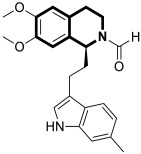	Anti-inflammatory	PDE-4	**DC** **591017**	[241]
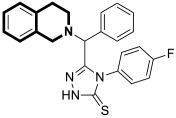	Anti-inflammatory	COX-2	**78a**	[245]
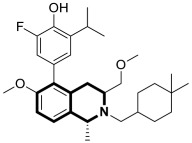	Antidiabetic	GLP-1R, GIPR	**81**	[247]
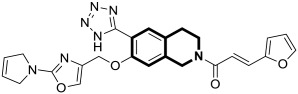	Antidiabetic	PPARγ	**82**	[253]
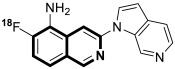	*tau* PET tracer	Neurofibrillary tangles (NFTs)	**[^18^F]MK-6240**	[258]
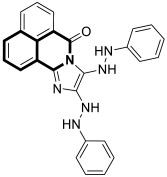	Fluorescent sensor	Copper(II), iron(III), and chloride ions	**83**	[260]
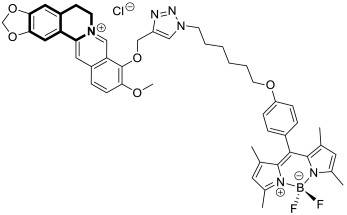	Fluorescent sensor	MCF-7 human breast carcinoma cells	**BBR-BODIPY**	[261]

**Table 2:** Examples of valuable metal complexes of isoquinoline-based compounds, their biological activities and molecular targets.

Structure	Biological Activity	Molecular Target	Name/ Number	Ref.
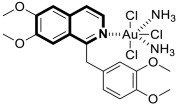	Anticancer	MCF-7 and Hep-G2 cell lines	**85**	[265]
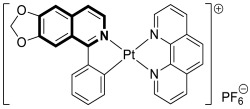	Anticancer	Ferritinophagy-dependent ferroptosis, nuclear receptor coactivator 4 (NCOA4)	**86**	[266]
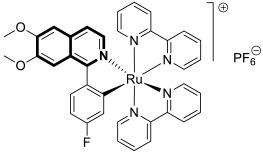	Anticancer	ROS-mediated apoptosis, nuclear factor erythroid factor 2 (Nrf2)	**87c**	[272]
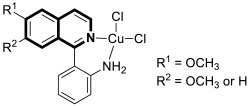	Anticancer	Mitochondrial-mediated apoptosis, MAPK signaling pathway, autophagy	**88a** **–** **b**	[273]
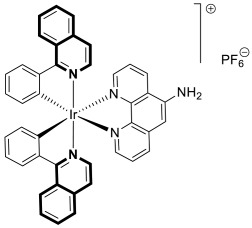	Anticancer	Apoptosis via ER targeting, immunogenic cell death (ICD), ROS production	**89**	[275]
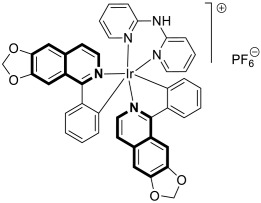	Anticancer	Autophagy-dependent ferroptosis, ferroptosis-dependent ICD	**90**	[277]
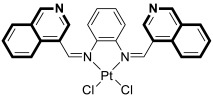	Anti-Alzheimer’s disease activity	Amyloid beta (Aβ_1-42_) aggregation	**92**	[281]
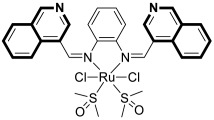	Anti-Alzheimer’s disease activity	Amyloid beta (Aβ_1-42_) aggregation	**93**	[281]
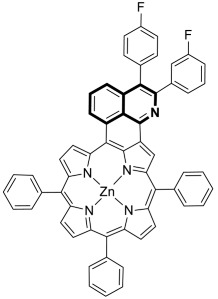	Photosensitizer	Near-infrared photodynamic therapy (NIR-PDT)	**94c**	[283]

## Data Availability

Not applicable.
